# Fragment-Based Structural Optimization of a Natural Product Itampolin A as a p38α Inhibitor for Lung Cancer

**DOI:** 10.3390/md17010053

**Published:** 2019-01-12

**Authors:** Jing-wei Liang, Ming-yang Wang, Shan Wang, Xin-yang Li, Fan-hao Meng

**Affiliations:** School of Pharmacy, China Medical University, Liaoning 110122, China; liangjw89@163.com (J.-w.L.); wmy940623@163.com (M.-y.W.); 18341653600@139.com (S.W.); 13125424162@163.com (X.-y.L.)

**Keywords:** itampolin A, FBDD, antitumor, p38α, novel inhibitor

## Abstract

Marine animals and plants provide abundant secondary metabolites with antitumor activity. Itampolin A is a brominated natural tyrosine secondary metabolite that is isolated from the sponge *Iotrochota purpurea*. Recently, we have achieved the first total synthesis of this brominated tyrosine secondary metabolite, which was found to be a potent p38α inhibitor exhibiting anticancer effects. A fragment-based drug design (FBDD) was carried out to optimize itampolin A. Forty-five brominated tyrosine derivatives were synthesized with interesting biological activities. Then, a QSAR study was carried out to explore the structural determinants responsible for the activity of brominated tyrosine skeleton p38α inhibitors. The lead compound was optimized by a FBDD method, then three series of brominated tyrosine derivatives were synthesized and evaluated for their inhibitory activities against p38α and tumor cells. Compound **6o** (IC_50_ = 0.66 μM) exhibited significant antitumor activity against non-small cell lung A549 cells (A549). This also demonstrated the feasibility of the FBDD method of structural optimization.

## 1. Introduction

Sponges are the most abundant source of bioactive compounds in marine invertebrates [1]. About half of the current FDA-approved marine drugs come from sponges or derivatives based on sponge metabolites [2,3,4]. Most of the natural products isolated from sponges belong to bromotyrosine derivatives and exhibit extensive biological activities [5,6,7,8]. Itampolin A was the first brominated tyrosine alkaloid isolated from the sponge *Iotrochota purpurea* [9]. In our previous study, the total synthesis of itampolin A was achieved for the first time. In an evaluation of the pharmacological properties, the alkaloid showed potent concentration-dependent antitumor activity against non-small cell lung cancer. In addition, its enantiomer (−)-itampolin A exhibited significant inhibitory activity against non-small cell lung A549 cells. The underlying anticancer mechanism was associated with its binding to DFG-out conformation of p38α and then decreasing the phospho-p38 expression in a time-dependent manner [10].

In recent years, the design of more selective kinase inhibitors, targeting inactive conformations, has been explored. Relative to type I inhibitors that compete directly with ATP, type II inhibitors bind to the inactive DFG-out conformation of kinase induced by the conformational transition of DFG-loop. This opened up a second hydrophobic subcavity formed by the catalytic amino acid triad Asp168, Phe169, and Glu71. A number of studies have demonstrated the advantages of targeting the DFG-out binding mode of kinases in general and p38 MAP kinase (p38 MAPK) in particular such as low toxicity [11].

Fragment-based drug design (FBDD) is now widely used in academia and industry to obtain small molecule inhibitors for a given target. Moreover, it is established for many fields of research including antimicrobials and oncology [12,13,14]. Many molecules derived from fragment-based approaches are already in clinical trials and two—Vemurafenib and Venetoclax—are on the market [13]. Unlike other computer aided drug design (CADD) methods, the FBDD theory maintains that the active pockets of the drug target are made up of multiple subcavities, and the fragments are units that combine with these subcavities. Finding these fragments and linking them together often leads to higher active compounds [15].

For the purpose of improving the activity of itampolin A, and increasing the structural diversity of type II inhibitors, we here reported the optimization of (−)-itampolin A as novel p38α inhibitors by using the FBDD method. This strategy involved interrogation of structural information that was available for different in-house chemotypes [16]. The challenge included three aspects. The first one was deconstruction of known p38α inhibitors to identify highly efficient interactions in the binding site. The second one was screening out suitable units that fit the second hydrophobic subcavity. The last one was exploring the effects on the activity of some atom or fragment substitutions of the brominated tyrosine skeleton.

## 2. Results

### 2.1. Fragment-Based Drug Design

**Figure 1:**
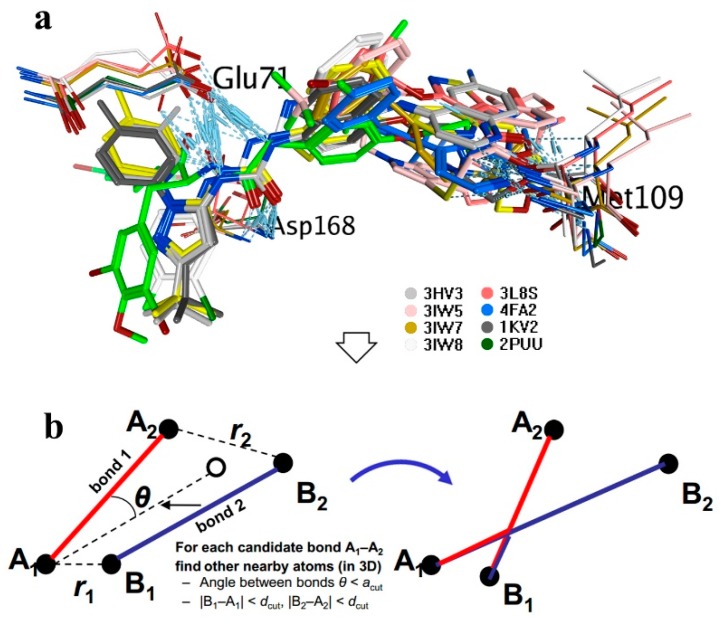
Workflow of the BREED method; (**a**) The lead compound (green) overlapped with type II inhibitors; (**b**) ligand fragmentation based on interatomic distance and bonding angle.

The conformation of inactive p38α bound with type II inhibitors was screened out from the PDB website as 3HV3, 3IW5, 3L8S, 3IW7, 3IW8, 4FA2, 2KV2, and 2PUU. The conformation of itampolin A overlapped with BIRB-796 was obtained in a previous work. The above conformations were superposed together after alignment (Figure 1a). An FBDD-based BREED technique was adopted as a novel fragment-based drug design method, which was based on sets of aligned 3D ligand structures binding to the same target or target family. The implementation comprises two steps. Firstly, a superposition of ligands (Figure 1a). Secondly, a ligand fragmentation based on interatomic distance and bonding angle. This was followed by a scoring scheme assigning individual scores to each fragment, and the incremental construction of novel ligands based on a greedy search algorithm guided by the calculated fragment scores (Figure 1b). These small molecules were then screened by pharmacophore models and the lipinsiki rule of five. The BREED results generated by MOE software are described in the Supplementary Materials, Table S1.

### 2.2. Synthesis

**Scheme 1:**
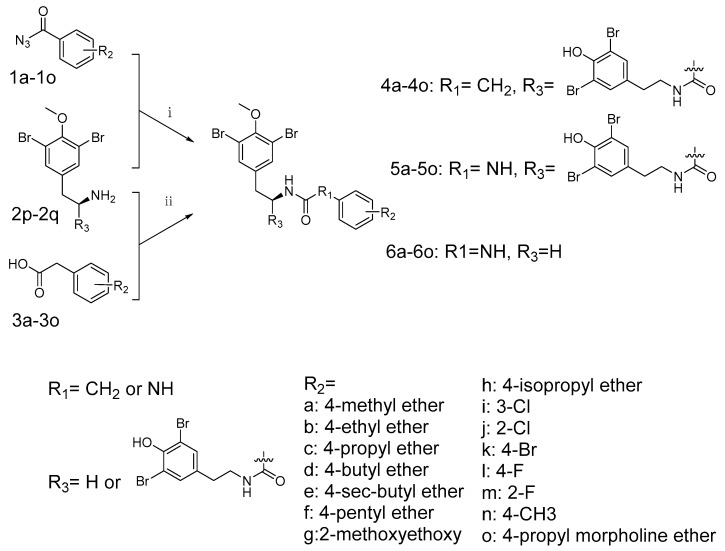
Synthesis of novel brominated tyrosine derivatives. Conditions: (**i**) 1.1 eq. 1a–1o, DCE, 80 °C, 39–51%; (**ii**) 1.05 eq. 2p, 2q, EDCI, HOBt, Et3N, DMF, rt., 52–57%.

The synthesis of brominated tyrosine derivatives followed the synthetic route outlined in Scheme 1. The chemical synthesis method to access the parent compounds (+)-itampolin A and (−)-itampolin A were reported previously, as well as 2p, 2q and 3a–3o [10]. The other important intermediates 1a-1o for synthesizing the brominated tyrosine derivatives were substituted benzoyl azide. Thearomatic hydrazines were used as raw material to obtain aryl azides by diazotization. The substituted benzoyl azides produced corresponding substituted isocyanatobenzenes through Curtius rearrangement in DCE at 80 °C.

### 2.3. Inhibitory Activities of (−)-Itampolin A Skeleton Brominated Tyrosine Derivatives

The capacity of the synthesized brominated tyrosine derivatives for inhibiting p38α activity was evaluated. The evaluation results are summarized in Table 1.

**Figure 2:**
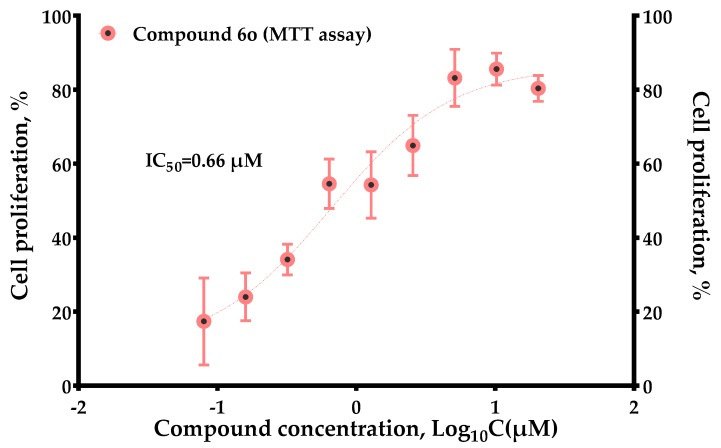
Dependence of concentration versus the activity of compound **6o**.

In the assay of inhibiting p38α activity, the compound **6o** exhibited the best activity. The compoundwas selected for further cell proliferation inhibition experiments by MTT assay. Because the lead compound (−)-itampolin A showed an inhibitory effect on A549, this cell line was still selected to evaluate **6o** activity. Compound **6o** inhibited A549 cell proliferation in a concentration-dependent manner with IC_50_ = 0.66 μM. The experimental results are shown in Figure 2.

### 2.4. 3D-QSAR Study

In order to clarify the structure–activity relationship between the bromine tyrosine derivatives and antitumor activity, a 3D-QSAR study on the derivatives was carried out using the CoMFA and CoMSIA method in Sybyl-X2.0.

**Figure 3:**
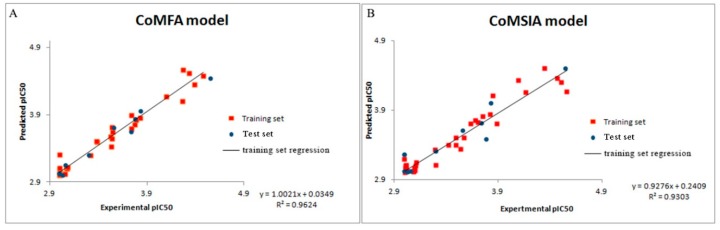
Experimental versus predicted activity of the training and test set based on the Topomer CoMFA model (**A**) and CoMSIA model (**B**).

The statistical results of the Topomer CoMFA model were as follows: q^2^ value of 0.700; r^2^ value of 0.954, with five optimum components. With the optimal number of components being 4 in the CoMSIA model, q^2^, r^2^, and SEE were found to be 0.615, 0.897, and 0.124 respectively. The statistical results proved that the QSAR model of Topomer CoMFA and CoMSIA has precise predictability. The experimental and predicted activities of both the training set and test set are shown in Figure 3. The Topomer CoMFA and CoMSIA models gave the correlation coefficient (r^2^) value of 0.9271 and 0.909, respectively, which demonstrated the internal robustness and external high prediction of the QSAR models.

**Figure 4:**
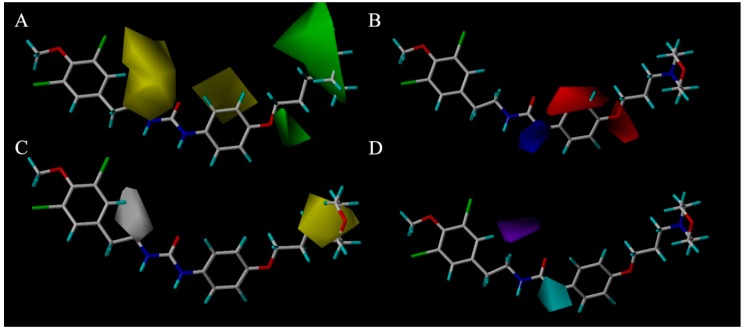
(**A**) Steric contour maps of Topomer CoMFA around the brominated tyrosine skeleton. Green contours refer to sterically favourable regions and yellow contours refer to sterically unfavorable regions. (**B**) Electrostatic contour maps of Topomer CoMFA around the brominated tyrosine skeleton. Red contours refer to regions where electropositive groups were favorable and blue contours refer to regions where electropositive groups were unfavourable. (**C**) Hydrophobic field contour maps for CoMSIA. Yellow contours represent regions where hydrophobic substituents were favorable, white contours represent regions where hydrophilic substituents were unfavourable. (**D**) H-bond donor field contour maps for CoMSIA. Cyan contours indicate regions where hydrogen bond donor substituents increase activity, and purple contours indicate the unfavorable regions for hydrogen bond donor groups.

A 3D-QSAR contour map was utilized to exhibit the Topomer CoMFA model and CoMSIA model properties in 3D-space, as well as to obtain information in ligand-receptor conformation. After the visualization of favorable and unfavorable regions of fields in a 3D-QSAR contour map, several fields (steric fields, electrostatic fields, hydrophobic fields, hydrogen bond donor atom fields, and hydrogen bond acceptor atom fields) were used to realize the relationship between the biological activities and structures. Steric and electrostatic contour maps of the Topomer CoMFA QSAR model are shown in Figure 4a,b respectively. HBA and hydrophobic contour maps of the CoMSIA QSAR model are shown in Figure 4c,d respectively. Compound **6o** has the most complex chemical structure for the visual clarity of analyzing the QSAR, hence it was chosen as the reference structure for the generation of the Topomer CoMFA and CoMSIA contour map.

## 3. Discussion

**Figure 5:**
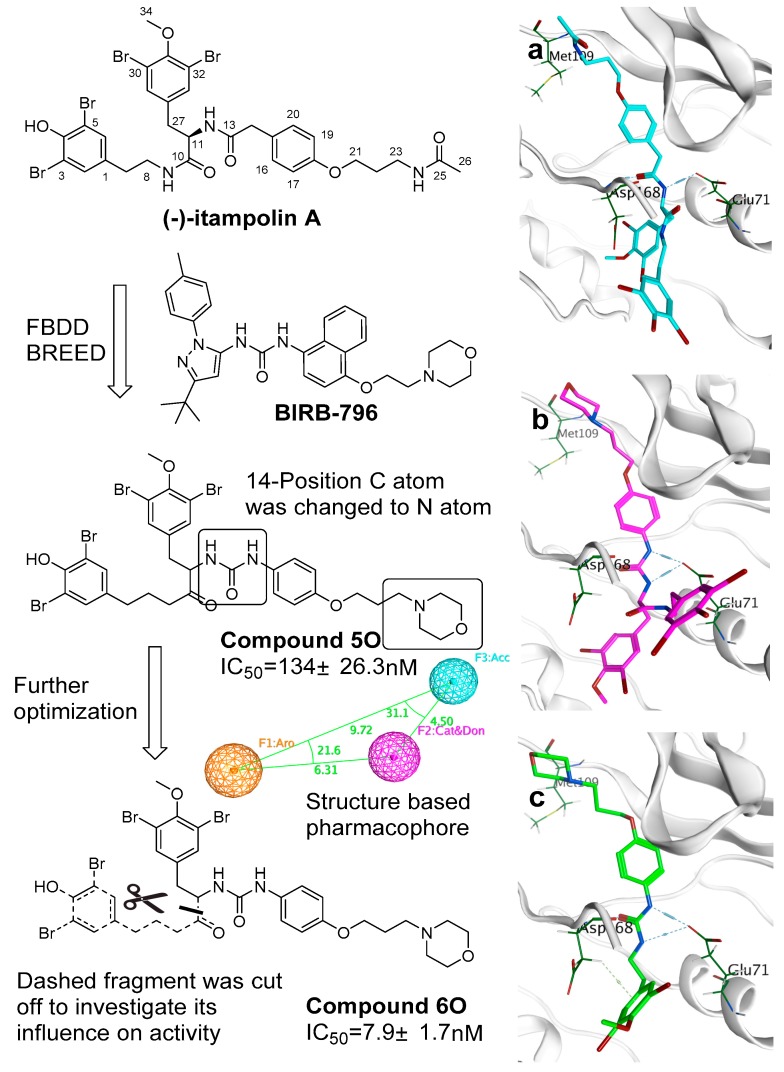
Structural optimization of (−)-itampolin A; (**a**) the conformation of (−)-itampolin A docked to p38α; (**b**) the conformation of compound **5o** docked to p38α; (**c**) the conformation of compound **6o** docked to p38α.

Based on the aligned 3D ligand structures binding to p38α, a novel skeleton was generated based on the structure of (−)-itampolin A (Figure 5). The carbon atom of 14-position was changed to a nitrogen atom, making the amide unit (12-position, 13-position) convert to a disubstituted urea group. As a result, the new skeleton was capable of binding Glu71 residue. It stabilized the conformation of the molecule in the active site. This binding was crucial, because the interaction between the urea unit and Glu71 ensured that bromoaromatic groups enter the second hydrophobic pocket unique to DFG-out conformation. The atoms between 23-position and 26-position were replaced with a morpholine unit (there were also other units such as fluorine-substituted aromatic ring that are listed in Table S1). The urea group probably came from the inhibitors which contained the urea unit. The morpholine unit came from BIRB-796. The fluorine-substituted aromatic came from the inhibitor of 3IW5 or 4FA2. Most inhibitors supported the change to the urea unit. However, each inhibitor supported different changes in the atoms between 23-position and 26-position (Figure 5 shows a more active one with the morpholine unit). Therefore, it was suggested that it is necessary to explore more units to replace the atoms between 23-position and 26-position. The results of molecular docking showed that most of the brominate tyramine fragment was exposed outside the active pocket. Moreover, excessive hydrophobic fragments might hinder the binding of the molecule to Glu71 (Figure 5a). After the discovery of defects in the skeleton, a filter was added to iterate the process of FBDD. This filter included the Lipinski’s rule of five and a structure-based pharmacophore, worked out by MOE 2015. The screening results showed that cutting off the fragment can further improve the activity of the compounds with a novel skeleton.

In the case of CoMFA analysis, the contour maps around **6o** (Figure 4a,b) were generated from the CoMFA model. The steric contour map of **6o** is shown in Figure 4a. Two green regions near the *N*-substituents of the morpholine proved that this length of hydrocarbon chain preferred sterically favorable functional groups. In contrast, a yellow region over the 11-C showed that a large group such as segment II leads to a decrease in activity. Consistent with this result, after cutting off the brominate tyramine fragment, the activities of compounds 3a–3o were found to be improved. This may be attributable to the excessive volume of the fragment. It was exposed to the outside of the active site pocket, when (−)-itampolin A was docked into p38α, as shown in Figure 5a. The electrostatic contour map of **6o** is shown in Figure 4b. The red region over the benzene ring revealed that substituents on the benzene ring preferred electronegative groups, such as halogen atoms. The blue region around the 14-N showed that electropositive groups possessed good anticancer activity. Consistent with the QSAR analysis result, the molecular docking study showed that the urea group forms one more hydrogen bond to Glu71 than the amide group (Figure 5b). These results explained the fact that almost all of the compounds in the second series exhibited more potent inhibitory effects than those in the first series after the 14-position carbon atom was changed to a nitrogen atom.

The CoMSIA contour hydrophobic map is shown in Figure 4c. The yellow contour map in the morpholine indicated the region where addition of the hydrophobic groups would increase the inhibitory activity while the white contour map over the 11-C proved that segment II in this position will decrease the inhibitory activity. The contour map of the CoMSIA hydrogen bond donor (HBD) is shown in Figure 4d; the purple region above 11-C represents the HBD atom substitution which was adverse to the activity. This might be attributed to the hydroxyl of segment II. The cyan region around the 14-N atom indicates that the addition of the HBD atom was conducive to the activities. The CoMSIA HBD contour map can be validated by the fact that almost all of the compounds in the second series exhibited more potent inhibitory effects than those in the first series after the 14-position carbon atom was changed to a nitrogen atom.

**Figure 6:**
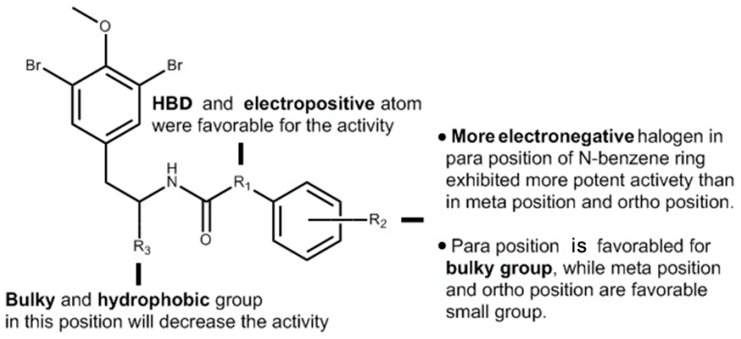
Important structural requirements of the novel brominated tyrosine skeleton by means of the ligand-based 3D-QSAR and structure-based molecular docking study.

Finally, we combined the ligand-based 3D-QSAR analysis with the structure-based molecular docking study, to identify the necessary moiety of the brominated tyrosine derivatives (Figure 6).

In conclusion, (−)-itampolin A, as a lead compound, was optimized by the FBDD method based on the interaction between type II inhibitors and p38α. The 14-position carbon atom was changed to a nitrogen atom, making the amide group change to a diaryl urea unit. The brominate tyramine fragment and the substituent groups on the benzene ring were also modified. Thus, 45 brominated tyrosine derivatives of three series were designed and synthesized. Not all the compounds were reported in previous studies, and their structures were confirmed by ^1^H-NMR and ^13^C-NMR. Through the analysis of 3D-QSAR, it was found that the conversion of the 14-position carbon atom to a nitrogen atom contributed to improving the activity of the derivatives. Cleavage of the brominate tyramine fragment helped to improve the activities of **6a**–**6o**. The groups on the benzene ring preferred electron-withdrawing groups, followed by meta-substituted halogen atoms. The alkane chains on the aromatic ring were not beneficial to increasing inhibitory activity. Moreover, we discovered that a novel derivative, **6o** (IC_50_ = 0.66 μM), exhibited significant antitumor activity against A549.

## 4. Experimentation

### 4.1. Fragment-Based Drug Design

Fragment-based drug design was performed using the BREED module in Molecular Operating Environment package (MOE 2015.1001.). Eight molecules (ligands in 3HV3, 3IW5, 3L8S, 3IW7, 3IW8, 4FA2, 2KV2, 2PUU) after alignment were set as novel bond producers. (−)-Itampolin A was set as the parent compound. During the generation, a filter included the Lipinski’s rule of five and a structure-based pharmacophore was toggled ON. The novel compounds were generated based on candidate bonds.

### 4.2. Molecular Docking

Molecular docking was performed using MOE. The ligands were docked into p38α after energy minimization. Their conformations were generated with the bond rotation method. In order to validate the docking protocol, a self-docking of BIRB-796 into the binding pocket was firstly performed. The triangle matcher was selected as the ligand placement method, the London dG was selected as the scoring function, the rigid receptor was used as the type of post-placement refinement, the GBVI/WSA dG was used as the refinement scoring function. The one with the best docking conformation was selected from 30 poses according to E-strain. The RMSD value between the docking conformation and actual conformation was 0.9695, as shown in Figure S1.

### 4.3. The p38α MAP Kinase Activity Based on the Rate of Phosphorylation of ATF-2

Kinase reaction buffer composition: 50 mM HEPES (pH 7.5), 10 mM MgCl_2_, 0.01% BRIJ-35 and 1 mM EGTA. Titration MAPK14/p38α at 90 μM ATP: Prepare MAPK14/p38α in kinase buffer at a concentration of 500 ng/mL. Perform two-fold serial dilution using kinase buffer from 500 ng/mL, 16 dose points. Add 5 μL of the serially diluted MAPK14/p38α into the 384-well plate in triplicate. Prepare 1 mL of 0.8 μM substrate GFP-ATF2 (19–96) and 180 μMATP in kinase reaction buffer. The reaction was carried out by adding 5 μL of the substrate GFP-ATF2 (19–96) and ATP solution into each well of the assay plate. Seal the assay plate and incubate for 1 h at room temperature (RT). Add 10 μL of the antibody solution prepared in TR-FRET dilution buffer (20 mM EDTA and 4 nM Tb-antipATF2 (pThr71), and mix gently. The final EDTA and Tb-antipATF2 (pThr71) concentrations were 10 mM and 2 nM, respectively. Seal the assay plate and incubate for 30 min at RT. Read the TR-FRET signal on the Envision 2104 plate reader. 

Determination of IC_50_ values of inhibitors: add 2 μL/well of inhibitor in 0.5% DMSO at five-fold the final assay concentration to the 384-well assay plate. For the first cycle inhibitor screening, the final concentrations of inhibitors were 1117, 279.25, 69.81, 17.45, and 4.36 nM (three-fold dilution, five dose points, two replicates for each dose). Adjust the inhibitor concentration based on the results of the first cycle. An amount of 4 μL MAPK14/p38α was added to each well of the 384-well assay plate. Incubate for 15 min at RT. Finally, 4 μL substrate GFP-ATF2 (19–96) and ATP in kinase reaction buffer were added to start the reaction. The final concentrations of MAPK14/p38α, Substrate, and ATP were 1 ng/mL, 0.4 μM, and 90 μM, respectively. Incubate for 1 h at RT. Add 10 μL/well of antibody solution. The final concentrations of EDTA and antibody were 10 mM and 2 nM respectively. Incubate for 30 min at RT. Read the TR-FRET signal on the Envision 2104 plate reader.

### 4.4. MTT Assay

The A549 cell lines were cultured using DMEM and RPMI, respectively, in standard humidified incubation conditions at 37 °C in 5% CO_2_. Then, the cancer cells (1000/well) were placed on 96-well plates in triplicate. After 24 h incubation, cells were treated with a series of concentrations of the brominated tyrosine derivatives (1, 10, 20, 80 μmol/L). The positive control contained the same concentrations of BIRB-796, and the negative control received the same volume of DMSO. After 48h incubation, cells were stained with 5% MTT, and the optical density was recorded at an absorbance wavelength of 570 nm.

### 4.5. 3D-QSAR Study

The SKETCH function of Sybyl-X2.0 was utilized for drawing the structure and charges were calculated by the Gasteiger–Huckel method, and tripos force field was utilized for energy minimization of these molecules. These 45 synthetic molecules were divided into the training set and test set in the ratio of 80:20. The training set was used to build the 3D-QSAR model, and the test set was used to test the predictions of the model [17,18]. In the case of building the Topomer CoMFA QSAR model, a carbon sp3 probe was applied for calculating steric and electrostatic parameters. The database alignment was used to build the CoMSIA QSAR model [19].

### 4.6. Chemsitry

^1^H-NMR and ^13^C-NMR spectra were recorded on Varian NMR spectrometers operating at 600 MHz for ^1^H, and 150 MHz for ^13^C. All chemical shifts were measured in DMSO-*d*6 as solvent. All chemicals were purchased from Sinoreagent Chemcal Reagent (Beijing, China) and were used as received, unless stated otherwise. Analytical TLC was performed on Haiyang (Qingdao Haiyang Chemcal Co., Ltd. (Qingdao, China)) silica gel 60 F254 plates and visualized by UV and potassium permanganate staining. Flash column chromatography was performed on Haiyang (Qingdao Haiyang Chemcal Co., Ltd.) gel 60 (40–63 mm). HPLC was performed on Agilent 1260 Infinity II. Chromatographic separation was performed on a C18 column (3.0 × 50 mm, 1.7 µm) and the column temperature was maintained at 25 °C. The mobile phase consisted of methanol (50%) and water (50%).

The synthesis methods of the derivatives are described in the Supplementary Materials.

## Figures and Tables

**Table 1:** p38α MAP kinase inhibitory activity of brominated tyrosine derivatives.

No.	IC_50_ (nM)	No.	IC_50_ (nM)	No.	IC_50_ (nM)
4a	--	5a	112.3 ± 12.0	6a	262.7 ± 3.3
4b	492.5 ± 46.2	5b	346.4 ± 18.4	6b	221.4 ± 4.2
4c	--	5c	167.2 ± 23.9	6c	701.4 ± 7.4
4d	805.6 ± 139.4	5d	690.6 ± 38.4	6d	--
4e	--	5e	--	6e	920.3 ± 6.1
4f	--	5f	--	6f	--
4g	214.8 ± 64.0	5g	223.4 ± 10.3	6g	11.7 ± 3.0
4h	--	5h	--	6h	329.4 ± 44.2
4i	91.0 ± 11.1	5i	51.3 ± 4.5	6i	17.5 ± 2.4
4j	--	5j	447.7 ± 64.1	6j	71.2 ± 4.5
4k	832.1 ± 12.1	5k	90.3 ± 2.7	6k	169.0 ± 2.6
4l	617.7 ± 87.7	5l	110.4 ± 18.8	6l	21.5 ± 4.6
4m	78.6 ± 59.2	5m	71.4 ± 19.6	6m	13.6 ± 3.0
4n	--	5n	278.6 ± 28.9	6n	299.6 ± 11.7
4o	137.5 ± 13.6	5o	134.2 ± 26.3	6o	7.9 ± 1.7
				BIRB-796	11.3 ± 0.2

## Supplementary Materials

The following are available online at http://www.mdpi.com/1660-3397/17/1/53/s1. Figure S1: Comparison of the BIRB-796 molecular docking conformation (green) with the actual conformation (blue).
