# Supplementary material for: Fragment-Based Structural Optimization of a Natural Product Itampolin A as a p38α Inhibitor for Lung Cancer
Source: Mar Drugs. 2019 Jan 12;17(1):53. doi: 10.3390/md17010053 (PMC6356581; doi:10.3390/md17010053)
Supplement: Supplementary file 1 [file marinedrugs-17-00053-s001.pdf]

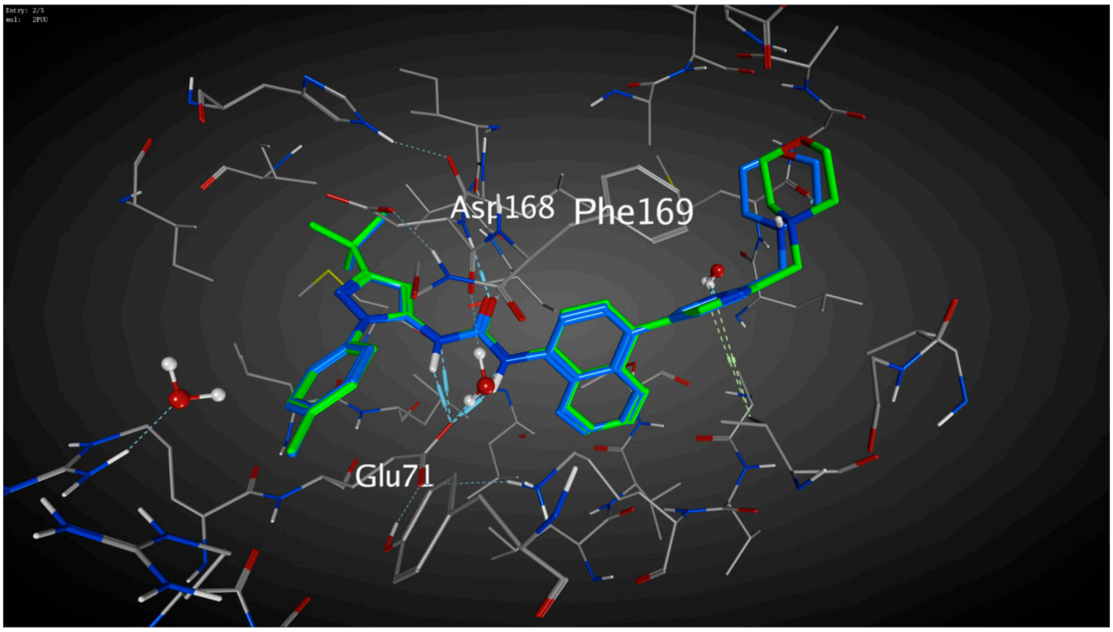


**Figure S1.** Comparison of the BIRB-796 molecular docking conformation (green) with the actual conformation (blue).

## 1. Process for synthesis of 1a-1o

4-methoxybenzoyl azide (**1a**): To a acetone solution of methyl 4-hydroxybenzoate (1.2 g, 7.22 mmol) were added $K_2CO_3$ (2 g, 8.67 mmol, 1.2 equiv) and dimethyl sulfate (1.18 g, 9.39 mmol, 1.3 equiv) and refluxed for 2 hours. The solvent acetone was then removed by vacuum evaporation to give a white soild (methyl 4-methoxybenzoate) which was used directly in the next step without purification. To a solution of the white solid in dichloromethane and water (25 mL, DCM: $H_2O$ = 1: 4), hydrochloric acid (10%, 4 mL, 1.5 equiv) and sodium nitrite (1.25 g, 18.05 mmol, 2 equiv) were added slowly at 0°C. The resulting mixture was stirred for 15 mins at 0°C. The reaction mixture was then concentrated, dissolved in EtOAc (30 mL) and dried over anhydrous $MgSO_4$. The crude product was purified by flash column chromatography (20% ethyl acetate in petroleum ether) to give **1a** as a light yellow oil (0.54 g, 34 % yield). $^1$H NMR (600 MHz, DMSO-*d*$_6$) δ7.27-7.21 (2H, *J*=8.5 Hz, d), 6.84-6.72 (2H, *J*=8.5 Hz, d), 3.97-3.46 (3H, *J*=7.4 Hz, t).

General Procedure for the formation of **1b**-**1o**

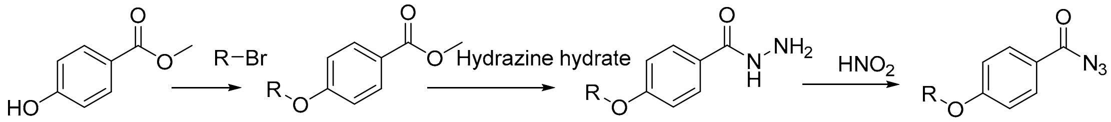


To a DMF solution of methyl 4-hydroxybenzoate were added NaH (60%, 1.2 equiv) and halohydrocarbon (1.3 equiv) slowly at 0°C. The resulting mixture was brought to 60°C and stirred for 2 hours. The solvent DMF was then removed by vacuum evaporation. The remaining mixture was added to a ethanol solution of hydrazine hydrate and reflux overnight. The solvent ethanol was removed by vacuum evaporation to give a white solid which was used directly in the next step without purification. To a mixed solution of the white solid in dichloromethane and water (25 mL, DCM: $H_2O$ = 1: 4), hydrochloric acid (10%, 1.5 equiv) and sodium nitrite (2 equiv) were added slowly at 0°C. The resulting mixture was stirred for 15 mins at 0°C. The reaction mixture was then

concentrated, dissolved in EtOAc (30 mL) and dried over anhydrous $MgSO_4$. The crude product was purified by flash column chromatography (20% ethyl acetate in petroleum ether) to give **1b**-**1o**.

4-ethoxybenzoyl azide (**1b**): Following the general procedure, bromoethane (1.40 g, 12.82 mmol, 1.3 equiv) hydrochloric acid (10%, 4 mL, 1.5 equiv) and sodium nitrite (1.15 g, 16.65 mmol, 2 equiv) gave **1b** as a light yellow oil (0.57 g, 36 % yield). $^1$H NMR (600 MHz, DMSO-$d_6$) δ7.25-7.20 (2H, *J*=8.5 Hz, m), 6.85-6.71 (2H, *J*=8.5 Hz, d), 3.51-3.46 (2H, *J*=4.9 Hz, d), 1.70 (3H, *J*=7.4 Hz, t).

4-propoxybenzoyl azide (**1c**): Following the general procedure, , 1-bromopropane (1.58 g, 12.82 mmol, 1.3 equiv), hydrochloric acid (10%, 4 mL, 1.5 equiv) and sodium nitrite (1.50 g, 15.45 mmol, 2 equiv)gave **1c** as a light yellow oil (0.63 g, 33 % yield). $^1$H NMR (600 MHz, DMSO-$d_6$): δ 7.20-7.14 (2H, d, *J* = 7.7 Hz ), 6.89-6.79 (2H, m), 3.94 (2H, t, *J* = 4.9 Hz ), 1.90-1.74 (2H, m) , 1.7 (t, *J*=7.4 Hz, 3H). 4-butoxybenzoyl azide (**1d**): Following the general procedure, 1-bromobutane (1.76 g, 12.82 mmol, 1.3 equiv), hydrochloric acid (10%, 4 mL, 1.5 equiv) and sodium nitrite (1 g, 14.41 mmol, 2 equiv)gave **1d** as a light yellow oil (0.61 g, 38 % yield). $^1$H NMR (600 MHz, DMSO-$d_6$): δ, 7.24-7.15 (2H, d, *J* = 7.7 Hz ), 6.84-6.79 (2H, m), 3.94 (2H, t, *J* = 4.9 Hz ), 1.90-1.74 (2H, m), 1.70-1,52 (2H, m), 1.44-1.32 (t, *J*=7.4 Hz, 3H). 4-(sec-butoxy)benzoyl azide (**1e**): Following the general procedure, 1-bromo-2-methylbutane (1.76 g, 12.82 mmol, 1.3 equiv), hydrochloric acid (10%, 4 mL, 1.5 equiv) and sodium nitrite (1 g, 14.41 mmol, 2 equiv)gave **1e** as a light yellow oil (0.52 g, 33 % yield). $^1$H NMR (600 MHz, DMSO-$d_6$): δ, 7.24-7.15 (2H, d, *J* = 8.5 Hz ), 6.84-6.79 (2H, d, *J* = 8.5 Hz), 4.04 (1H, d, *J* = 4.9 Hz ), 1.90-1.72 (3H, d, *J* = 6 Hz ) , 1.63-1.59 (2H, m), 1.44-1.32 (t, *J*=7.4 Hz, 3H). 4-(pentyloxy)benzoyl azide (**1f**): Following the general procedure, 1-bromopentane (1.94 g, 12.82 mmol, 1.3 equiv), hydrochloric acid (10%, 4 mL, 1.5 equiv) and sodium nitrite (0.93 g, 13.50 mmol, 2 equiv)gave **1f** as a light yellow soild (0.49 g, 30 % yield). $^1$H NMR (600 MHz, DMSO-$d_6$): δ 7.66-7.12 (2H, d, *J* = 8.5 Hz ), 6.99-6.83 (2H, d, *J* = 8.5 Hz), 3.95 (2H, t, *J* = 6.5 Hz ), 1.73-1.66 (d, 2 H), 1.35-1.27 (d, 4 H), 0.89 (t, *J*=7.2 Hz, 3 H). 4-(2-methoxyethoxy)benzoyl azide (**1g**): Following the general procedure, 1-bromo-2-methoxyethane (1.78 g, 12.82 mmol, 1.3 equiv), hydrochloric acid (10%, 4 mL, 1.5 equiv) and sodium nitrite (0.98 g, 14.27 mmol, 2 equiv)gave **1g** as a light yellow oil (0.56 g, 35 % yield). $^1$H NMR (600 MHz, DMSO-$d_6$): δ, 7.22-7.17 (d, *J*=8.5 Hz, 2 H), 6.81-6.73 (d, *J*=8.5 Hz, 2 H), 4.03 (2H, t, *J* = 6.5 Hz ), 3.70 (2H, t, *J* = 6.7 Hz ), 3.40 (3H, s, *J* = 6.9 Hz ).

4-isopropoxybenzoyl azide (**1H**): Following the general procedure, 2-bromopropane (1.58 g, 12.82 mmol, 1.3 equiv), hydrochloric acid (10%, 4 mL, 1.5 equiv) and sodium nitrite (1.07 g, 15.45 mmol, 2 equiv)gave **1H** as a light yellow oil (0.67 g, 45 % yield). $^1$H NMR (600 MHz, DMSO-$d_6$) δ7.33-7.17 (d, *J*=8.8 Hz, 2 H), 6.84-6.78 (d, *J*=9.0 Hz, 2 H), 3.84-3.68 (m, 1 H), 1.22 (d, *J*=6.0 Hz, 6 H).

3-chlorobenzoyl azide (**1i**): Following the general procedure, methyl 3-chlorobenzoate (1.5 g, 8.79 mmol, 1 equiv) hydrochloric acid (10%, 4 mL, 1.5 equiv) and sodium nitrite (1.21 g, 17.59 mmol, 2 equiv) gave **1i** as a light yellow oil (1.07 g, 66 % yield). $^1$H NMR (600 MHz, DMSO-$d_6$) δ 7.80 (d, *J*=9.0 Hz, 2 H), 7.64-7.56 (d, *J*=9.0 Hz, 2 H). 2-chlorobenzoyl azide (**1j**): Following the general procedure, methyl 2-chlorobenzoate (1.5 g, 8.79 mmol, 1 equiv), hydrochloric acid (10%, 4 mL, 1.5 equiv) and sodium nitrite (1.21 g, 17.59 mmol, 2 equiv) gave **1j** as a light yellow oil (1.09 g, 68 % yield). $^1$H NMR (600 MHz, DMSO-$d_6$) δ 7.98-7.76 (m, 2H), 7.56 -7.33 (m, 2H).

4-bromobenzoyl azide (**1k**): Following the general procedure, methyl 3-bromobenzoate (1.5 g, 6.98 mmol, 1 equiv), hydrochloric acid (10%, 4 mL, 1.5 equiv) and sodium nitrite (0.96 g, 13.95 mmol, 2 equiv) gave **1k** as a light yellow soild (0.93 g, 59 % yield). M.p. 46-47℃. $^{1}$H NMR (600 MHz, DMSO-$d_6$) δ 7.98 (s, 1H), 7.76 (d, $J$=9.0 Hz, 1H), 7.56 (d, $J$=9.0 Hz, 1H), 7.33 (m, 1H). 4-fluorobenzoyl azide (**1l**): Following the general procedure, methyl 3-fluorobenzoate (1.5 g, 9.73 mmol, 1 equiv), hydrochloric acid (10%, 4 mL, 1.5 equiv) and sodium nitrite (1.34 g, 19.49 mmol, 2 equiv) gave **1l** as a light yellow soild (1 g, 61 % yield). M.p. 39-41℃. $^{1}$H NMR (600 MHz, DMSO-$d_6$) δ 7.78 (s, 1H), 7.64 (d, $J$=9.0 Hz, 1H), 7.56 (d, $J$=9.0 Hz, 1H), 7.24 (m, 1H). 2-fluorobenzoyl azide (**1m**): Following the general procedure, methyl 2-fluorobenzoate (1.5 g, 9.73 mmol, 1 equiv), hydrochloric acid (10%, 4 mL, 1.5 equiv) and sodium nitrite (1.34 g, 19.49 mmol, 2 equiv) gave **1m** as a light yellow oil (0.87 g, 54 % yield). M.p. 32-34℃. $^{1}$H NMR (600 MHz, DMSO-$d_6$) δ 7.84-7.81 (d, $J$=8.5 Hz, 1 H), 7.71-7.61 (d, $J$=8.5 Hz, 1 H), 7.42 (d, $J$=8.5 Hz, 2 H).

4-methylbenzoyl azide (**1n**): Following the general procedure, methyl 3-methylbenzoate (1.5 g, 9.99 mmol, 1 equiv), hydrochloric acid (10%, 4 mL, 1.5 equiv) and sodium nitrite (1.38 g, 19.98 mmol, 2 equiv) gave **1n** as a light yellow oil (0.93 g, 57 % yield). M.p. 33-34℃. $^{1}$H NMR (600 MHz, DMSO-$d_6$) δ 7.79 (s, 1H), 7.72 (d, $J$=8.5 Hz, 1H), 7.31 (d, $J$=8.5 Hz, 1H), 3.46 (s, 3H), 2.24 (s, 3 H). 3-(3-morpholinopropyl)benzoyl azide (**1o**): Following the general procedure, T 4-(3-bromopropyl)morpholine (2.67 g, 12.82 mmol, 1.3 equiv), hydrochloric acid (10%, 4 mL, 1.5 equiv) and sodium nitrite (0.74 g, 10.74 mmol, 2 equiv)gave **1o** as a light yellow soild (0.44 g, 28 % yield). M.p. 49-51℃. $^{1}$H NMR (600 MHz, DMSO-$d_6$) δ7.85-7.80 (d, $J$=8.5 Hz, 2 H), 7.35-7.21 (d, $J$=8.5 Hz, 2 H), 4.48-4.32 (t, $J$=4.9 Hz, 1 H), 3.35-3.26 (t, $J$=5.7 Hz, 4 H), 3.04-2.87 (t, $J$=5.7 Hz, 4 H), 2.85 (m, 2 H), 2.39 (m, 2 H).

**2. General Procedure for the formation of 4a-4o**

To a DMF solution of **3a**-**3o** was added EDCI (1.1 equiv) and HOBt (1.1 equiv) at 0°C. The resulting mixture was brought to room temperature and stirred for 30 mins. Then (R) -2-amino-N-(3,5-dibromo-4-hydroxyphenethyl) -3- (3,5-dibromo-4-methoxyphenyl) propanamide (**2q**, 0.9 equiv) was added to the mixture and continue stirred for 1 hour. The reaction mixture was quenched with water and filtered. The crude product was recrystallized with ethanol (18 mL) to give **4a-4o**.

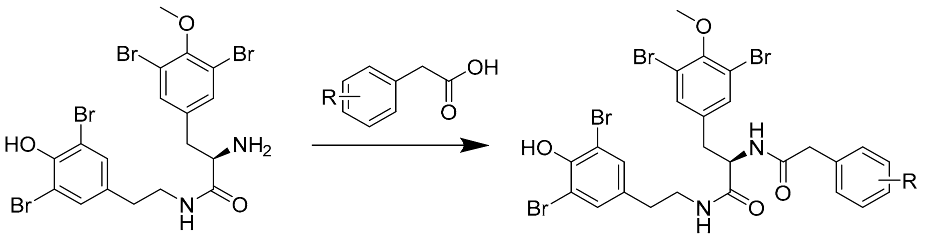

| Compound No. | R | Compound No. | R |
|---|---|---|---|
| 4A | 4-methyl ether | 4H | 4-isopropoxy ether |
| 4B | 4-ethyl ether | 4I | 3-Cl |
| 4C | 4-propyl ether | 4J | 2-Cl |
| 4D | 4-butyl ether | 4K | 4-Br |
| 4E | 4-sec-butyl ether | 4L | 4-F |

| | | | |
|---|---|---|---|
| 4F | 4-pentyl ether | 4M | 2-F |
| 4G | 4-(2-methoxyethoxy ether | 4N | 4-methy |
| | | 4O | 4-(3-morpholinopropoxy ether |

***(3,5-dibromo-4-methoxyphenyl) -2- [2- (4-methoxy) Phenyl) aminoacetyl] propionamide (4a)***

Following the general procedure, **3a** (0.25 g, 1.20 mmol)gave **4a** as a white solid (0.45 g, 46% yield). m.p.:151-153°C. $[\alpha]_D^{20}$-8.25 (c 0.66, MeOH). $^1$H NMR (600 MHz, DMSO-$d_6$) δ 8.44 (s, 1 H), 8.19 (br. s., 1 H), 7.45 (s, 2 H), 7.39 (s, 2 H), 7.20 - 7.25 (d, *J*=8.5 Hz, 2 H), 6.71 - 6.85 (d, *J*=8.5 Hz, 2 H), 6.28 (d, *J*=8.5 Hz, 1 H), 4.32 - 4.48 (m, 1 H), 3.75 (s, 3 H), 3.68 (s, 3 H), 3.26 - 3.35 (m, 1 H), 3.20 (dd, *J*=12.1, 5.7 Hz, 1 H), 2.85 (dd, *J*=13.5, 4.0 Hz, 1 H), 2.69 - 2.78 (m, 1 H), 2.60 (d, *J*=4.9 Hz, 2 H).

$^{13}$C NMR (150 MHz, DMSO-$d_6$) δ 171.0 (C=O), 169.0 (C=O), 152.2 (=C-O), 149.3(C-O), 138.0 (C-N), 132.8 (C-Br), 131.9 (C-Br), 129.3 (-C=), 127.2 (-C=), 117.3 (-C=), 115.1 (-C=), 112.2 (-C=), 70.2 (-C=), 60.7 (-O-CH3), 54.2 (CH2), 40.3 (CH2), 39.5 (CH2), 37.0 (CH2), 33.7 (CH2).

***(R) -N- (3,5-dibromo-4-hydroxybenzene) -3- (3,5-dibromo-4-methoxyphenyl) -2- [2- (4-ethoxy Phenyl) aminoacetyl] propionamide (4b)***

Following the general procedure, **3b** (0.25 g, 1.39 mmol) gave **4b** as a white solid (0.58 g, 53% yield). mp: 177-180°C. $[\alpha]_D^{20}$-8.92 (c 0.66, MeOH).

$^1$H NMR (600 MHz, DMSO-$d_6$) δ 8.44 (s, 1 H), 8.19 (br. s., 1 H), 7.45 (s, 2 H), 7.39 (s, 2 H), 7.20 - 7.25 (d, *J*=8.5 Hz, 2 H), 6.71 - 6.85 (d, *J*=8.5 Hz, 2 H), 6.28 (d, *J*=8.5 Hz, 1 H), 4.32 - 4.48 (d, 1 H), 3.75 (s, 3 H), 3.68 (s, 3 H), 3.26 - 3.35 (m, 1 H), 3.20 (dd, *J*=12.1, 5.7 Hz, 1 H), 2.85 (dd, *J*=13.5, 4.0 Hz, 1 H), 2.69 - 2.78 (m, 1 H), 2.60 (d, *J*=4.9 Hz, 2 H).

$^{13}$C NMR (150 MHz, DMSO-$d_6$) δ 170.0 (C=O), 168.6 (C=O), 155.8 (=C-O), 153.5 (=C-O), 150.3 (C-O), 137.0 (C-N), 133.9 (C-N), 132.6 (C-Br), 129.2 (C-Br), 126.7 (-C=), 119.8 (-C=), 114.8 (-C=), 112.5 (-C=), 70.42 (-C=), 63.4 (-O-CH3), 45.5 (CH2), 40.3 (CH2), 39.5 (CH2), 34.5 (CH2), 32.0 (CH2), 15.1 (CH3).

***(R) -N- (3,5-dibromo-4-hydroxybenzene) -3- (3,5-dibromo-4-methoxyphenyl) -2- [2- (4-propoxy Phenyl) aminoacetyl)] propanamide (4c)***

Following the general procedure, **3c**gave **4c** as a white solid (0.55 g, 53% yield). mp: 171-173 ℃. $[\alpha]_D^{20}$-9.35 (c 0.66, MeOH).

$^1$H NMR (600 MHz, DMSO-$d_6$) δ 8.44 (s, 1 H), 8.19 (br. s., 1 H), 7.45 (s, 2 H), 7.39 (s, 2 H), 7.20 - 7.25 (d, *J*=8.5 Hz, 2 H), 6.71 - 6.85 (d, *J*=8.5 Hz, 2 H), 6.28 (d, *J*=8.5 Hz, 1 H), 4.32 - 4.48 (d, 1 H), 3.75 (s, 3 H), 3.68 (s, 3 H), 3.26 - 3.35 (m, 1 H), 3.20 (dd, *J*=12.1, 5.7 Hz, 1 H), 2.85 (dd, *J*=13.5, 4.0 Hz, 1 H), 2.69 - 2.78 (m, 1 H), 2.60 (d, *J*=4.9 Hz, 2 H).

$^{13}$C NMR (150 MHz, DMSO-$d_6$) δ 170.0 (C=O), 169.0 (C=O), 155.7 (=C-O), 153.7 (=C-O), 138.0 (C-N), 133.8 (C-N), 132.8 (C-Br), 130.2 (C-Br), 128.3 (-C=), 119.9 (-C=), 117.6 (-C=), 114.9 (-C=), 69.4 (-C=), 60.7 (-O-CH3), 40.4 (CH2), 34.7 (CH2), 22.5 (CH2), 10.8 (CH3).

***(R) -N- (3,5-dibromo-4-hydroxybenzene) -3- (3,5-dibromo-4-methoxyphenyl) -2- [2- (4-butoxy Phenyl) aminoacetyl] propionamide (4d)***

Following the general procedure, **3d** (0.25 g, 1.20 mmol)gave **4d** as a white solid (0.41 g, 42%

yield). mp: 191-194 ℃. $[\alpha]_D^{20}$-8.25 (c 10.98, MeOH).

$^1$H NMR (600 MHz, DMSO-$d_6$) δ 8.44 (s, 1 H), 8.19 (br. s., 1 H), 7.45 (s, 2 H), 7.39 (s, 2 H), 7.20 - 7.25 (d, *J*=8.5 Hz, 2 H), 6.71 - 6.85 (d, *J*=8.5 Hz, 2 H), 6.28 (d, *J*=8.5 Hz, 1 H), 4.32 - 4.48 (m, 1 H), 3.75 (s, 3 H), 3.68 (s, 3 H), 3.26 - 3.35 (m, 1 H), 3.20 (dd, *J*=12.1, 5.7 Hz, 1 H), 2.85 (dd, *J*=13.5, 4.0 Hz, 1 H), 2.69 - 2.78 (m, 1 H), 2.60 (d, *J*=4.9 Hz, 2 H).

$^{13}$C NMR (150 MHz, DMSO-$d_6$) δ 171.0 (C=O), 170.0 (C=O), 155.7 (=C-O), 153.7 (=C-O), 149.3 (C-O), 134.6 (C-N), 133.9 (C-N), 132.7 (C-Br), 130.1 (C-Br), 128.2(-C=), 119.8(-C=), 114.7(-C=), 112.3(-C=), 67.6 (-O-CH3),40.7 (CH2), 34.5 (CH2), 31.2 (CH2), 19.1 (CH2), 14.1 (CH3).

***(R) -N- (3,5-dibromo-4-hydroxybenzene) -3- (3,5-dibromo-4-methoxyphenyl) -2- [2- (4-sec- Phenyl) aminoacetyl] propionamide (4e)***

Following the general procedure, 3e (0.25 g, 1.20 mmol) gave 4D as a white solid (0.49 g, 50% yield). mp: 160-166 ℃. $[\alpha]_D^{20}$-6.39 (c 0.66, MeOH).

$^1$H NMR (600 MHz, DMSO-$d_6$) δ 8.44 (s, 1 H), 8.19 (br. s., 1 H), 7.45 (s, 2 H), 7.39 (s, 2 H), 7.20 - 7.25 (d, *J*=8.5 Hz, 2 H), 6.71 - 6.85 (d, *J*=8.5 Hz, 2 H), 6.28 (d, *J*=8.5 Hz, 1 H), 4.32 - 4.48 (m, 1 H), 3.75 (s, 3 H), 3.68 (s, 3 H), 3.26 - 3.35 (m, 1 H), 3.20 (dd, *J*=12.1, 5.7 Hz, 1 H), 2.85 (dd, *J*=13.5, 4.0 Hz, 1 H), 2.69 - 2.78 (m, 1 H), 2.60 (d, *J*=4.9 Hz, 2 H).

$^{13}$C NMR (150 MHz, DMSO-$d_6$) δ 171.1 (C=O), 170.6 (C=O), 156.6 (=C-O), 152.19 (=C-O), 149.4 (C-O), 138.0 (C-N), 134.2 (C-N), 133.8 (C-Br), 132.8 (C-Br), 130.2 (-C=), 128.3 (-C=), 117.3 (-C=), 115.7 (-C=), 112.2 (-C=), 74.4 (-C=), 60.7 (-O-CH3), 54.1 (CH2), 41.6 (CH2), 40.4 (CH2), 37.0 (CH2), 33.7 (CH2), 28.9 (CH2), 19.5 (CH3), 9.9 (CH3).

***(R) -N- (3,5-dibromo-4-hydroxybenzene) -3- (3,5-dibromo-4-methoxyphenyl) -2- [2- (4-pentyl Phenyl) aminoacetyl] propionamide (4F)***

Following the general procedure, 3f (0.25 g, 1.13 mmol) gave 4F as a white solid (0.51 g, 55% yield). mp: 174-178 ℃. $[\alpha]_D^{20}$-6.71 (c 0.66, MeOH).

$^1$H NMR (600 MHz, DMSO-$d_6$) δ 8.25 (d, *J*=8.7 Hz, 1 H), 8.09 (t, *J*=5.6 Hz, 1 H), 7.51 (s, 2 H), 7.35 - 7.41 (d, 2 H), 6.95 - 7.01 (d, *J*=8.5 Hz, 2 H), 6.72 - 6.78 (d, *J*=8.7 Hz, 2 H), 4.33 - 4.42 (m, 1 H), 3.89 (t, *J*=6.5 Hz, 2 H), 3.72 - 3.78 (m, 3 H), 3.30 - 3.38 (m, 2 H), 3.15 - 3.27 (m, 2 H), 2.81 (dd, *J*=13.6, 3.9 Hz, 1 H), 2.54 - 2.67 (m, 3 H), 1.68 (quin, *J*=6.8 Hz, 2 H), 1.29 - 1.41 (m, 4 H), 0.89 (t, *J*=7.1 Hz, 3 H).

$^{13}$C NMR (150 MHz, DMSO-$d_6$) δ 171.0 (C=O), 170.6 (C=O), 157.5 (=C-O), 152.1 (=C-O), 149.4 (C-O), 137.9 (C-N), 133.8 (C-N), 132.8 (C-Br), 130.2 (C-Br), 128.3 (-C=), 117.2 (-C=), 114.4 (-C=), 112.2 (-C=), 67.7 (-C=), 60.7 (-O-CH3), 54.0 (CH2), 41.6 (CH2), 40.3 (CH2), 39.5 (CH2), 37.0 (CH2), 33.7 (CH2), 28.8 (CH2), 28.1 (CH2), 22.3 (CH2), 14.3 (CH3).

***(R) -N- (3,5-dibromo-4-hydroxybenzene) -3- (3,5-dibromo-4-methoxyphenyl) -2- [2- (4-pentyl Phenyl) aminoacetyl] propanamide (4g)***

Following the general procedure, 3g (0.25 g, 1.19 mmol)gave 4g as a white solid (0.62 g, 61% yield). mp: 181-183 . $[\alpha]_D^{20}$-6.91 (c 0.66, MeOH).

$^1$H NMR (600 MHz, DMSO-$d_6$) δ 8.44 (s, 1 H), 8.19 (br. s., 1 H), 7.45 (s, 2 H), 7.39 (s, 2 H), 7.20 - 7.25 (d, *J*=8.5 Hz, 2 H), 6.71 - 6.85 (d, *J*=8.5 Hz, 2 H), 6.28 (d, *J*=8.5 Hz, 1 H), 4.32 - 4.48 (m, 1 H), 3.75 (s, 3 H), 3.68 (s, 3 H), 3.26 - 3.35 (m, 1 H), 3.20 (dd, *J*=12.1, 5.7 Hz, 1 H), 2.85 (dd, *J*=13.5, 4.0 Hz, 1 H), 2.69 - 2.78 (m, 1 H), 2.60 (d, *J*=4.9 Hz, 2 H).

$^{13}$C NMR (150 MHz, DMSO-$d_6$) δ 170.1 (C=O), 168.7 (C=O), 155.4 (=C-O), 153.5 (=C-O),

149.3(C-O), 134.6 (C-N), 132.7 (C-N), 128.25 (C-Br), 119.7 (C-Br), 114.9 (-C=), 112.3 (-C=), 70.4 (-C=), 66.0 (-C=), 54.5 (-O-CH3), 40.3 (CH2), 39.5 (CH2), 34.50 (CH2), 31.2 (CH2), 22.5 (CH2), 10.8 (CH2)

***(R) -N- (3,5-dibromo-4-hydroxybenzene) -3- (3,5-dibromo-4-methoxyphenyl) -2- [2- (4-isopropyl Phenyl) aminoacetyl] propionamide (4h)***

Following the general procedure, 3H (0.25 g, 1.29 mmol)gave 4h as a white solid (0.57 g, 55% yield). mp: 169-173 ℃. $[\alpha]_D^{20}$-8.75 (c 0.66, MeOH).

$^1$H NMR (600 MHz, DMSO-$d_6$) δ 8.44 (s, 1 H), 8.19 (br. s., 1 H), 7.45 (s, 2 H), 7.39 (s, 2 H), 7.20 - 7.25 (d, *J*=8.5 Hz, 2 H), 6.71 - 6.85 (d, *J*=8.5 Hz, 2 H), 6.28 (d, *J*=8.5 Hz, 1 H), 4.32 - 4.48 (m, 1 H), 3.75 (s, 3 H), 3.68 (s, 3 H), 3.26 - 3.35 (m, 1 H), 3.20 (dd, *J*=12.1, 5.7 Hz, 1 H), 2.85 (dd, *J*=13.5, 4.0 Hz, 1 H), 2.69 - 2.78 (m, 1 H), 2.60 (d, *J*=4.9 Hz, 2 H).

$^{13}$C NMR (150 MHz, DMSO-$d_6$) δ 171.2 (C=O), 170.0 (C=O), 155.7 (=C-O), 153.7 (=C-O), 137.0 (C-O), 133.8 (C-N), 133.4 (C-N), 127.0 (C-Br), 119.9 (C-Br), 117.6 (-C=), 114.9 (-C=), 69.4 (-C=), 54.2 (-O-CH3), 40.4 (CH2), 34.7 (CH2), 22.5 (CH3), 10.8 (CH3).

***(R) -N- (3,5-dibromo-4-hydroxybenzene) -3- (3,5-dibromo-4-methoxyphenyl) -2- [2- (3-chlorophenyl ) Aminoacetyl] propionamide (4i)***

Following the general procedure, 3i (0.25 g, 1.47 mmol) gave 4i as a white solid (0.55 g, 59% yield). m.p .: 208-213 ℃. $[\alpha]_D^{20}$-8.16 (c 0.66, MeOH).

$^1$H NMR (600 MHz, DMSO-$d_6$) δ 8.40 (d, *J*=8.5 Hz, 1 H), 8.13 (t, *J*=5.6 Hz, 1 H), 7.46 - 7.52 (d, 2 H), 7.38 (s, 2 H), 7.23 - 7.27 (d, 2 H), 7.19 (s, 1 H), 6.98 - 7.09 (d, 1 H), 4.37 - 4.43 (m, 1 H), 3.75 (s, 3 H), 3.42 - 3.48 (m, 1 H), 3.40 (s, 1 H), 3.31 - 3.37 (m, 2 H), 2.81 (dd, *J*=13.7, 4.1 Hz, 1 H), 2.55 - 2.68 (m, 3 H).

$^{13}$C NMR (150 MHz, DMSO-$d_6$) δ 172.2 (C=O), 170.4 (C=O), 155.6 (=C-O), 152.6 (=C-O), 149.4 (C-O), 140.98 (C-N), 138.08 (C-N), 132.2 (C-Cl), 128.8 (C-Br), 122.0 (C-Br), 118.6 (-C=), 115.2 (-C=), 113.2 (-C=), 70.7 (-C=), 54.5(-O-CH3), 40.3 (CH2), 39.5 (CH2), 34.6 (CH2), 21.6 (CH2).

***(R) -N- (3,5-dibromo-4-hydroxybenzene) -3- (3,5-dibromo-4-methoxyphenyl) -2- [2- (2-chlorophenyl ) Aminoacetyl] propionamide (4J)***

Following the general procedure, 3j (0.25 g, 1.47 mmol) gave 4j as a white solid (0.49 g, 52% yield). m.p .: 186-193 ℃. $[\alpha]_D^{20}$-8.67 (c 0.66, MeOH).

$^1$H NMR (600 MHz, DMSO-$d_6$) δ 9.68 (br. s., 1 H), 8.35 (d, *J*=8.7 Hz, 1 H), 8.11 (t, *J*=5.6 Hz, 1 H), 7.54 (s, 2 H), 7.40 (s, 2 H), 7.35 (dd, *J*=7.7, 1.5 Hz, 1 H), 7.17 - 7.24 (d, 2 H), 7.11 - 7.15 (d, 1 H), 4.44 (ddd, *J*=10.5, 8.8, 4.0 Hz, 1 H), 3.72 - 3.79 (m, 3 H), 3.49 - 3.60 (m, 2 H), 3.31 - 3.41 (m, 2 H), 2.63 - 2.68 (m, 2 H), 2.57 - 2.63 (m, 2 H).

$^{13}$C NMR (150 MHz, DMSO-$d_6$) δ 171.0 (C=O), 169.0 (C=O), 152.2 (=C-O), 149.4 (=C-O), 138.0 (C-O), 134.4 (C-N), 134.3 (C-N), 133.9 (C-Cl), 133.9 (C-Br), 132.8 (C-Br), 132.0 (-C=), 129.3 (-C=), 128.7 (-C=), 127.2 (-C=), 117.3 (-C=), 112.2 (-C=), 60.7 (-O-CH3), 54.2, 40.3 (CH2), 39.5 (CH2), 37.0 (CH2), 33.7 (CH2).

***(R) -N- (3,5-dibromo-4-hydroxybenzene) -3- (3,5-dibromo-4-methoxyphenyl) -2- [2- (4-bromophenyl ) Aminoacetyl] propionamide (4k).***

Following the general procedure, 3k (0.25 g, 1.16 mmol) gave 4k as a white solid (0.49 g, 51% yield). m.p .: 221-225 ℃. $[\alpha]_D^{20}$-10.13 (c 0.66, MeOH).

$^{1}$H NMR (600 MHz, DMSO-$d_6$) δ 9.68 (br. s., 1 H), 8.37 (d, $J$=8.7 Hz, 1 H), 8.12 (t, $J$=5.6 Hz, 1 H), 7.49 (s, 2 H), 7.37 - 7.43 (d, 4 H), 7.05 (d, $J$=8.3 Hz, 2 H), 4.33 - 4.46 (m, 1 H), 3.75 (s, 3 H), 3.42 (d, $J$=13.9 Hz, 1 H), 3.29 - 3.38 (m, 2 H), 3.19 (dq, $J$=12.6, 6.3 Hz, 1 H), 2.81 (dd, $J$=13.6, 4.0 Hz, 1 H), 2.55 - 2.68 (m, 3 H).

$^{13}$C NMR (150 MHz, DMSO-$d_6$) δ 171.7 (C=O), 155.8 (C=O), 153.7 (=C-O), 149.4 (=C-O), 133.9 (C-O), 132.6 (C-N), 130.1 (C-N), 128.0 (C-Br), 119.8 (C-Br), 114.9 (-C=), 112.5 (-C=), 67.9 (-C=), 60.7 (-O-CH3), 40.3 (CH2), 39.5 (CH2).

***(R) -N- (3,5-dibromo-4-hydroxybenzene) -3- (3,5-dibromo-4-methoxyphenyl) -2- [2- (4-fluorophenyl ) Aminoacetyl] propionamide (4l)***

Following the general procedure, 3l (0.25 g, 1.62 mmol) gave 4l as a white solid (0.64 g, 52% yield). m.p .: 186-189 ℃. $[\alpha]_D^{20}$-7.72 (c 0.66, MeOH).

$^{1}$H NMR (600 MHz, DMSO-$d_6$) δ 8.44 (s, 1 H), 8.19 (br. s., 1 H), 7.45 (s, 2 H), 7.39 (s, 2 H), 7.20 - 7.25 (d, $J$=8.5 Hz, 2 H), 6.71 - 6.85 (d, $J$=8.5 Hz, 2 H), 6.28 (d, $J$=8.5 Hz, 1 H), 4.32 - 4.48 (m, 1 H), 3.75 (s, 3 H), 3.68 (s, 3 H), 3.26 - 3.35 (m, 1 H), 3.20 (dd, $J$=12.1, 5.7 Hz, 1 H), 2.85 (dd, $J$=13.5, 4.0 Hz, 1 H), 2.69 - 2.78 (m, 1 H), 2.60 (d, $J$=4.9 Hz, 2 H).

$^{13}$C NMR (150 MHz, DMSO-$d_6$) δ 171.0 (C=O), 170.2 (C=O), 162.0 (=C-O), 160.4 (=C-O), 152.2 (C-O), 149.3 (C-N), 137.9 (C-N), 134.3 (C-F), 133.8 (C-Br), 132.8 (C-Br), 131.0 (-C=), 130.9 (-C=), 117.3 (-C=), 115.2 (-C=), 115.0 (-C=), 112.2 (-C=), 60.7(-O-CH3), 54.0 (CH2), 41.5 (CH2), 37.0 (CH2), 33.6 (CH2).

***(R) -N- (3,5-dibromo-4-hydroxybenzene) -3- (3,5-dibromo-4-methoxyphenyl) -2- [2- (2-fluorophenyl ) Aminoacetyl] propionamide (4m)***

Following the general procedure, 3m (0.25 g, 1.62 mmol) gave 4m as a white solid (0.69 g, 56% yield). m.p .: 181-183 ℃. $[\alpha]_D^{20}$-8.05 (c 0.66, MeOH).

$^{1}$H NMR (600 MHz, DMSO-$d_6$) δ (s, 1 H), 8.19 (br. s., 1 H), 7.45 (s, 2 H), 7.39 (s, 2 H), 7.20 - 7.25 (d, $J$=8.5 Hz, 2 H), 6.71 - 6.85 (d, $J$=8.5 Hz, 2 H), 6.28 (d, $J$=8.5 Hz, 1 H), 4.32 - 4.48 (m, 1 H), 3.75 (s, 3 H), 3.68 (s, 3 H), 3.26 - 3.35 (m, 1 H), 3.20 (dd, $J$=12.1, 5.7 Hz, 1 H), 2.85 (dd, $J$=13.5, 4.0 Hz, 1 H), 2.69 - 2.78 (m, 1 H), 2.60 (d, $J$=4.9 Hz, 2 H).

$^{13}$C NMR (150 MHz, DMSO-$d_6$) δ171.6 (C=O), 170.6 (C=O), 155.8 (=C-O), 153.7 (=C-O), 152.0 (C-O), 139.8 (C-N), 133.8 (C-N), 133.4 (C-F), 130.3 (C-Br), 128.3 (C-Br), 119.9 (-C=), 117.6 (-C=), 112.9 (-C=), 67.9 (-C=), 60.7 (-O-CH3), 40.3 (CH2), 39.5 (CH2).

***(R) -N- (3,5-dibromo-4-hydroxybenzene) -3- (3,5-dibromo-4-methoxyphenyl) -2- [2- (4-methylbenzene Yl) aminoacetyl] propionamide (4n)***

Following the general procedure, 3n (0.25 g, 1.62 mmol) gave 4n as a white solid (0.69 g, 56% yield). m.p .: 230-233 ℃. $[\alpha]_D^{20}$-9.96 (c 0.66, MeOH).

$^{1}$H NMR (600 MHz, DMSO-$d_6$) δ 9.64 (br. s., 1 H), 8.27 (d, $J$=8.7 Hz, 1 H), 8.09 (t, $J$=5.6 Hz, 1 H), 7.51 (s, 2 H), 7.39 (s, 2 H), 7.00 - 7.04 (d, $J$=7.9 Hz, 2 H), 6.94 - 6.98 (d, $J$=7.9 Hz, 2 H), 4.32 - 4.45 (m, 1 H), 3.76 (s, 3 H), 3.39 (d, $J$=13.9 Hz, 1 H), 3.34 (dd, $J$=13.5, 6.7 Hz, 1 H), 3.27 (d, $J$=13.9 Hz, 1 H), 3.13 - 3.23 (m, 1 H), 2.80 (dd, $J$=13.6, 4.0 Hz, 1 H), 2.54 - 2.67 (m, 3 H), 2.19 - 2.25 (m, 3 H).

$^{13}$C NMR (150 MHz, DMSO-$d_6$) δ 171.2 (C=O), 170.6 (C=O), 155.6 (=C-O), 140.9 (=C-O), 138.0 (C-O), 132.2 (C-N), 128.8 (C-N), 122.0 (C-Br), 118.6 (C-Br), 115.2 (-C=), 113.2 (-C=), 67.9 (-C=),

60.4 (-O-CH3), 54.8 (CH2), 40.3 (CH2), 39.5 (CH2), 34.7 (CH2), 22.3 (CH3).

***(R) -N- (3,5-dibromo-4-hydroxybenzene) -3- (3,5-dibromo-4-methoxyphenyl) -2- [2- (4- (2- Methyl ethyl ether)) aminoacetyl] propionamide (4o)***

Following the general procedure, 3o (0.25 g, 0.90 mmol) gave 4o as a white solid (0.44 g, 45% yield). m.p .: 232-235 ℃. $[\alpha]_D^{20}$-8.59 (c 0.66, MeOH).

$^1$H NMR (600 MHz, DMSO-$d_6$) δ 8.44 (s, 1 H), 8.19 (br. s., 1 H), 7.45 (s, 2 H), 7.39 (s, 2 H), 7.20 - 7.25 (d, *J*=8.5 Hz, 2 H), 6.71 - 6.85 (d, *J*=8.5 Hz, 2 H), 6.28 (d, *J*=8.5 Hz, 1 H), 4.32 - 4.48 (m, 1 H), 3.75 (s, 3 H), 3.68 (s, 3 H), 3.26 - 3.35 (m, 1 H), 3.20 (dd, *J*=12.1, 5.7 Hz, 1 H), 2.85 (dd, *J*=13.5, 4.0 Hz, 1 H), 2.69 - 2.78 (m, 1 H), 2.60 (d, *J*=4.9 Hz, 2 H).

$^{13}$C NMR (150 MHz, DMSO-$d_6$) δ 170.0 (C=O), 169.0 (C=O), 155.7 (=C-O), 153.7 (=C-O), 137.9 (C-O), 133.8 (C-O), 132.8 (C-N), 130.2 (C-N), 128.3 (C-Br), 119.9 (C-Br), 117.6 (-C=), 114.9 (-C=), 74.6 (-C=), 69.4 (-C=), 60.7 (-O-CH3), 57.4 (CH2), 34.7 (CH2), 22.5 (CH2), 10.8 (CH3).

**3. General Procedure for the formation of 5a-5o**

Aryl azide **1a-1o** was added into 1,2-dichloroethane and stirred for 30 mins at 80°C. To the solution of 1a-1o in 1,2-dichloroethane, (R)-2-amino-N-(3,5-dibromo-4-hydroxy-phenethyl)-3-(3,5-dibromo-4-methoxyphenyl)propanamide (**2q**, 0.65 g , 1.03 mmol, 0.9 equiv) was added and stirred for 2 hours at 80°C. The reaction mixture was then concentrated and recrystallized with DCM (20 mL) to give **5a-5o**.

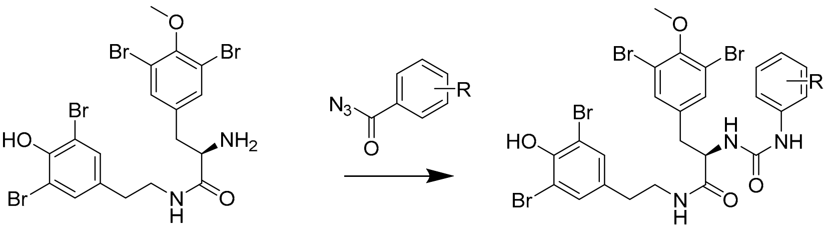


| Compound No. | R | 5H | 4-isopropoxy ether |
|---|---|---|---|
| 5A | 4-methyl ether | 5I | 3-Cl |
| 5B | 4-ethyl ether | 5J | 2-Cl |
| 5C | 4-propyl ether | 5K | 4-Br |
| 5D | 4-butyl ether | 5L | 4-F |
| 5E | 4-sec-butyl ether | 5M | 2-F |
| 5F | 4-pentyl ether | 5N | 4-methy |
| 5G | 4-(2-methoxyethoxy ether | 5O | 4-(3-morpholinopro poxy ether |

***(3,5-dibromo-4-methoxyphenyl) -2- [2- (4-methoxy) Phenyl) ureido] propionamide (5A):***

Following the general procedure, **1a** (0.25 g, 1.14 mmol) gave **5a** as a white solid (0.19 g, 21% yield). M.p.: 149-150°C. $[\alpha]_D^{20}$-14.31 (c 0.66, MeOH).

$^1$H NMR (600 MHz, DMSO-$d_6$) δ 8.44 (s, 1 H), 8.19 (br. s., 1 H), 7.45 (s, 2 H), 7.39 (s, 2 H), 7.20 - 7.25 (d, *J*=8.5 Hz, 2 H), 6.71 - 6.85 (d, *J*=8.5 Hz, 2 H), 6.28 (d, *J*=8.5 Hz, 1 H), 4.32 - 4.48 (m, 1 H), 3.75 (s, 3 H), 3.68 (s, 3 H), 3.26 - 3.35 (m, 1 H), 3.20 (dd, *J*=12.1, 5.7 Hz, 1 H), 2.85 (dd, *J*=13.5, 4.0 Hz, 1 H), 2.69 - 2.78 (m, 1 H), 2.60 (d, *J*=4.9 Hz, 2 H).

$^{13}$C NMR (150 MHz, DMSO-$d_6$) δ 171.5 (C=O), 155.1 (C=O), 153.8 (=C-O), 152.2 (=C-O), 137.8

(C-O), 133.9 (C-O), 132.7 (C-O), 119.8 (C-N), 117.3 (C-N), 114.9 (C-Br), 112.4 (C-Br), 67.9 (-O-CH3), 54.25 (CH2), 40.35 (CH2), 39.55 (CH2).

***(R) -N- (3,5-dibromo-4-hydroxybenzene) -3- (3,5-dibromo-4-methoxyphenyl) -2- [2- (4-ethoxy Phenyl) ureido] propionamide (5b)***

Following the general procedure, 1b (0.25 g, 1.31 mmol)gave 5b as a white solid (0.31 g, 29% yield). mp: 194-203 ℃. $[\alpha]_D^{20}$-19.14 (c 0.66, MeOH).

$^1$H NMR (600 MHz, DMSO-$d_6$) δ 8.42 (s, 1 H), 8.18 (t, *J*=5.6 Hz, 1 H), 7.44 (s, 2 H), 7.40 (s, 2 H), 7.15 - 7.23 (d, 2 H), 6.74 - 6.81 (d, 2 H), 6.26 (d, *J*=8.5 Hz, 1 H), 4.38 (td, *J*=8.5, 5.1 Hz, 1 H), 3.93 (q, *J*=7.0 Hz, 2 H), 3.73 - 3.76 (m, 3 H), 3.32 (dq, *J*=13.1, 6.8 Hz, 3 H), 2.84 (dd, *J*=13.7, 4.9 Hz, 1 H), 2.52 - 2.66 (m, 3 H), 1.28 (t, *J*=7.0 Hz, 3 H).

$^{13}$C NMR (150 MHz, DMSO-$d_6$) δ 158.3 (C=O), 154.7 (C=O), 152.6 (=C-O), 149.8 (=C-O), 143.0 (=C-O), 136.1 (C-O), 130.3 (C-O), 129.8 (C-N), 129.6 (C-N), 123.1 (C-N), 122.6 (C-Br), 120.9 (C-Br), 117.7 (-C=), 115.8 (-C=), 111.4 (-C=), 67.8 (-O-CH3), 41.6 (CH2), 40.3 (CH2), 39.5 (CH2), 29.2 (CH3).

***(R) -N- (3,5-dibromo-4-hydroxybenzene) -3- (3,5-dibromo-4-methoxyphenyl) -2- [2- (4-propoxy Phenyl) ureido] propionamide (5c)***

Following the general procedure, 1c (0.25 g, 1.22 mmol)gave 5c as a white solid (0.33 g, 34% yield). mp: 173-176 ℃. $[\alpha]_D^{20}$-15.38 (c 0.66, MeOH).

$^1$H NMR (600 MHz, DMSO-$d_6$) δ 8.44 (s, 1 H), 8.19 (br. s., 1 H), 7.45 (s, 2 H), 7.39 (s, 2 H), 7.20 - 7.25 (d, *J*=8.5 Hz, 2 H), 6.71 - 6.85 (d, *J*=8.5 Hz, 2 H), 6.28 (d, *J*=8.5 Hz, 1 H), 4.32 - 4.48 (m, 1 H), 3.75 (s, 3 H), 3.68 (s, 3 H), 3.26 - 3.35 (m, 1 H), 3.20 (dd, *J*=12.1, 5.7 Hz, 1 H), 2.85 (dd, *J*=13.5, 4.0 Hz, 1 H), 2.69 - 2.78 (m, 1 H), 2.60 (d, *J*=4.9 Hz, 2 H).

$^{13}$C NMR (150 MHz, DMSO-$d_6$) δ 172.3 (C=O), 155.2 (C=O), 153.7 (=C-O), 152.2 (=C-O), 138.2 (C-O), 133.9 (C-N), 132.3 (C-N), 119.8 (C-N), 117.3 (C-Br), 114.9 (C-Br), 113.0 (-C=), 67.9 (-O-CH3), 54.1 (CH2), 40.3 (CH2), 39.5 (CH2), 34.5 (CH2), 22.5 (CH2), 10.8 (CH3).

***(R) -N- (3,5-dibromo-4-hydroxybenzene) -3- (3,5-dibromo-4-methoxyphenyl) -2- [2- (4-butoxy Phenyl) ureido] propionamide (5d)***

Following the general procedure,  1d (0.25 g, 1.14 mmol)gave 5d as a white solid (0.22 g, 24% yield). mp: 185-188 ℃. $[\alpha]_D^{20}$-10.88 (c 0.66, MeOH).

$^1$H NMR (600 MHz, DMSO-$d_6$) δ 8.44 (s, 1 H), 8.19 (br. s., 1 H), 7.45 (s, 2 H), 7.39 (s, 2 H), 7.20 - 7.25 (d, *J*=8.5 Hz, 2 H), 6.71 - 6.85 (d, *J*=8.5 Hz, 2 H), 6.28 (d, *J*=8.5 Hz, 1 H), 4.32 - 4.48 (m, 1 H), 3.75 (s, 3 H), 3.68 (s, 3 H), 3.26 - 3.35 (m, 1 H), 3.20 (dd, *J*=12.1, 5.7 Hz, 1 H), 2.85 (dd, *J*=13.5, 4.0 Hz, 1 H), 2.69 - 2.78 (m, 1 H), 2.60 (d, *J*=4.9 Hz, 2 H).

$^{13}$C NMR (150 MHz, DMSO-$d_6$) δ 172.1 (C=O), 155.7 (C=O), 153.5 (=C-O), 149.3 (=C-O), 137.9 (C-O), 134.6 (C-N), 132.7 (C-N), 119.8 (C-N), 117.4 (C-Br), 114.9 (C-Br), 112.3 (-C=), 66.0 (-O-CH3), 55.1 (CH2), 40.3 (CH2), 39.5 (CH2), 34.5 (CH2), 31. (CH2)2, 22.5 (CH2), 10.8 (CH3).

***(R) -N- (3,5-dibromo-4-hydroxybenzene) -3- (3,5-dibromo-4-methoxyphenyl) -2- [2- (4-sec- Phenyl) ureido] propionamide (5E)***

Following the general procedure,  1e (0.25 g, 1.14 mmol)1egave 5e as a white solid (0.27g, 29% yield). mp: 174-176 ℃. $[\alpha]_D^{20}$-14.37 (c 0.66, MeOH).

$^1$H NMR (600 MHz, DMSO-$d_6$) δ 8.44 (s, 1 H), 8.19 (br. s., 1 H), 7.45 (s, 2 H), 7.39 (s, 2 H), 7.20

- 7.25 (d, *J*=8.5 Hz, 2 H), 6.71 - 6.85 (d, *J*=8.5 Hz, 2 H), 6.28 (d, *J*=8.5 Hz, 1 H), 4.32 - 4.48 (m, 1 H), 3.75 (s, 3 H), 3.68 (s, 3 H), 3.26 - 3.35 (m, 1 H), 3.20 (dd, *J*=12.1, 5.7 Hz, 1 H), 2.85 (dd, *J*=13.5, 4.0 Hz, 1 H), 2.69 - 2.78 (m, 1 H), 2.60 (d, *J*=4.9 Hz, 2 H).

$^{13}$C NMR (150 MHz, DMSO-$d_6$) δ 171.8 (C=O), 155.8 (C=O), 153.7 (=C-O), 151.8 (=C-O), 136.1 (C-O), 133.9 (C-N), 132.6 (C-N), 119.8 (C-N), 117.3 (C-Br), 114.9 (C-Br), 112.5 (-C=), 67.9 (-O-CH3), 60.7 (CH2), 40.3 (CH2), 39.5 (CH2), 34.5 (CH2), 28.9 (CH2), 28.1 (CH2), 22.3 (CH3), 14.3 (CH3).

***(R) -N- (3,5-dibromo-4-hydroxybenzene) -3- (3,5-dibromo-4-methoxyphenyl) -2- [2- (4-pentyl Phenyl) ureido] propionamide (5f)***

Following the general procedure, 1f (0.25 g, 1.07 mmol)1fgave 5f as a white solid (0.33 g, 34% yield). mp: 186-193 ℃. $[\alpha]_D^{20}$-14.89 (c 0.66, MeOH).

$^{1}$H NMR (600 MHz, DMSO-$d_6$) δ 8.44 (s, 1 H), 8.19 (br. s., 1 H), 7.45 (s, 2 H), 7.39 (s, 2 H), 7.20 - 7.25 (d, *J*=8.5 Hz, 2 H), 6.71 - 6.85 (d, *J*=8.5 Hz, 2 H), 6.28 (d, *J*=8.5 Hz, 1 H), 4.32 - 4.48 (m, 1 H), 3.75 (s, 3 H), 3.68 (s, 3 H), 3.26 - 3.35 (m, 1 H), 3.20 (dd, *J*=12.1, 5.7 Hz, 1 H), 2.85 (dd, *J*=13.5, 4.0 Hz, 1 H), 2.69 - 2.78 (m, 1 H), 2.60 (d, *J*=4.9 Hz, 2 H).

$^{13}$C NMR (150 MHz, DMSO-$d_6$) δ 171.5 (C=O), 155.1 (C=O), 153.8 (=C-O), 152.2 (=C-O), 137.8 (C-O), 133.9 (C-N), 132.7 (C-N), 119.8 (C-N), 117.3 (C-Br), 114.9 (C-Br), 112.4 (-C=), 68.0 (-O-CH3), 60.7 (CH2), 54.1 (CH2), 38.0 (CH2), 33.7 (CH2), 28.8, (CH2), 28.1 (CH2), 22.3 (CH2), 14.3 (CH3).

***(R) -N- (3,5-dibromo-4-hydroxybenzene) -3- (3,5-dibromo-4-methoxyphenyl) -2- [2- (4- (3-Morpholinopropyl) phenyl) ureido] propionamide (5g)***

Following the general procedure, 1g (0.25 g, 1.13 mmol)1ggave 5g as a white solid (0.37 g, 39% yield). mp: 179-182 ℃. $[\alpha]_D^{20}$-10.44 (c 0.66, MeOH).

$^{1}$H NMR (600 MHz, DMSO-$d_6$) δ 8.49 (s, 1 H), 8.18 (t, *J*=5.2 Hz, 1 H), 7.45 (s, 2 H), 7.29 (s, 2 H), 7.14 - 7.25 (d, *J*=8.8 Hz, 2 H), 6.65 - 6.84 (d, *J*=8.8 Hz, 2 H), 6.34 (d, *J*=8.5 Hz, 1 H), 4.27 - 4.45 (m, 1 H), 3.92 (t, *J*=6.2 Hz, 2 H), 3.75 (s, 3 H), 3.50 - 3.61 (m, 4 H), 3.29 (dq, *J*=13.2, 6.6 Hz, 1 H), 3.10 - 3.21 (m, 1 H), 2.85 (dd, *J*=13.6, 4.6 Hz, 1 H), 2.70 (dd, *J*=13.5, 8.6 Hz, 1 H), 2.54 (br. s., 2 H), 2.39 (t, *J*=7.2 Hz, 2 H), 2.35 (br. s., 4 H), 1.82 (quin, *J*=6.7 Hz, 2 H).

$^{13}$C NMR (150 MHz, DMSO-$d_6$) δ 171.5 (C=O), 155.2 (C=O), 153.7 (=C-O), 152.2 (=C-O), 137.8 (C-O), 133.86 (C-N), 132.3 (C-N), 119.8 (C-Br), 113.0 (C-Br), 66.6 (-O-CH3), 66.3 (CH2), 60.7 (CH2), 55.3 (CH2), 53.8 (CH2), 38.0 (CH2), 33.8 (CH2), 26.3 (CH2).

***(R) -N- (3,5-dibromo-4-hydroxybenzene) -3- (3,5-dibromo-4-methoxyphenyl) -2- [2- (4-isopropyl Phenyl) ureido] propionamide (5h)***

Following the general procedure, 1h (0.25 g, 1.22 mmol)gave 5h as a white solid (0.25 g, 26% yield). mp: 175-177 ℃. $[\alpha]_D^{20}$-10.91 (c 0.66, MeOH).

$^{1}$H NMR (600 MHz, DMSO-$d_6$) δ 8.44 (s, 1 H), 8.19 (br. s., 1 H), 7.45 (s, 2 H), 7.39 (s, 2 H), 7.20 - 7.25 (d, *J*=8.5 Hz, 2 H), 6.71 - 6.85 (d, *J*=8.5 Hz, 2 H), 6.28 (d, *J*=8.5 Hz, 1 H), 4.32 - 4.48 (m, 1 H), 3.75 (s, 3 H), 3.68 (s, 3 H), 3.26 - 3.35 (m, 1 H), 3.20 (dd, *J*=12.1, 5.7 Hz, 1 H), 2.85 (dd, *J*=13.5, 4.0 Hz, 1 H), 2.69 - 2.78 (m, 1 H), 2.60 (d, *J*=4.9 Hz, 2 H).

$^{13}$C NMR (150 MHz, DMSO-$d_6$) δ 169.1 (C=O), 155.7 (C=O), 153.7 (=C-O), 149.3 (=C-O), 134.6 (C-O), 133.9 (C-N), 132.7 (C-N), 119.8 (C-N), 117.2 (C-Br), 114.9 (C-Br), 112.3 (-C=), 69.4 (-O-CH3), 40.7 (CH2), 34.5 (CH2), 22.5 (CH2), 10.8 (CH3).

***(R) -N- (3,5-dibromo-4-hydroxybenzene) -3- (3,5-dibromo-4-methoxyphenyl) -2- [2- (4- (3-Chlorophenyl)) ureido] propionamide (5i)***

Following the general procedure, 1i (0.25 g, 1.38 mmol)1i gave 5i as a white solid (0.31 g, 29% yield). mp: 205-207 ℃. $[\alpha]_D^{20}$-10.75 (c 0.66, MeOH).

$^1$H NMR (600 MHz, DMSO-$d_6$) δ 8.44 (s, 1 H), 8.19 (br. s., 1 H), 7.45 (s, 2 H), 7.39 (s, 2 H), 7.20 - 7.25 (d, *J*=8.5 Hz, 2 H), 6.71 - 6.85 (d, *J*=8.5 Hz, 2 H), 6.28 (d, *J*=8.5 Hz, 1 H), 4.32 - 4.48 (m, 1 H), 3.75 (s, 3 H), 3.68 (s, 3 H), 3.26 - 3.35 (m, 1 H), 3.20 (dd, *J*=12.1, 5.7 Hz, 1 H), 2.85 (dd, *J*=13.5, 4.0 Hz, 1 H), 2.69 - 2.78 (m, 1 H), 2.60 (d, *J*=4.9 Hz, 2 H).

$^{13}$C NMR (150 MHz, DMSO-$d_6$) δ 172.3 (C=O), 155.2 (C=O), 153.7 (=C-O), 152.6 (=C-O), 138.2 (C-O), 133.9 (C-N), 132.3 (C-N), 119.7 (C-N), 117.9 (C-Br), 114.2 (C-Br), 112.9 (-C=), 60.7 (-O-CH3), 54.0 (CH2), 37.0 (CH2), 33.7 (CH2).

***(R) -N- (3,5-dibromo-4-hydroxybenzene) -3- (3,5-dibromo-4-methoxyphenyl) -2- [2- (4- (2-Chlorophenyl)) ureido] propionamide (5j)***

Following the general procedure, 1j (0.25 g, 1.38 mmol)1jgave 5j as a white solid (0.33 g, 31% yield). mp: 224-226 ℃. $[\alpha]_D^{20}$-20.89 (c 0.66, MeOH).

$^1$H NMR (600 MHz, DMSO-$d_6$) δ 8.44 (s, 1 H), 8.19 (br. s., 1 H), 7.45 (s, 2 H), 7.39 (s, 2 H), 7.20 - 7.25 (d, *J*=8.5 Hz, 2 H), 6.71 - 6.85 (d, *J*=8.5 Hz, 2 H), 6.28 (d, *J*=8.5 Hz, 1 H), 4.32 - 4.48 (m, 1 H), 3.75 (s, 3 H), 3.68 (s, 3 H), 3.26 - 3.35 (m, 1 H), 3.20 (dd, *J*=12.1, 5.7 Hz, 1 H), 2.85 (dd, *J*=13.5, 4.0 Hz, 1 H), 2.69 - 2.78 (m, 1 H), 2.60 (d, *J*=4.9 Hz, 2 H).

$^{13}$C NMR (150 MHz, DMSO-$d_6$) δ 171.2 (C=O), 155.7 (C=O), 153.7 (=C-O), 149.3 (=C-O), 132.7 (C-O), 130.0 (C-N), 119.8 (C-N), 117.2 (C-Br), 114.9 (C-Br), 112.3 (-C=), 60.7 (-O-CH3), 54.0 (CH2), 34.5 (CH2).

***(R) -N- (3,5-dibromo-4-hydroxybenzene) -3- (3,5-dibromo-4-methoxyphenyl) -2- [2- (4- (4-Bromophenyl)) ureido] propionamide (5k)***

Following the general procedure, 1k (0.25 g, 1.11 mmol)1kgave 5k as a white solid (0.31 g, 34% yield). mp: 217-219 ℃. $[\alpha]_D^{20}$-10.46 (c 0.66, MeOH).

$^1$H NMR (600 MHz, DMSO-$d_6$) δ 9.05 (br. s., 1 H), 8.21 (s, 1 H), 7.47 (s, 2 H), 7.31 - 7.38 (d, 4 H), 7.19 (s, 2 H), 6.68 (br. s., 1 H), 4.39 (d, *J*=4.9 Hz, 1 H), 3.74 (s, 3 H), 3.25 (s, 1 H), 3.11 (br. s., 1 H), 2.88 (d, *J*=4.7 Hz, 1 H), 2.71 - 2.78 (m, 1 H), 2.42 - 2.54 (m, 4 H)

$^{13}$C NMR (150 MHz, DMSO-$d_6$) δ 171.2 (C=O), 154.9 (C=O), 152.2 (=C-O), 140.1 (=C-O), 137.8 (C-O), 133.9 (C-N), 131.7 (C-N), 120.0 (C-N), 117.3 (C-Br), 114.0 (C-Br), 112.7 (-C=), 60.7 (-O-CH3), 54.3 (CH2), 41.1 (CH2), 37.9 (CH2), 33.9 (CH2).

***(R) -N- (3,5-dibromo-4-hydroxybenzene) -3- (3,5-dibromo-4-methoxyphenyl) -2- [2- (4- (4-Fluorophenyl)) ureido] propionamide (5l)***

Following the general procedure, 1l (0.25 g, 1.51 mmol)1lgave 5l as a white solid (0.44 g, 38% yield). mp: 180-183 ℃. $[\alpha]_D^{20}$-17.09 (c 0.66, MeOH).

$^1$H NMR (600 MHz, DMSO-$d_6$) δ 8.44 (s, 1 H), 8.19 (br. s., 1 H), 7.45 (s, 2 H), 7.39 (s, 2 H), 7.20 - 7.25 (d, *J*=8.5 Hz, 2 H), 6.71 - 6.85 (m, *J*=8.5 Hz, 2 H), 6.28 (d, *J*=8.5 Hz, 1 H), 4.32 - 4.48 (m, 1 H), 3.75 (s, 3 H), 3.68 (s, 3 H), 3.26 - 3.35 (m, 1 H), 3.20 (dd, *J*=12.1, 5.7 Hz, 1 H), 2.85 (dd, *J*=13.5, 4.0 Hz, 1 H), 2.69 - 2.78 (m, 1 H), 2.60 (d, *J*=4.9 Hz, 2 H).

$^{13}$C NMR (150 MHz, DMSO-$d_6$) δ 172.0 (C=O), 155.1 (C=O), 152.0 (=C-O), 139.6 (=C-O),

137.0 (C-O), 133.4 (C-F), 129.4 (C-N), 127.8 (C-N), 122.9 (C-N), 121.4 (C-Br), 117.6 (C-Br), 114.0 (-C=), 112.0 (-C=), 60.7 (-O-CH3), 54.0 (CH2), 34.5 (CH2).

***(3,5-dibromo-4-hydroxybenzene) -3- (3,5-dibromo-4-methoxyphenyl) -2- [2- (4- (2- Fluorophenyl)) ureido] propionamide 5m)***

Following the general procedure, 1m (0.25 g, 1.11 mmol)1mgave 5m as a white solid (0.35 g, 31% yield). mp: 177-180 ℃. $[\alpha]_D^{20}$-16.07 (c 0.66, MeOH).

$^1$H NMR (600 MHz, DMSO-$d_6$) δ 8.52 (br. s., 1 H), 8.22 (t, *J*=5.2 Hz, 1 H), 8.02 (td, *J*=8.3, 1.3 Hz, 1 H), 7.47 (s, 2 H), 7.23 (br. s., 2 H), 7.15 (ddd, *J*=11.5, 8.2, 1.1 Hz, 1 H), 6.98 - 7.07 (m, 2 H), 6.88 - 6.94 (m, 1 H), 4.40 (td, *J*=8.5, 5.1 Hz, 1 H), 3.75 (s, 3 H), 3.22 - 3.33 (m, 1 H), 3.14 (dd, *J*=12.3, 5.6 Hz, 1 H), 2.88 (dd, *J*=13.7, 4.9 Hz, 1 H), 2.70 (dd, *J*=13.7, 8.7 Hz, 1 H).

$^{13}$C NMR (150 MHz, DMSO-$d_6$) δ 171.0 (C=O), 152.2 (C=O), 149.4 (=C-O), 138.0 (=C-O), 134.3 (C-O), 133.6 (C-F), 132.8 (C-N), 131.9 (C-N), 129.3 (C-N), 127.2 (C-Br), 117.3 (C-Br), 112.2 (-C=), 60.7 (-O-CH3), 54.2 (CH2), 37.0 (CH2), 33.7 (CH2).

***(R) -N- (3,5-dibromo-4-hydroxybenzene) -3- (3,5-dibromo-4-methoxyphenyl) -2- [2- (4- (4- Methylphenyl)) ureido] propionamide (5n)***

Following the general procedure, 1n (0.25 g, 1.55 mmol)1ngave 5n as a white solid (0.43 g, 36% yield). mp: 208-213 ℃. $[\alpha]_D^{20}$-11.50 (c 0.66, MeOH).

$^1$H NMR (600 MHz, DMSO-$d_6$) δ 8.59 (s, 1 H), 8.19 (t, *J*=5.6 Hz, 1 H), 7.46 (s, 2 H), 7.18 - 7.27 (m, 4 H), 7.00 (d, *J*=8.3 Hz, 2 H), 6.41 (d, *J*=8.5 Hz, 1 H), 4.38 (td, *J*=8.5, 5.1 Hz, 1 H), 3.75 (s, 3 H), 3.27 (dd, *J*=13.4, 6.6 Hz, 1 H), 3.11 - 3.18 (m, 1 H), 2.86 (dd, *J*=13.7, 4.9 Hz, 1 H), 2.71 (dd, *J*=13.7, 8.5 Hz, 1 H), 2.20 (s, 3 H)

$^{13}$C NMR (150 MHz, DMSO-$d_6$) δ 171.1 (C=O), 155.6 (C=O), 152.3 (=C-O), 140.1 (=C-O), 137.8 (C-N), 133.9 (C-N), 131.7 (C-N), 120.0 (C-Br), 117.3 (C-Br), 112.5 (-C=), 60.7 (-O-CH3), 54.3 (CH2), 37.9 (CH2), 33.9 (CH3).

***(R) -N- (3,5-dibromo-4-hydroxybenzene) -3- (3,5-dibromo-4-methoxyphenyl) -2- [2- (4- (2- Methyl ethyl ether)) ureido] propionamide (5O)***

Following the general procedure, 1o (0.25 g, 0.86 mmol)1o gave 5o as a white solid (0.29 g, 38% yield). mp: 211-214 ℃. $[\alpha]_D^{20}$-13.35 (c 0.66, MeOH).

$^1$H NMR (600 MHz, DMSO-$d_6$) δ 8.44 (s, 1 H), 8.19 (br. s., 1 H), 7.45 (s, 2 H), 7.39 (s, 2 H), 7.20 - 7.25 (d, *J*=8.5 Hz, 2 H), 6.71 - 6.85 (d, *J*=8.5 Hz, 2 H), 6.28 (d, *J*=8.5 Hz, 1 H), 4.32 - 4.48 (m, 1 H), 3.75 (s, 3 H), 3.68 (s, 3 H), 3.26 - 3.35 (m, 1 H), 3.20 (dd, *J*=12.1, 5.7 Hz, 1 H), 2.85 (dd, *J*=13.5, 4.0 Hz, 1 H), 2.69 - 2.78 (m, 1 H), 2.60 (d, *J*=4.9 Hz, 2 H).

$^{13}$C NMR (150 MHz, DMSO-$d_6$) δ 171.0 (C=O), 162.1 (C=O), 160.4 (=C-O), 152.2 (=C-O), 149.3 (C-N), 137.9 (C-N), 134.3 (C-N), 133.8 (C-Br), 132.8 (C-Br), 130.9 (-C=), 117.2 (-C=), 115.2 (-C=), 112.2 (-C=), 60.7 (-O-CH3), 58.0 (-O-CH3), 54.5 (CH2), 37.0 (CH2), 33.7 (CH2).

**3. General Procedure for the formation of 6a-6o**

The aryl azide **1a**-**1o** was added into 1,2-dichloroethane and stirred for 30 mins at 80°C. To the solution of 1a in 1,2-dichloroethane, 2-(3,5-dibromo-4-methoxyphenyl)ethan-1-amine (**2p**, 0.51 g , 1.64 mmol, 0.9 equiv) was added and stirred for 2 hours at 80°C. The reaction mixture was then

concentrated and recrystallized with DCM (20 mL) to give **6a-6o**.

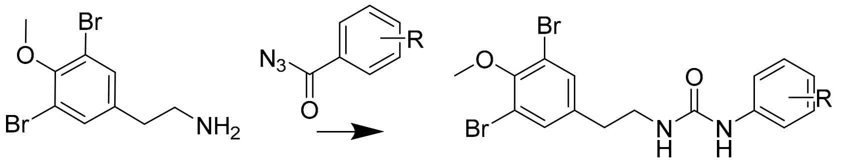


| Compound No. | R | 6H | 4-isopropoxy ether |
|---|---|---|---|
| 6A | 4-methyl ether | 6I | 3-Cl |
| 6B | 4-ethyl ether | 6J | 2-Cl |
| 6C | 4-propyl ether | 6K | 4-Br |
| 6D | 4-butyl ether | 6L | 4-F |
| 6E | 4-sec-butyl ether | 6M | 2-F |
| 6F | 4-pentyl ether | 6N | 4-methy |
| 6G | 4-(2-methoxyethoxy ether | 6O | 4-(3-morpholinopropoxy ether |

***1- (3,5-dibromo-4-methoxyphenyl) -3- (4-methoxyphenyl) urea (6a)***

Following the general procedure, **1a** (0.4 g, 1.82 mmol)gave **6a** as a white solid (0.44 g, 49% yield). M.p.: 151-155°C.

$^1$H NMR (600 MHz, DMSO-$d_6$) δ 8.28 (s, 1 H), 7.43 - 7.60 (m, 2 H), 7.17 - 7.35 (m, 2 H), 6.69 - 6.87 (m, 2 H), 6.06 (t, *J*=5.6 Hz, 1 H), 3.77 (s, 3 H), 3.69 (s, 3 H), 3.30 - 3.32 (m, 2 H), 2.71 ppm (t, *J*=6.9 Hz, 2 H)

$^{13}$C NMR (150 MHz, DMSO-$d_6$) δ 155.8 (C=O), 154.3 (=C-O), 133.9 (C-N), 133.4 (C-N), 119.8 (C-Br), 117.5 (C-Br), 114.2 (-C=), 60.7 (-O-CH3), 55.5 (CH2), 40.5 (CH2), 34.7 (CH2).

***1- (3,5-dibromo-4-methoxyphenyl) -3- (4-ethoxyphenyl) urea (6b)***

Following the general procedure, 1b (0.4 g, 2.09 mmol)gave 6b as a white solid (0.46 g, 47% yield). mp: 176-180 ° C.

$^1$H NMR (600 MHz, DMSO-$d_6$) δ 8.44 (s, 1 H), 8.19 (br. s., 1 H), 7.45 (s, 2 H), 7.39 (s, 2 H), 7.20 - 7.25 (d, *J*=8.5 Hz, 2 H), 6.71 - 6.85 (d, *J*=8.5 Hz, 2 H), 6.28 (d, *J*=8.5 Hz, 1 H), 4.32 - 4.48 (m, 1 H), 3.75 (s, 3 H), 3.68 (s, 3 H), 3.26 - 3.35 (m, 1 H), 3.20 (dd, *J*=12.1, 5.7 Hz, 1 H), 2.85 (dd, *J*=13.5, 4.0 Hz, 1 H), 2.69 - 2.78 (m, 1 H), 2.60 (d, *J*=4.9 Hz, 2 H).

$^{13}$C NMR (150 MHz, DMSO-$d_6$) δ 155.7 (C=O), 153.6 (=C-O), 152.0 (=C-O), 139.8 (C-N), 133.8 (C-N), 133.4 (C-Br), 114.8 (-C=), 63.4 (-O-CH3), 60.7 (-O-CH3), 40.5 (CH2), 34.7 (CH2), 15.1 (CH3).

***1- (3,5-dibromo-4-methoxyphenyl) -3- (4-propoxyphenyl) urea (6c)***

Following the general procedure, 1c (0.4 g, 1.95 mmol)gave 6c as a white solid (0.39 g, 41% yield). mp: 197-199 ℃.

$^1$H NMR (600 MHz, DMSO-$d_6$) δ 8.21 (s, 1 H), 7.54 (s, 2 H), 7.14 - 7.27 (m, 2 H), 6.73 - 6.84 (m, 2 H), 5.99 (s, 1 H), 3.84 (t, *J*=6.5 Hz, 2 H), 3.77 (s, 3 H), 2.71 (t, *J*=6.9 Hz, 2 H), 1.60 - 1.76 (m, 2 H), 0.96 (t, *J*=7.4 Hz, 3 H).

$^{13}$C NMR (150 MHz, DMSO-$d_6$) δ 155.7 (C=O), 153.7 (=C-O), 133.8 (=C-O), 133.4 (C-N), 119.9 (C-N), 117.6 (C-Br), 114.9 (-C=), 69.4 (-O-CH3), 60.7 (CH2), 34.7 (CH2), 22.5 (CH2), 10.8 (CH3).

*1- (3,5-dibromo-4-methoxyphenyl) -3- (4-butoxyphenyl) urea (6d)*

Following the general procedure, 1d (0.4 g, 1.82 mmol)gave 6d as a white solid (0.50 g, 55% yield).mp: 150-156 ℃.

$^{1}$H NMR (600 MHz, DMSO-$d_6$) δ 8.21 (s, 1 H), 7.54 (s, 2 H), 7.19 - 7.28 (d, *J*=9.0 Hz, 2 H), 6.74 - 6.89 (d, *J*=9.0 Hz, 2 H), 6.00 (t, *J*=5.6 Hz, 1 H), 3.88 (t, *J*=6.5 Hz, 2 H), 3.77 (s, 3 H), 3.27 - 3.32 (m, 2 H), 2.71 (t, *J*=6.8 Hz, 2 H), 1.59 - 1.71 (m, 2 H), 1.42 (sxt, *J*=7.4 Hz, 2 H), 0.92 (t, *J*=7.3 Hz, 3 H)

$^{13}$C NMR (150 MHz, DMSO-$d_6$) δ 155.7 (C=O), 153.8 (=C-O), 152.0 (=C-O), 139.8 (C-N), 133.4 (C-N), 119.9 (C-Br), 117.6 (-C=), 114.9 (-C=), 67.6 (-O-CH3), 60.7 (CH2), 40.5 (CH2), 34.7 (CH2), 31.2 (CH2), 19.1 (CH2), 14.1 (CH3).

*1- (3,5-dibromo-4-methoxyphenyl) -3- (4-secutoxyphenyl) urea (6e)*

Following the general procedure, 1e (0.4 g, 1.82 mmol)1egave 6e as a white solid (0.49 g, 54% yield). mp: 160-164 ℃.

$^{1}$H NMR (600 MHz, DMSO-$d_6$) δ 8.21 (s, 1 H), 7.43 - 7.60 (m, 2 H), 7.16 - 7.31 (d, *J*=9.0 Hz, 2 H), 6.69 - 6.89 (d, *J*=8.8 Hz, 2 H), 6.00 (t, *J*=5.6 Hz, 1 H), 4.19 - 4.29 (m, 1 H), 3.77 (s, 3 H), 3.30 - 3.33 (m, 2 H), 2.71 (t, *J*=6.9 Hz, 2 H), 1.46 - 1.66 (m, 2 H), 1.18 (d, *J*=6.0 Hz, 3 H), 0.90 (t, *J*=7.4 Hz, 3 H)

$^{13}$C NMR (150 MHz, DMSO-$d_6$) δ 155.8 (C=O), 153.7 (=C-O), 152.0 (=C-O), 139.8 (C-N), 133.8 (C-N), 133.4 (C-Br), 119.9 (-C=), 67.9 (-O-CH3), 60.7 (CH2), 40.5 (CH2), 34.7 (CH2), 28.9 (CH2), 28.1 (CH2), 22.3 (CH3), 14.3 (CH3).

*1- (3,5-dibromo-4-methoxyphenyl) -3- (4-n-pentylphenyl) urea (6f)*

Following the general procedure, 1f (0.4 g, 1.71 mmol)1fgave 6f as a white solid (0.35 g, 40% yield). mp: 184-186 ℃.

$^{1}$H NMR (600 MHz, DMSO-$d_6$) δ 8.23 (s, 1 H), 7.47 - 7.62 (m, 2 H), 7.17 - 7.30 (m, 2 H), 6.71 - 6.86 (m, 2 H), 6.02 (t, *J*=4.7 Hz, 1 H), 3.87 (t, *J*=6.5 Hz, 2 H), 3.73 - 3.81 (m, 3 H), 3.30 - 3.33 (m, 2 H), 2.71 (t, *J*=6.9 Hz, 2 H), 1.63 - 1.70 (m, 2 H), 1.28 - 1.42 (m, 4 H), 0.89 (t, *J*=7.2 Hz, 3 H).

$^{13}$C NMR (150 MHz, DMSO-$d_6$) δ 155.8 (C=O), 153.7 (=C-O), 133.9 (=C-O), 132.6 (C-N), 119.8 (C-N), 114.9 (C-Br), 112.5 (-C=), 67.9 (-O-CH3), 60.7 (CH2), 34.7 (CH2), 28.9 (CH2), 28.1 (CH2), 22.3 (CH2), 14.3 (CH3).

*1- (3,5-dibromo-4-methoxyphenyl) -3- [4- (3-morpholinopropyl) phenyl] urea (6g)*

Following the general procedure, 1g (0.4 g, 1.81 mmol)1ggave 6g as a white solid (0.44 g, 48% yield). m.p .: 169-172 ℃.

$^{1}$H NMR (600 MHz, DMSO-$d_6$) δ 8.44 (s, 1 H), 8.19 (br. s., 1 H), 7.45 (s, 2 H), 7.39 (s, 2 H), 7.20 - 7.25 (d, *J*=8.5 Hz, 2 H), 6.71 - 6.85 (d, *J*=8.5 Hz, 2 H), 6.28 (d, *J*=8.5 Hz, 1 H), 4.32 - 4.48 (m, 1 H), 3.75 (s, 3 H), 3.68 (s, 3 H), 3.26 - 3.35 (m, 1 H), 3.20 (dd, *J*=12.1, 5.7 Hz, 1 H), 2.85 (dd, *J*=13.5, 4.0 Hz, 1 H), 2.69 - 2.78 (m, 1 H), 2.60 (d, *J*=4.9 Hz, 2 H).

$^{13}$C NMR (150 MHz, DMSO-$d_6$) δ 155.7 (C=O), 153.5 (=C-O), 149.3 (=C-O), 134.6 (C-N), 132.7 (C-N), 119.8 (C-Br), 114.9 (-C=), 66.1 (-O-CH3), 55.0 (CH2), 34.5 (CH2).

*1- (3,5-dibromo-4-methoxyphenyl) -3- (4-isopropylphenyl) urea (6h)*

Following the general procedure, 1h (0.4 g, 1.95 mmol)gave 6h as a white solid (0.53 g, 56% yield). m.p.:166-168℃.

$^{1}H$ NMR (600 MHz, DMSO-$d_6$) δ 8.24 (s, 1 H), 7.47 - 7.57 (m, 2 H), 7.16 - 7.35 (d, *J*=8.8 Hz, 2 H), 6.72 - 6.85 (d, *J*=9.0 Hz, 2 H), 6.02 (t, *J*=5.6 Hz, 1 H), 4.47 (dt, *J*=12.0, 6.0 Hz, 1 H), 3.68 - 3.84 (m, 3 H), 3.30 - 3.34 (m, 2 H), 2.71 (t, *J*=6.9 Hz, 2 H), 1.22 (d, *J*=6.0 Hz, 6 H).

$^{13}C$ NMR (150 MHz, DMSO-$d_6$) δ 155.7 (C=O), 153.7 (=C-O), 149.3 (=C-O), 133.9 (C-N), 132.7 (C-N), 119.8 (C-Br), 114.9 (-C=), 69.4 (-O-CH3), 60.7 (CH2), 34.5 (CH2), 22.5 (CH2), 10.8 (CH3).

***1- (3,5-dibromo-4-methoxyphenyl) -3- [4- (3-chlorophenyl)] urea (6i)***

Following the general procedure, 1i (0.4 g, 2.20 mmol)1igave 6i as a white solid (0.49 g, 48% yield). mp: 169-172 ℃.

$^{1}H$ NMR (600 MHz, DMSO-$d_6$) δ 8.44 (s, 1 H), 8.19 (br. s., 1 H), 7.45 (s, 2 H), 7.39 (s, 2 H), 7.20 - 7.25 (d, *J*=8.5 Hz, 2 H), 6.71 - 6.85 (d, *J*=8.5 Hz, 2 H), 6.28 (d, *J*=8.5 Hz, 1 H), 4.32 - 4.48 (m, 1 H), 3.75 (s, 3 H), 3.68 (s, 3 H), 3.26 - 3.35 (m, 1 H), 3.20 (dd, *J*=12.1, 5.7 Hz, 1 H), 2.85 (dd, *J*=13.5, 4.0 Hz, 1 H), 2.69 - 2.78 (m, 1 H), 2.60 (d, *J*=4.9 Hz, 2 H).

$^{13}C$ NMR (150 MHz, DMSO-$d_6$) δ 152.9 (C=O), 140.0 (=C-O), 138.3 (C-N), 129.0 (C-N), 122.9 (C-Cl), 119.0 (C-Br), 115.7 (-C=), 21.6 (CH2).

***1- (3,5-dibromo-4-methoxyphenyl) -3- [4- (2-chlorophenyl)] urea (6j)***

Following the general procedure, 1j (0.4 g, 2.20 mmol)1jgave 6j as a white solid (0.53 g, 52% yield). mp: 211-215 ℃.

$^{1}H$ NMR (600 MHz, DMSO-$d_6$) δ 8.09 (dd, *J*=8.3, 1.3 Hz, 1 H), 8.00 (s, 1 H), 7.56 (s, 2 H), 7.38 (dd, *J*=8.0, 1.4 Hz, 1 H), 7.20 - 7.26 (m, 1 H), 6.99 (t, *J*=5.6 Hz, 1 H), 6.95 (td, *J*=7.7, 1.6 Hz, 1 H), 3.77 (s, 3 H), 3.33 - 3.38 (m, 2 H), 2.73 (t, *J*=6.9 Hz, 2 H).

$^{13}C$ NMR (150 MHz, DMSO-$d_6$) δ 155.1 (C=O), 152.0 (=C-O), 139.6 (C-N), 137.0 (C-N), 133.4(C-Cl), 129.4 (C-Br), 127.8 (-C=), 122.9 (-C=), 60.7 (-O-CH3), 34.5 (CH2).

***1- (3,5-dibromo-4-methoxyphenyl) -3- [4- (4-bromophenyl)] urea (6k)***

Following the general procedure, 1k (0.4 g, 1.77 mmol)1k 6k as a white solid (0.37 g, 41% yield). 74% yield, mp: 204-207 ℃.

$^{1}H$ NMR (600 MHz, DMSO-$d_6$) δ 8.59 (s, 1 H), 7.54 (s, 2 H), 7.32 - 7.39 (m, 4 H), 6.17 (t, *J*=5.6 Hz, 1 H), 3.77 (s, 3 H), 3.30 - 3.33 (m, 2 H), 2.72 (t, *J*=6.9 Hz, 2 H).

$^{13}C$ NMR (150 MHz, DMSO-$d_6$) δ 155.2 (C=O), 152.0 (=C-O), 139.6 (C-N), 133.4 (C-N), 128.6 (C-Br), 124.7 (C-Br), 117.6 (-C=), 115.2 (-C=), 55.4 (-O-CH3), 34.6 (CH2).

***1- (3,5-dibromo-4-methoxyphenyl) -3- [4- (4-fluorophenyl)] urea (6l)***

Following the general procedure, 1l (0.4 g, 2.42 mmol)1lgave 6l as a white solid (0.49 g, 45% yield). mp: 191-195 ℃.

$^{1}H$ NMR (600 MHz, DMSO-$d_6$) δ 8.44 (s, 1 H), 8.19 (br. s., 1 H), 7.45 (s, 2 H), 7.39 (s, 2 H), 7.20 - 7.25 (d, *J*=8.5 Hz, 2 H), 6.71 - 6.85 (d, *J*=8.5 Hz, 2 H), 6.28 (d, *J*=8.5 Hz, 1 H), 4.32 - 4.48 (m, 1 H), 3.75 (s, 3 H), 3.68 (s, 3 H), 3.26 - 3.35 (m, 1 H), 3.20 (dd, *J*=12.1, 5.7 Hz, 1 H), 2.85 (dd, *J*=13.5, 4.0 Hz, 1 H), 2.69 - 2.78 (m, 1 H), 2.60 (d, *J*=4.9 Hz, 2 H).

$^{13}C$ NMR (150 MHz, DMSO-$d_6$) δ 155.6 (C=O), 141.0 (=C-O), 138.0 (C-N), 132.2 (C-N), 128.8 (C-F), 122.0 (C-Br), 118.6 (-C=), 115.2 (-C=), 67.9 (-O-CH3), 60.4 (CH2), 34.7 (CH2), 22.3 (CH2).

***1- (3,5-dibromo-4-methoxyphenyl) -3- [4- (2-fluorophenyl)] urea (6m)***

Following the general procedure, 1m (0.4 g, 2.42 mmol)1m gave 6m as a white solid (0.55 g, 51% yield). mp: 190-196 ℃.

$^{1}$H NMR (600 MHz, DMSO-$d_6$) δ 8.24 - 8.33 (m, 1 H), 8.10 (t, $J$=7.7 Hz, 1 H), 7.51 - 7.61 (m, 2 H), 7.16 (ddd, $J$=11.7, 8.2, 1.3 Hz, 1 H), 7.00 - 7.09 (m, 1 H), 6.87 - 6.95 (m, 1 H), 6.59 (t, $J$=5.6 Hz, 1 H), 3.69 - 3.81 (m, 3 H), 3.35 (q, $J$=6.8 Hz, 2 H), 2.72 (t, $J$=6.8 Hz, 2 H).

$^{13}$C NMR (150 MHz, DMSO-$d_6$) δ 155.1 (C=O), 152.0 (=C-O), 139.6 (C-N), 137.0 (C-N), 133.4(C-F), 129.4 (C-Br), 127.8 (-C=), 122.9 (-C=), 60.7 (-O-CH3), 34.5 (CH2).

*1- (3,5-dibromo-4-methoxyphenyl) -3- [4- (4-methylphenyl)] urea (6n)*

Following the general procedure, 1n (0.4 g, 2.48 mmol)1ngave 6n as a white solid (0.51 g, 46% yield). mp: 205-210 ℃.

$^{1}$H NMR (600 MHz, DMSO-$d_6$) δ 8.44 (s, 1 H), 8.19 (br. s., 1 H), 7.45 (s, 2 H), 7.39 (s, 2 H), 7.20 - 7.25 (d, $J$=8.5 Hz, 2 H), 6.71 - 6.85 (d, $J$=8.5 Hz, 2 H), 6.28 (d, $J$=8.5 Hz, 1 H), 4.32 - 4.48 (m, 1 H), 3.75 (s, 3 H), 3.68 (s, 3 H), 3.26 - 3.35 (m, 1 H), 3.20 (dd, $J$=12.1, 5.7 Hz, 1 H), 2.85 (dd, $J$=13.5, 4.0 Hz, 1 H), 2.69 - 2.78 (m, 1 H), 2.60 (d, $J$=4.9 Hz, 2 H).

$^{13}$C NMR (150 MHz, DMSO-$d_6$) δ 153.0 (C=O), 137.6 (=C-O), 130.9 (C-N), 129.5 (C-N), 118.6 (C-Br), 53.2, 20.7 (CH3).

*1- (3,5-dibromo-4-methoxyphenyl) -3- [4- (2-methylethyl ether)] urea (6o)*

Following the general procedure, 1o (0.4 g, 1.38 mmol) 1ogave 6o as a white solid (0.37 g, 47% yield).m.p.:201-209 ℃

$^{1}$H NMR (600 MHz, DMSO-$d_6$) δ 8.44 (s, 1 H), 8.19 (br. s., 1 H), 7.45 (s, 2 H), 7.39 (s, 2 H), 7.20 - 7.25 (d, $J$=8.5 Hz, 2 H), 6.71 - 6.85 (d, $J$=8.5 Hz, 2 H), 6.28 (d, $J$=8.5 Hz, 1 H), 4.32 - 4.48 (m, 1 H), 3.75 (s, 3 H), 3.68 (s, 3 H), 3.26 - 3.35 (m, 1 H), 3.20 (dd, $J$=12.1, 5.7 Hz, 1 H), 2.85 (dd, $J$=13.5, 4.0 Hz, 1 H), 2.69 - 2.78 (m, 1 H), 2.60 (d, $J$=4.9 Hz, 2 H).

$^{13}$C NMR (150 MHz, DMSO-$d_6$) δ 155.7 (C=O), 153.7 (=C-O), 149.3 (=C-O), 134.6 (C-N), 133.9 (C-N), 132.7 (C-Br), 119.8 (-C=), 71.9 (-O-CH3), 67.6 (-O-CH3), 34.5(CH2).

**4. spectrum of the derivatives**

*4.1*

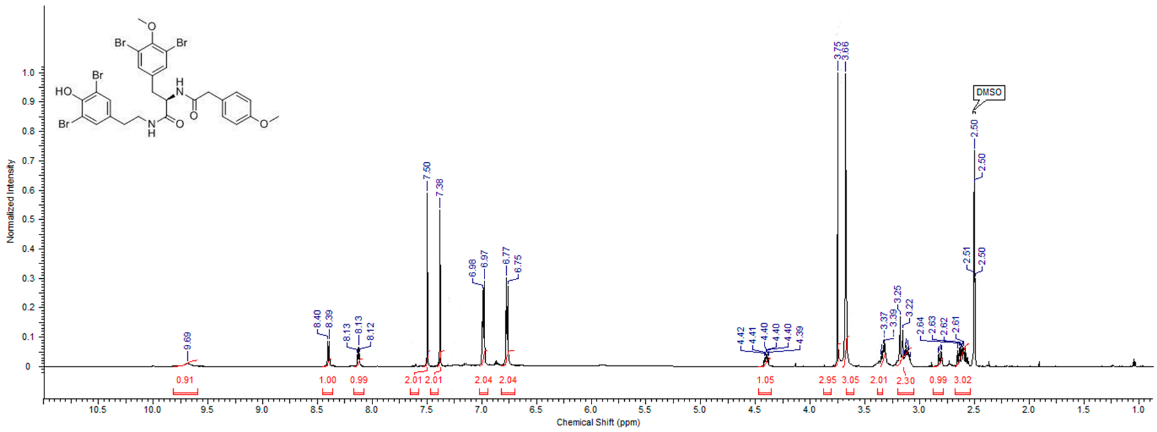


**4A $^{1}$H-NMR**

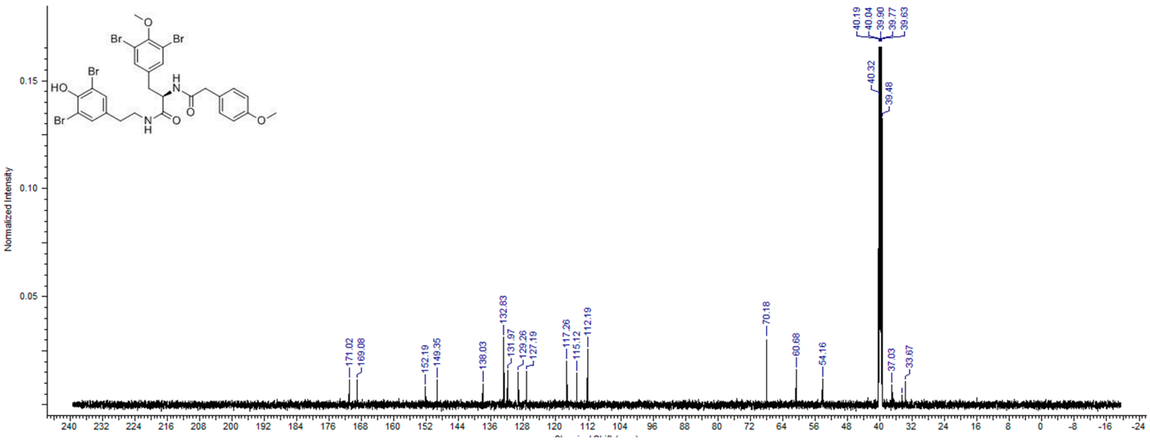


4A $^{13}$C-NMR

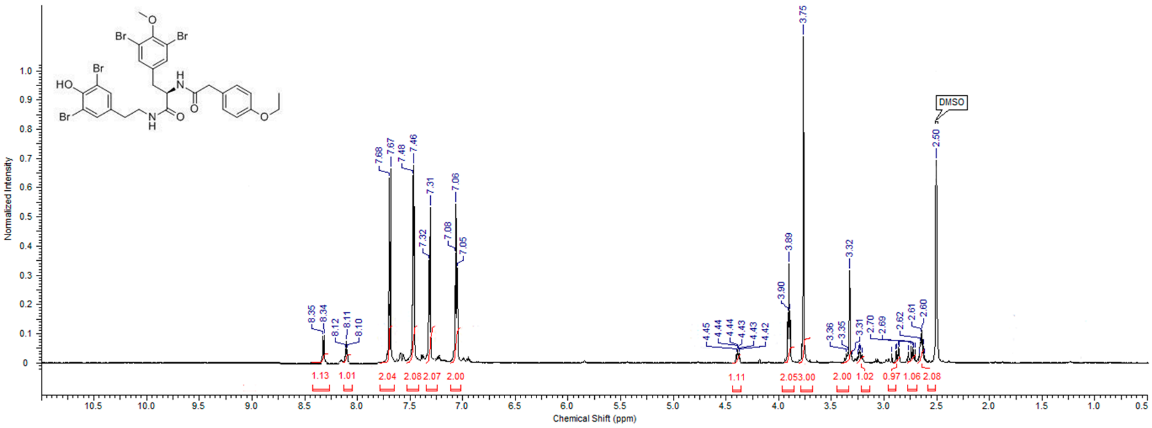


4B $^{1}$H-NMR

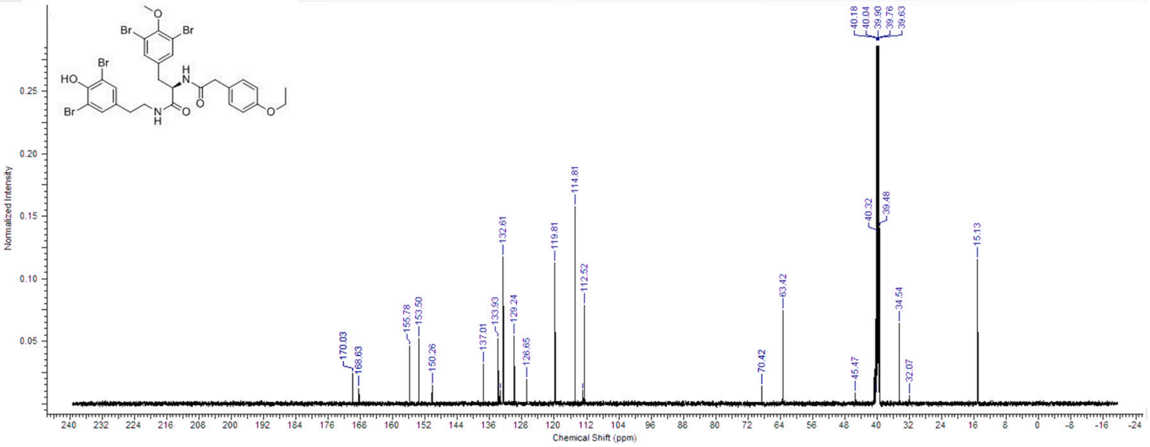


4B $^{13}$C-NMR

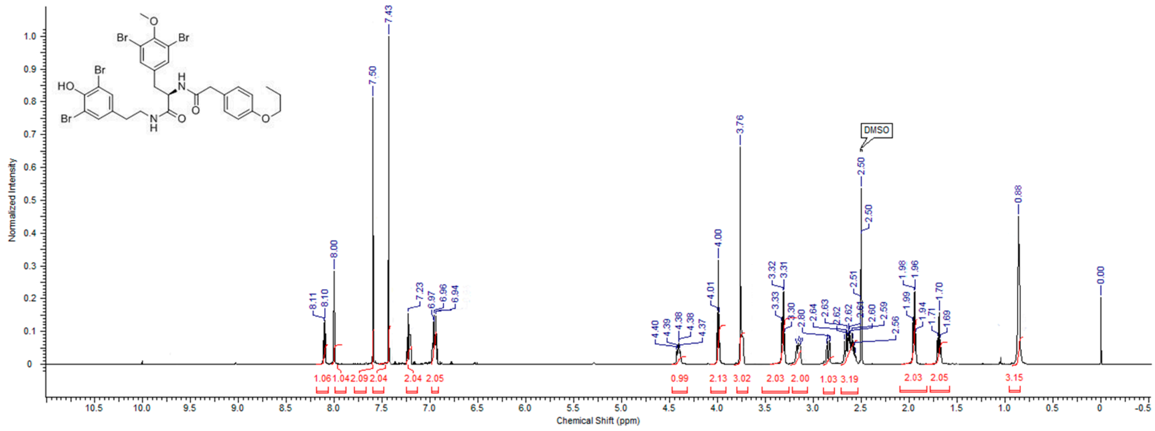

**4C $^{1}$H-NMR**

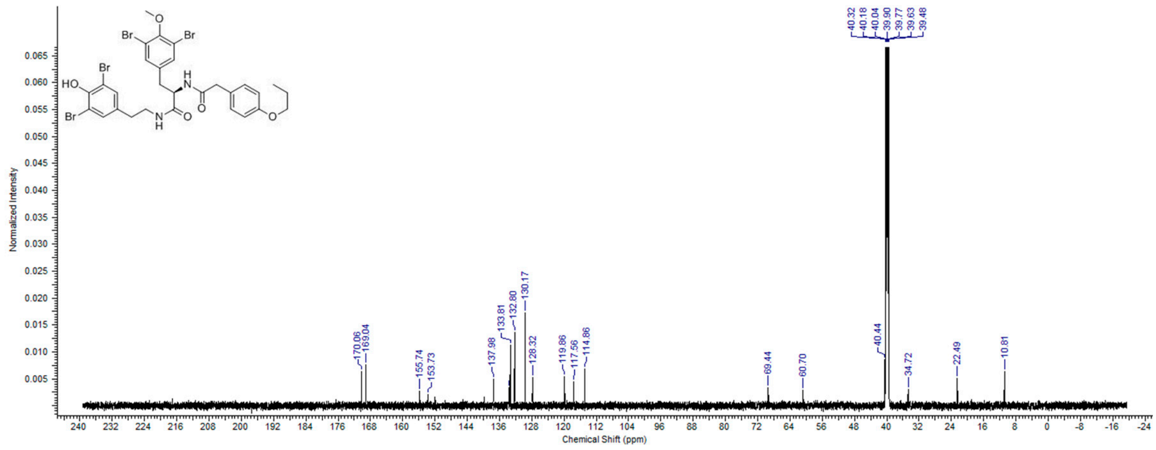

**4C $^{13}$C-NMR**

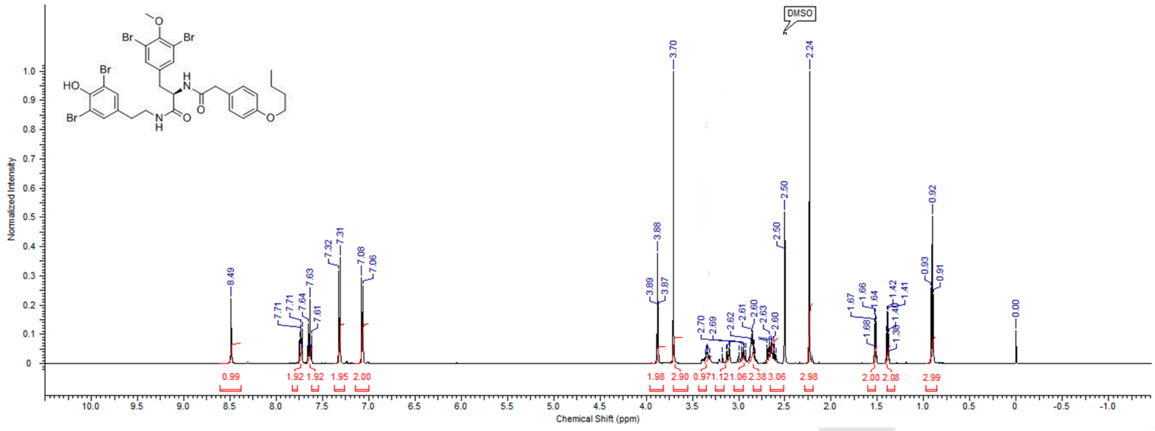

**4D $^{1}$H-NMR**

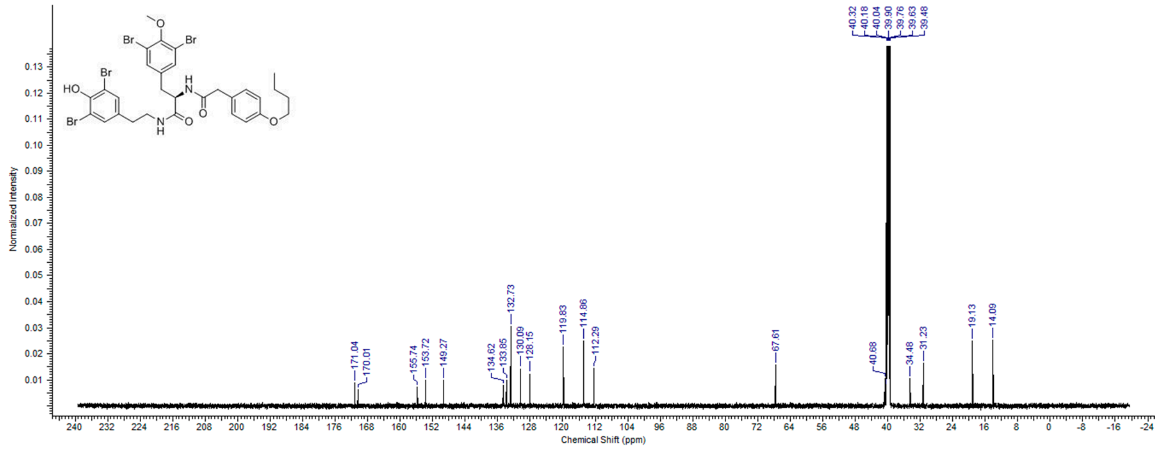

**4D $^{13}$C-NMR**

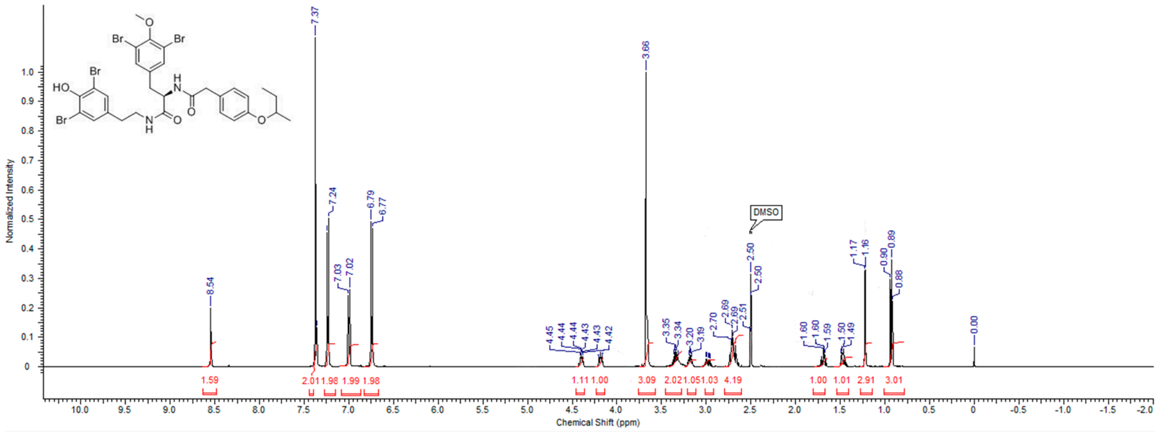

**4E $^{1}$H-NMR**

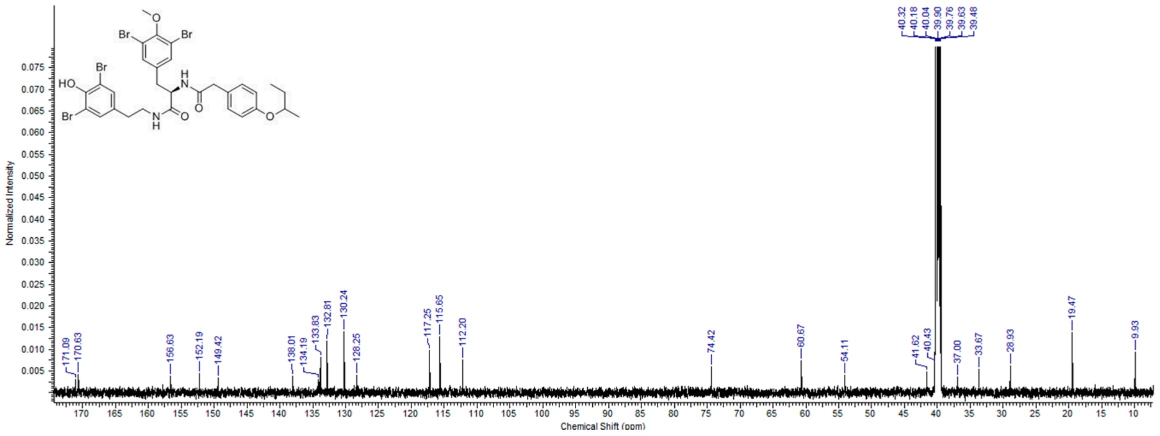

**4E $^{13}$C-NMR**

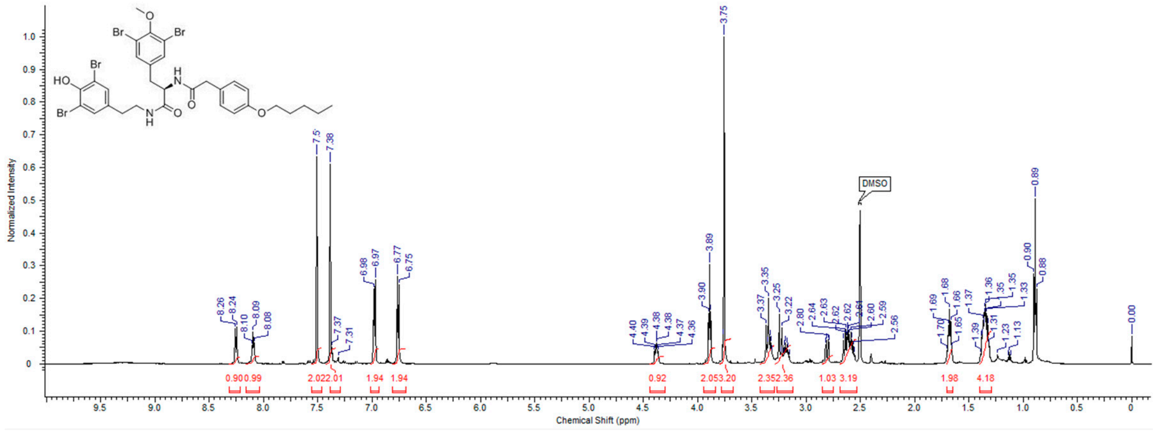

**4F $^{1}$H-NMR**

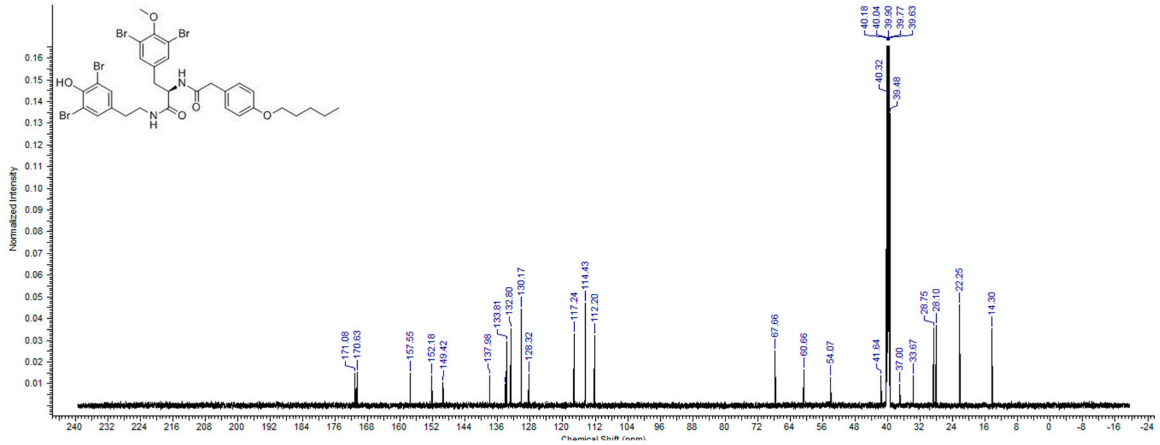

**4F $^{13}$C-NMR**

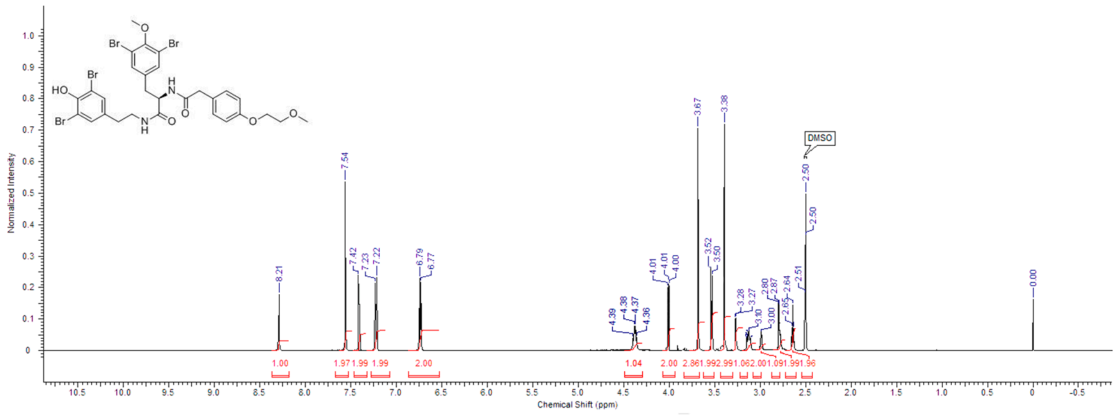

**4G $^{1}$H-NMR**

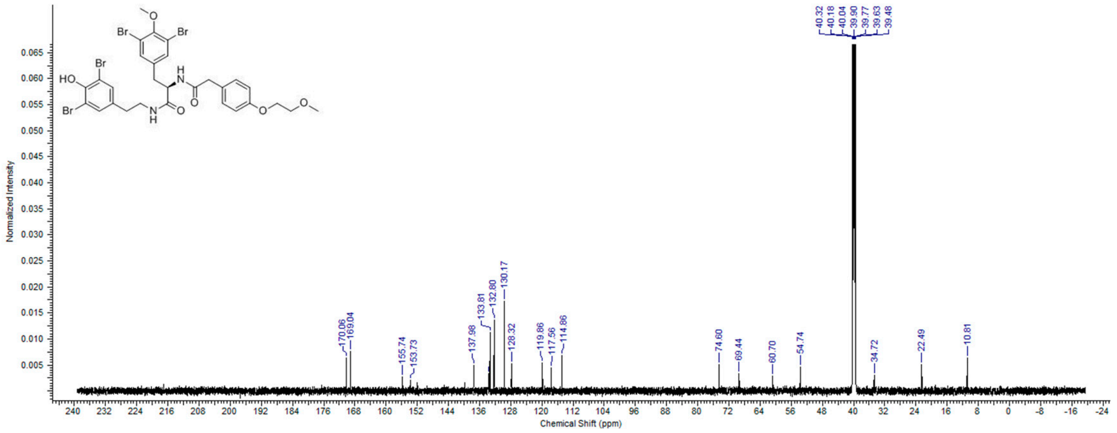

**4G $^{13}$C-NMR**

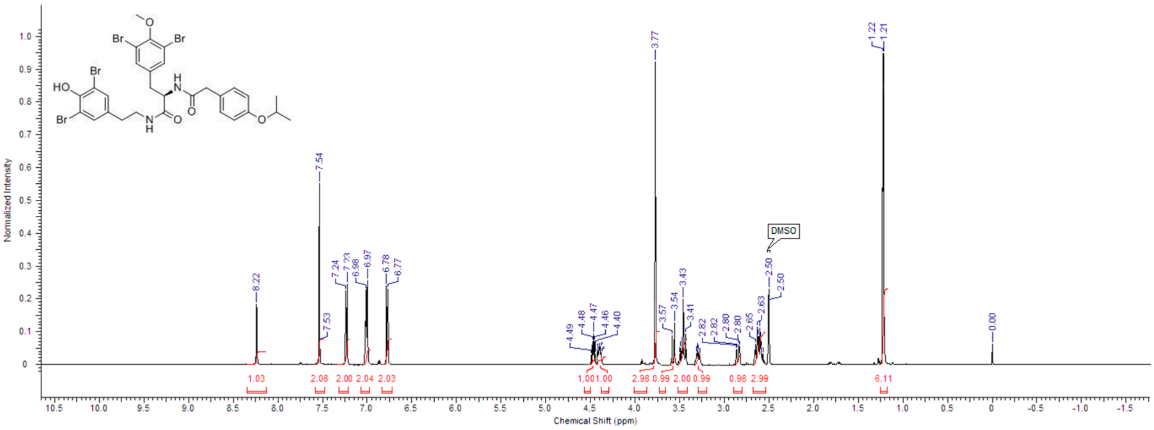

**4H $^{1}$H-NMR**

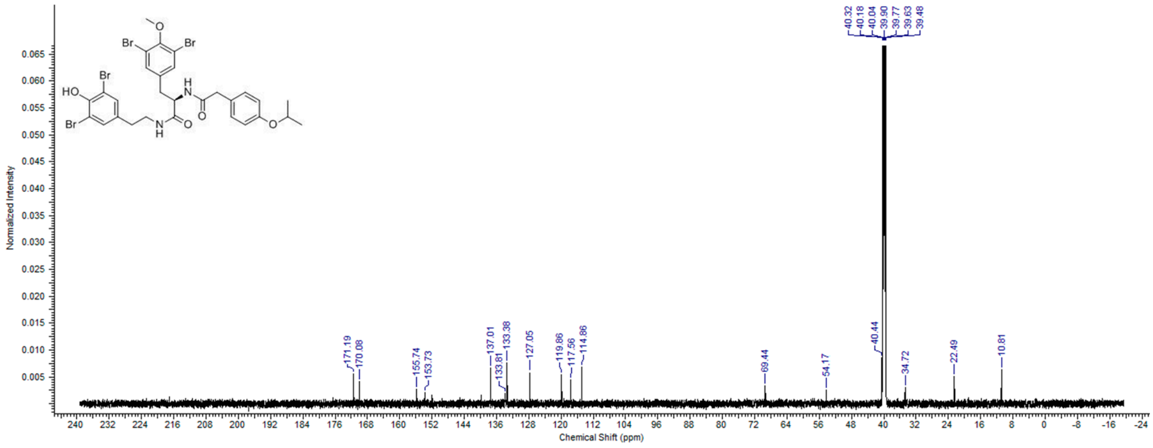

**4H $^{13}$C-NMR**

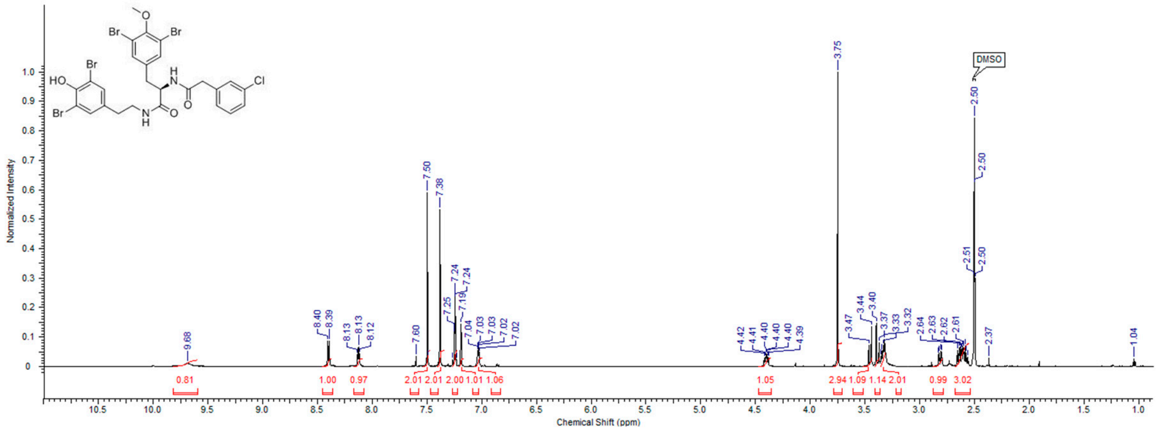

**4I $^{1}$H-NMR**

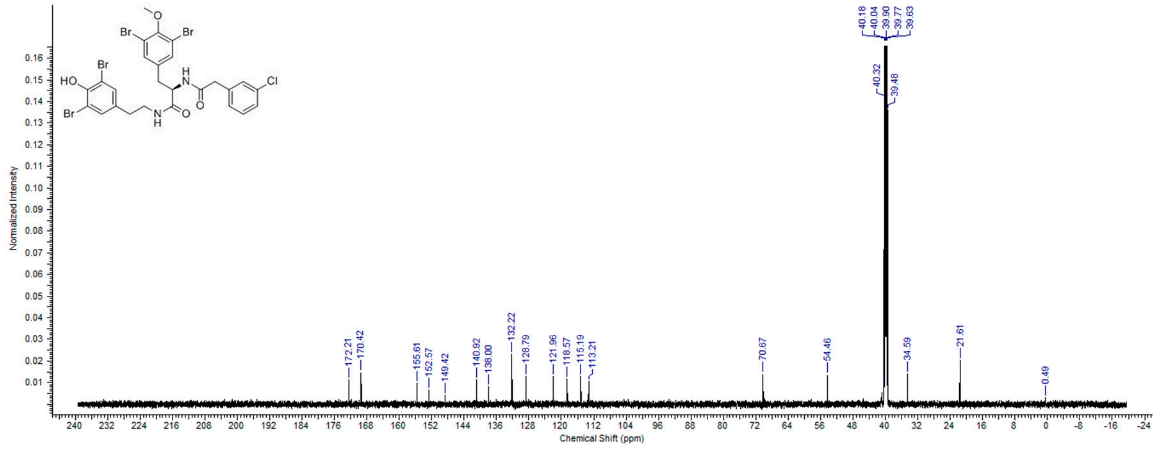

**4I $^{13}$C-NMR**

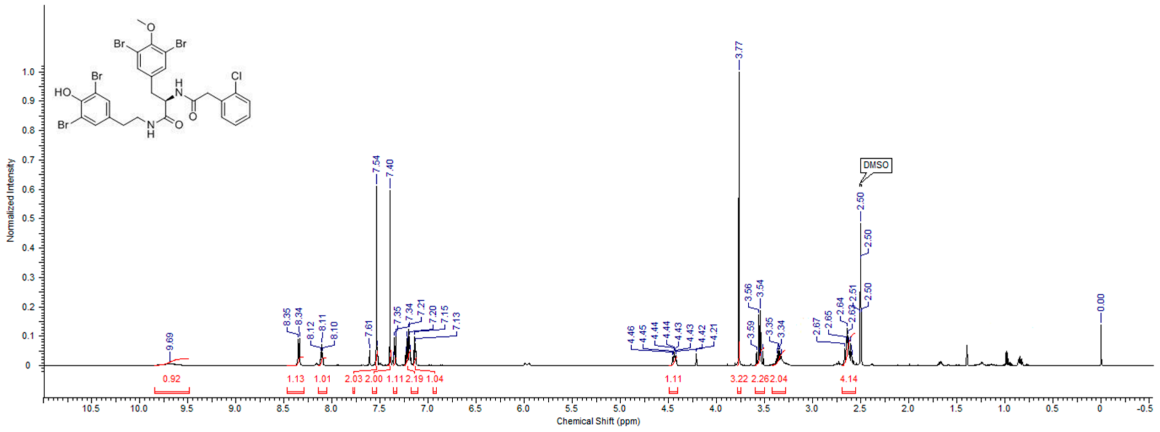

**4J $^{1}$H-NMR**

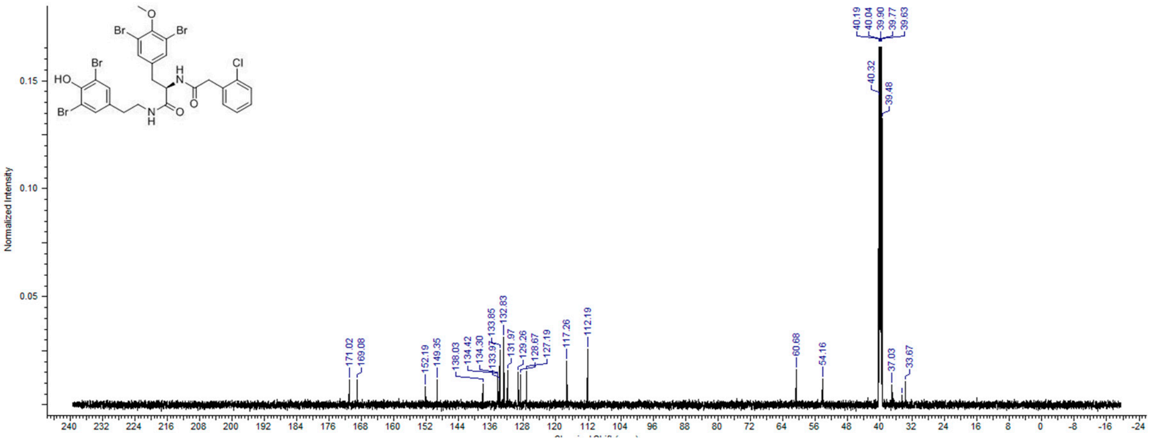


**4J $^{13}$C-NMR**

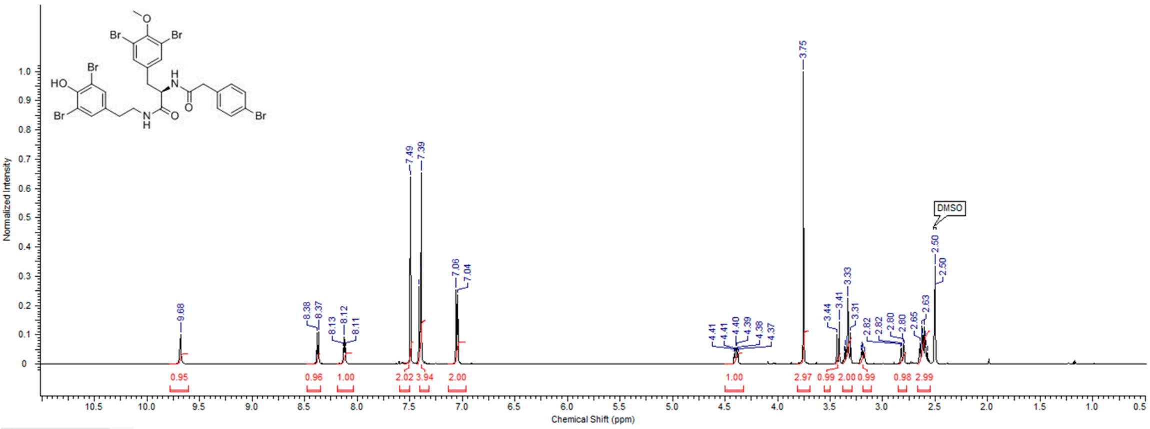


**4K $^{1}$H-NMR**

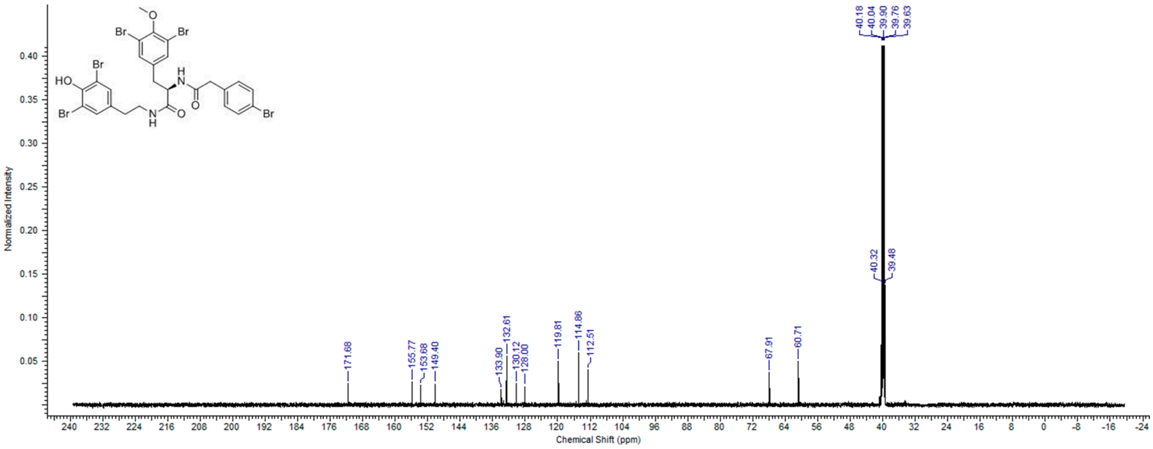


**4K $^{13}$C-NMR**

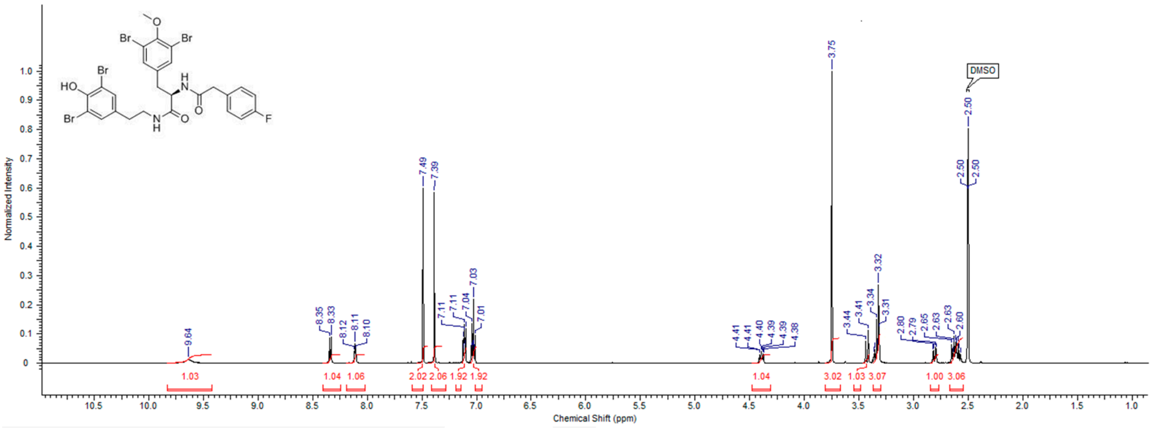


**4L $^{1}$H-NMR**

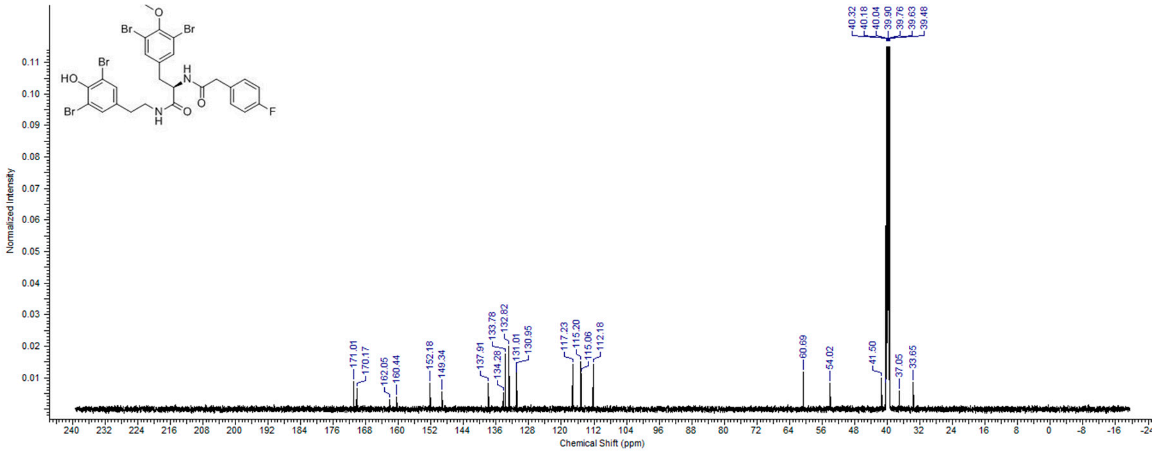


**4L $^{13}$C-NMR**

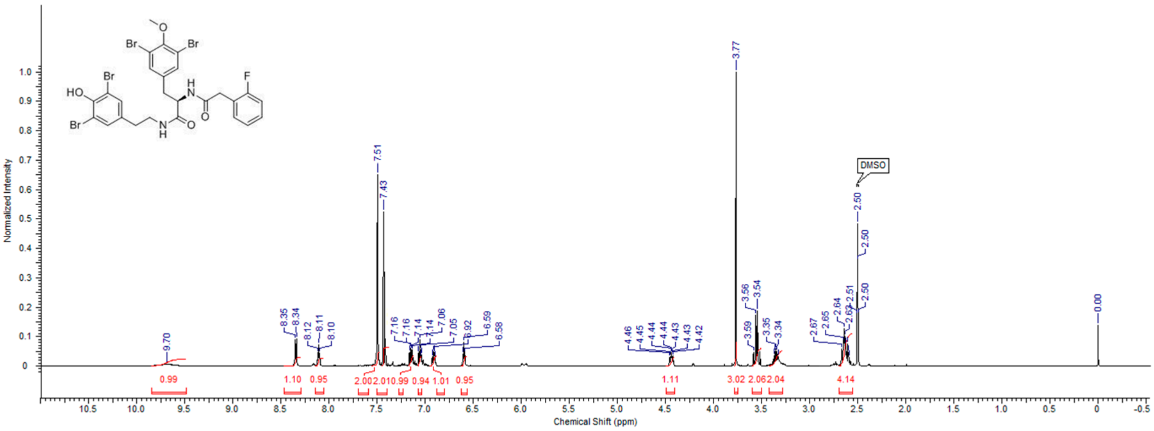


**4M $^{1}$H-NMR**

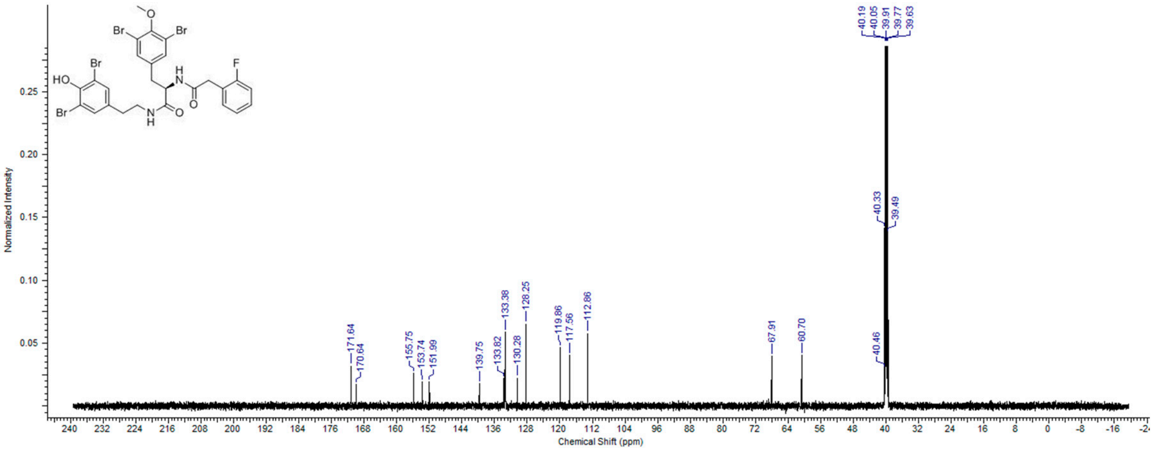


**4M $^{13}$C-NMR**

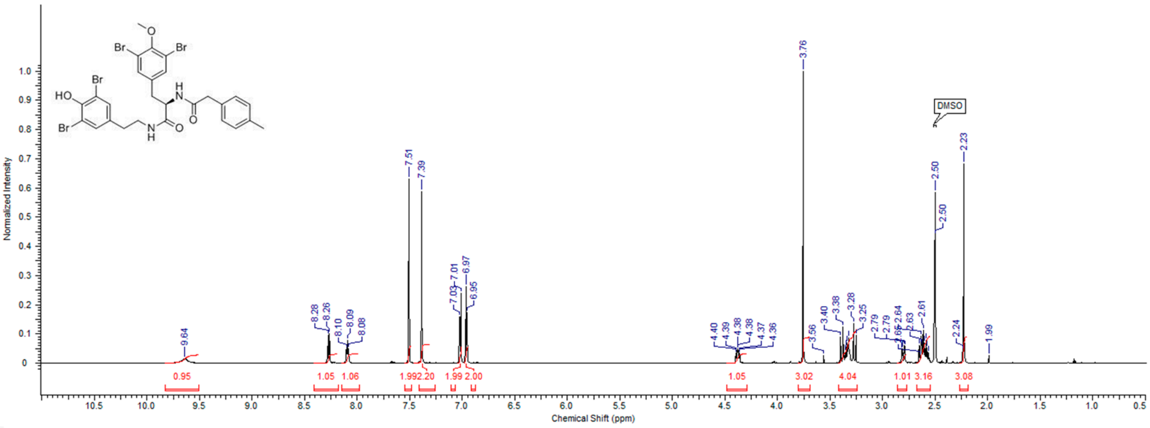


**4N $^{1}$H-NMR**

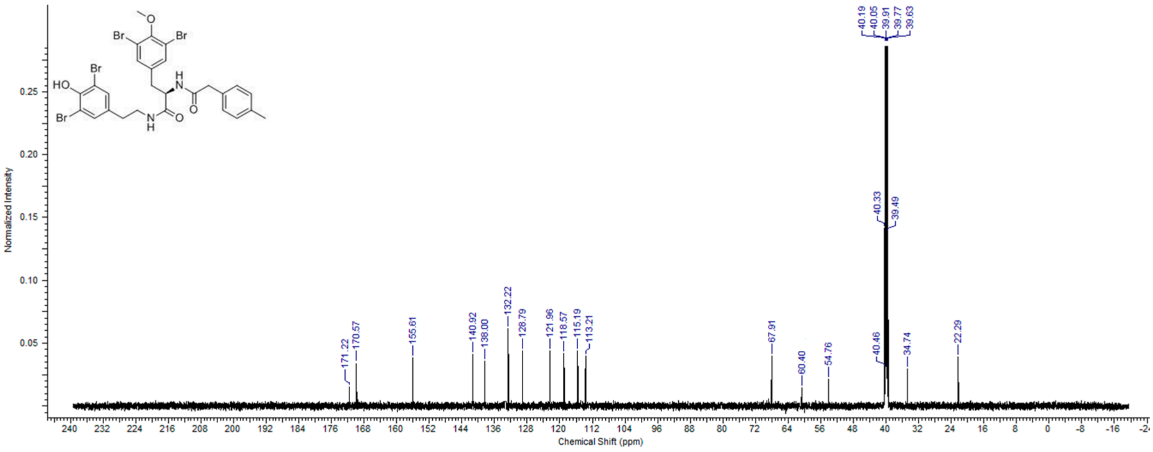


**4N $^{13}$C-NMR**

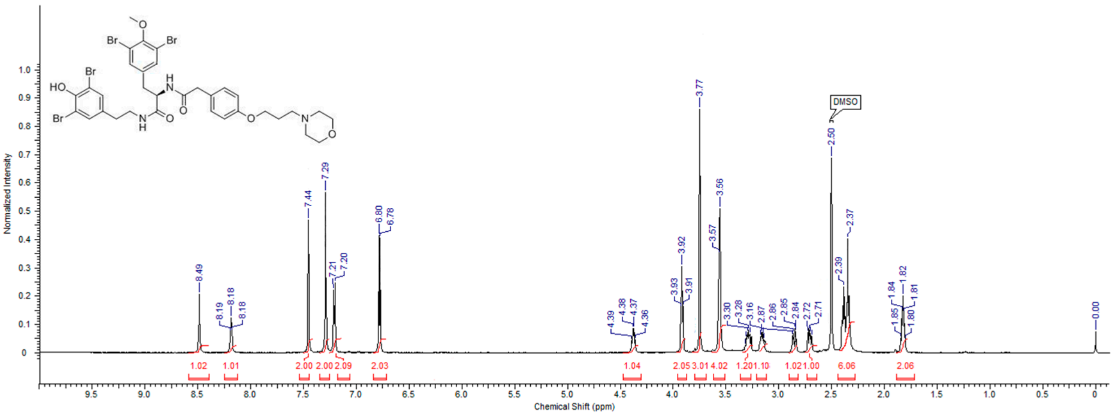


**4O $^{1}$H-NMR**

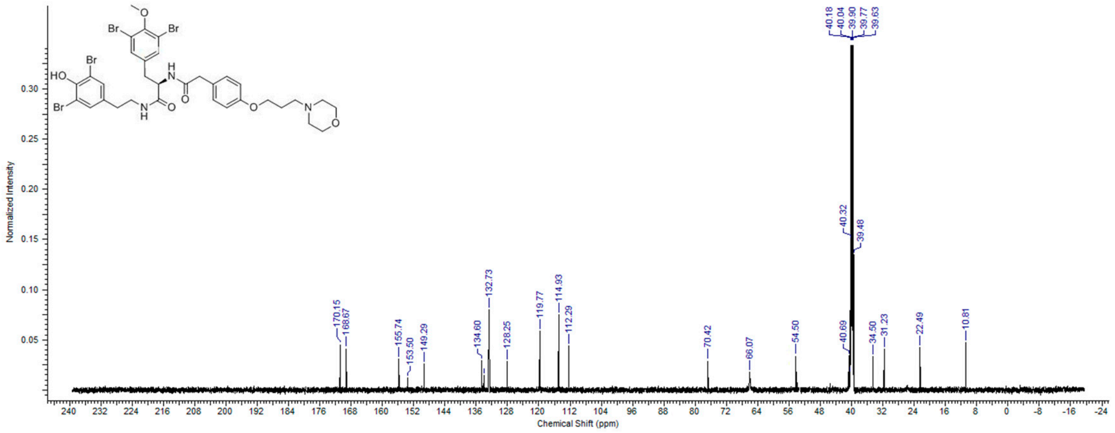


**4O $^{13}$C-NMR**

*2.2*

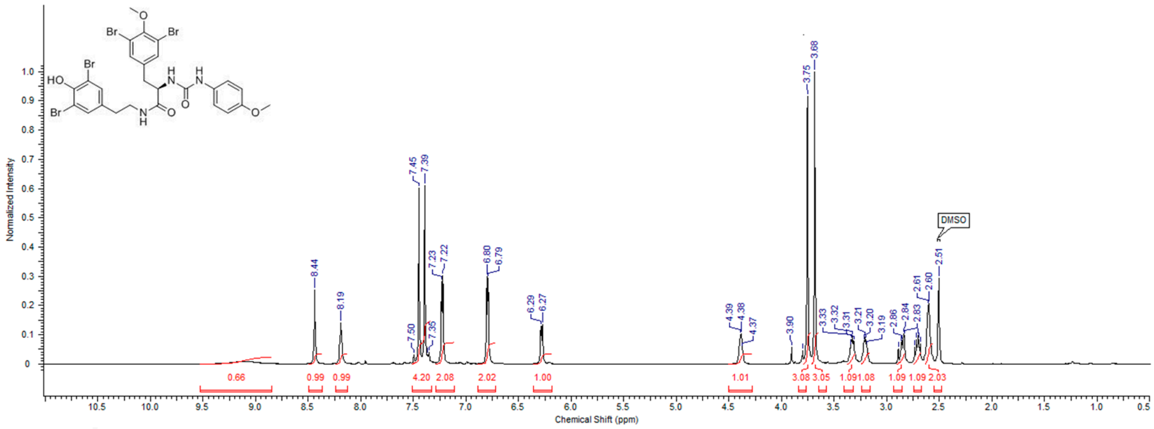


**5A $^{1}$H-NMR**

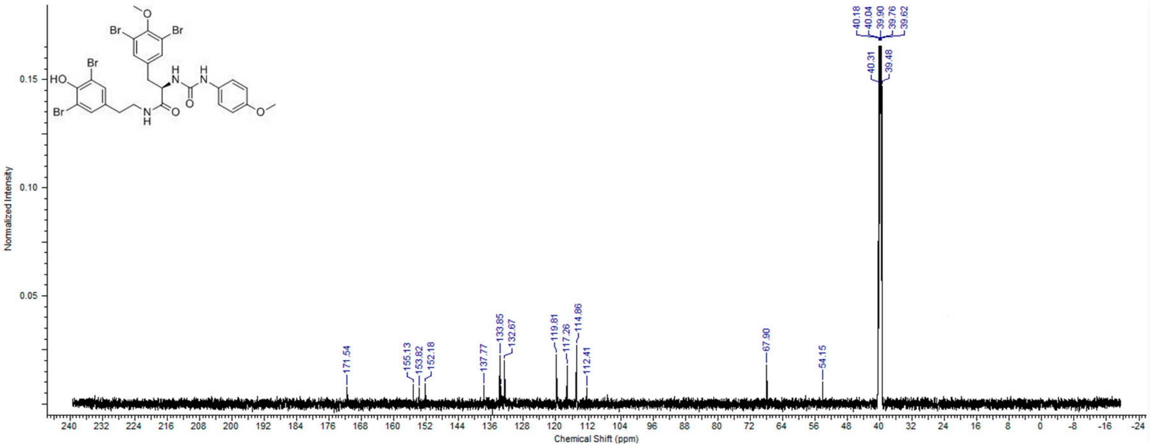

**5A $^{13}$C-NMR**

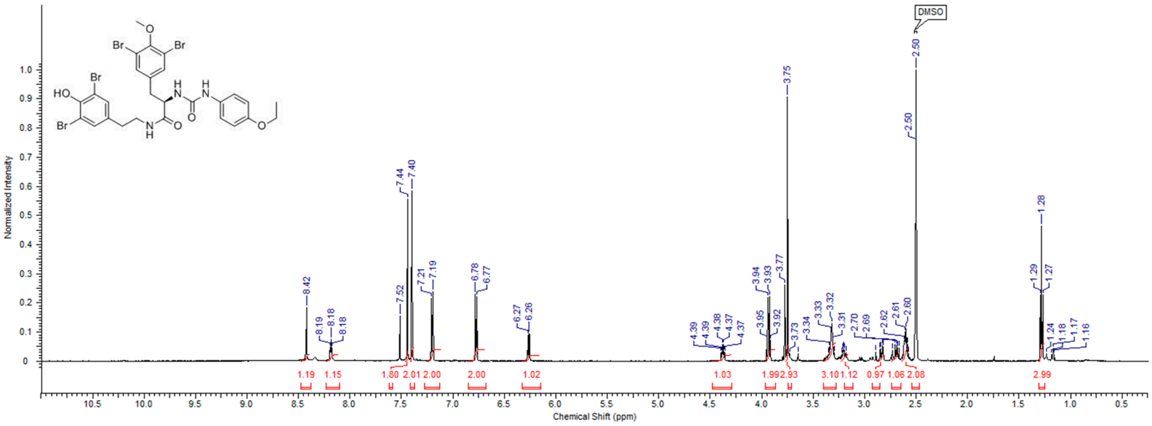

**5B $^{1}$H-NMR**

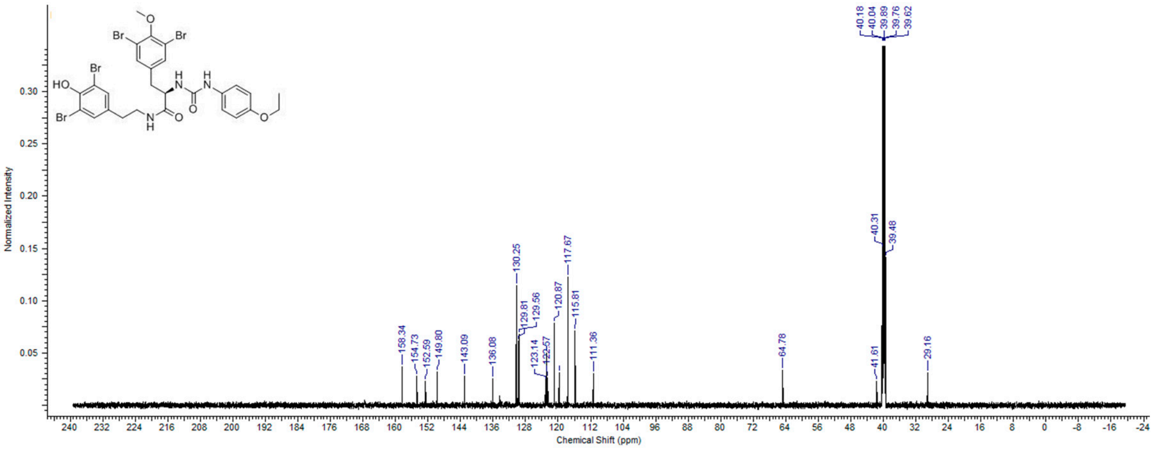

**5B $^{13}$C-NMR**

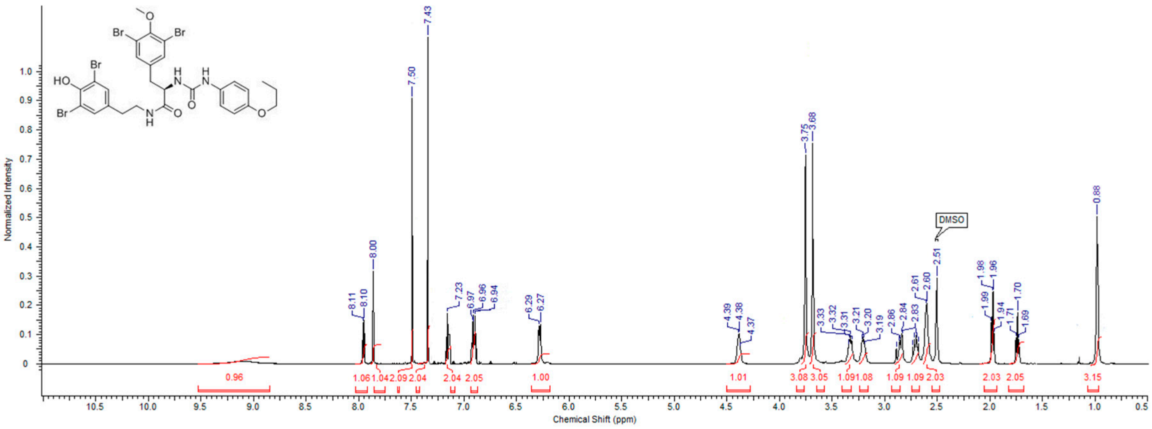


**5C $^{1}$H-NMR**

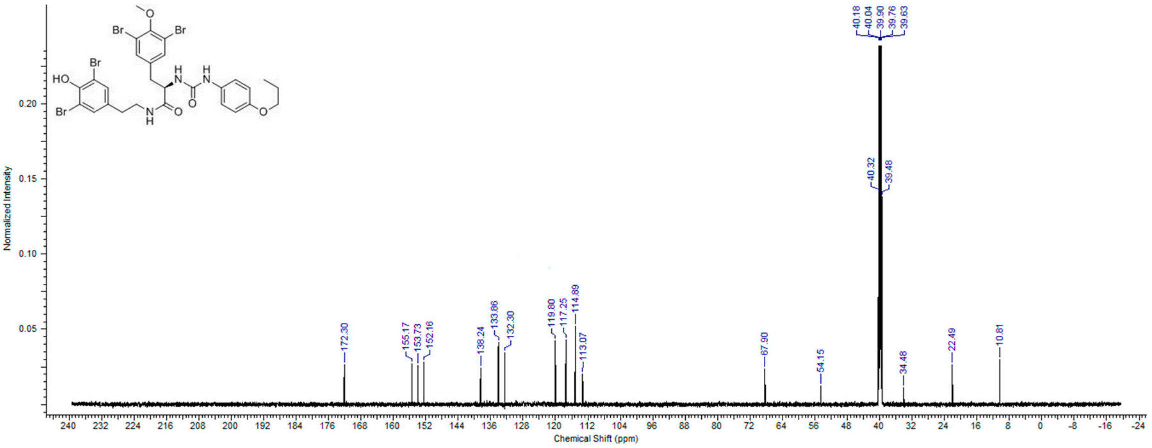


**5C $^{13}$C-NMR**

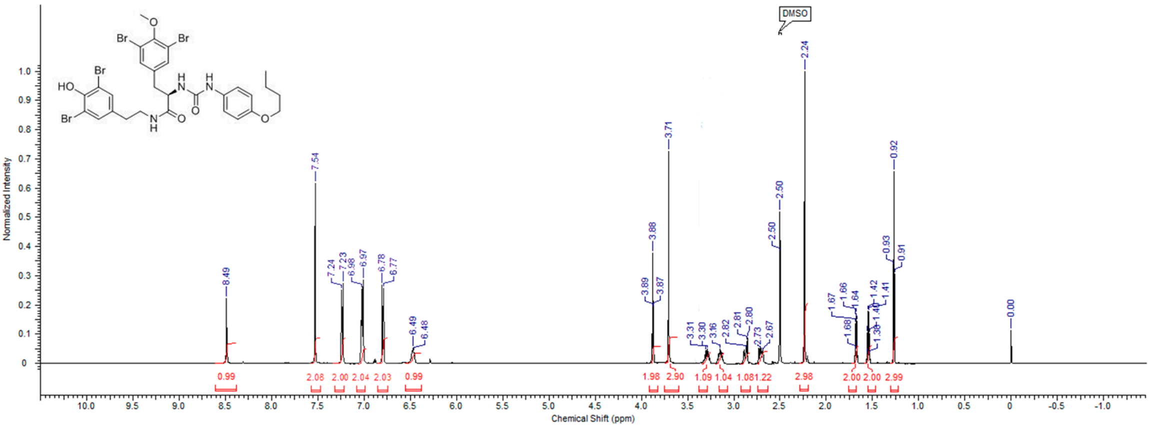


**5D $^{1}$H-NMR**

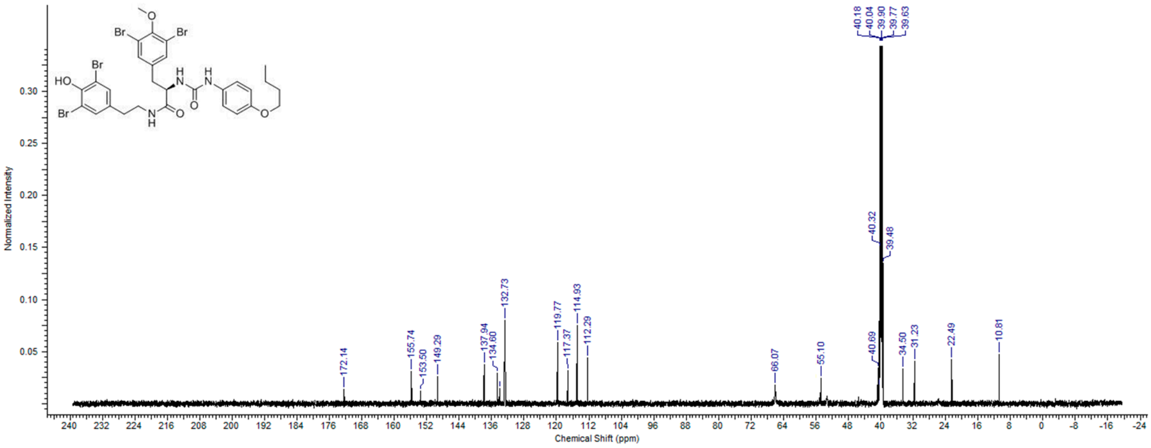


**5D $^{13}$C-NMR**

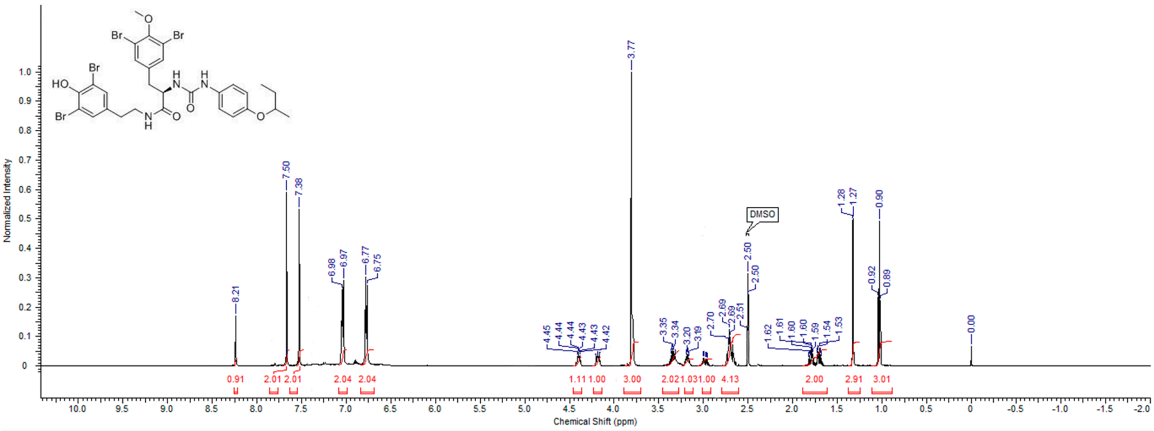


**5E $^{1}$H-NMR**

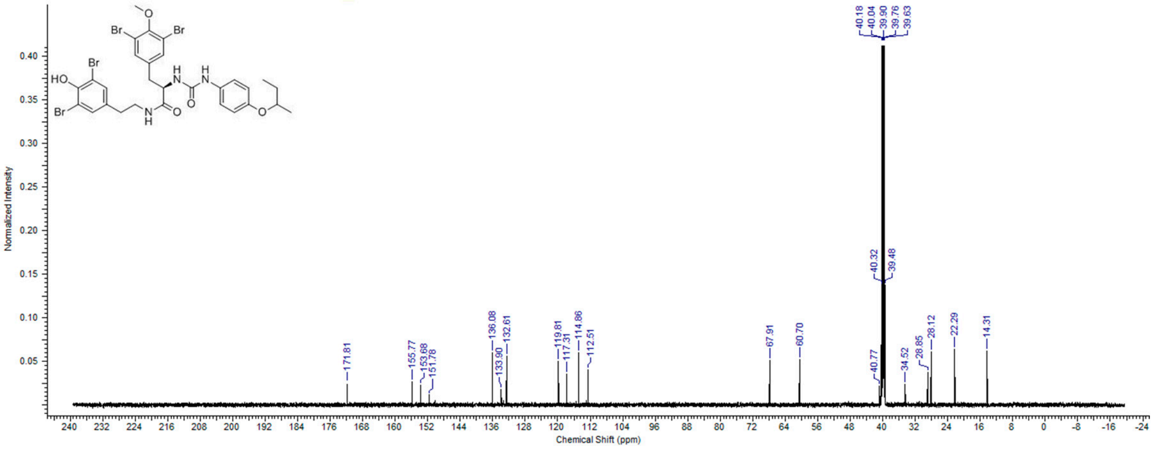


**5E $^{13}$C-NMR**

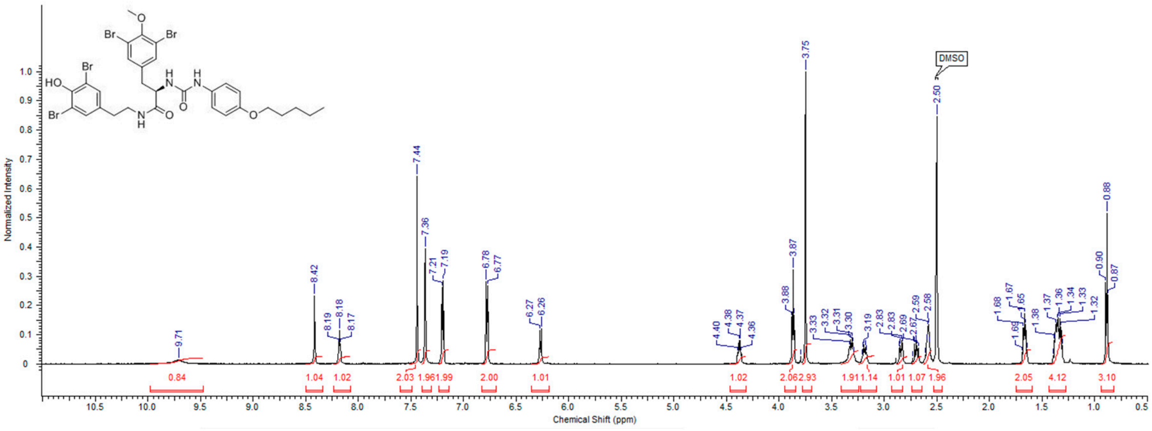


**5F $^{1}$H-NMR**

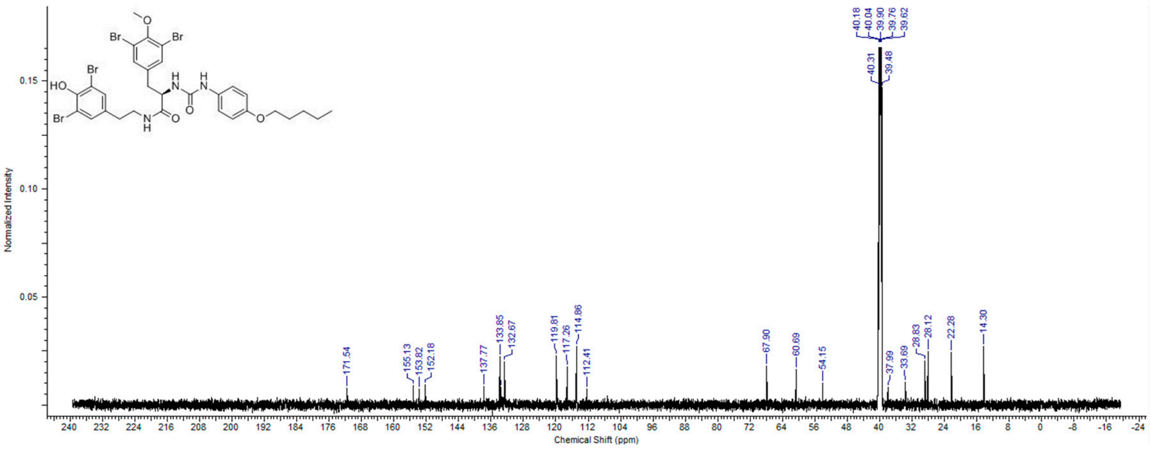


**5F $^{13}$C-NMR**

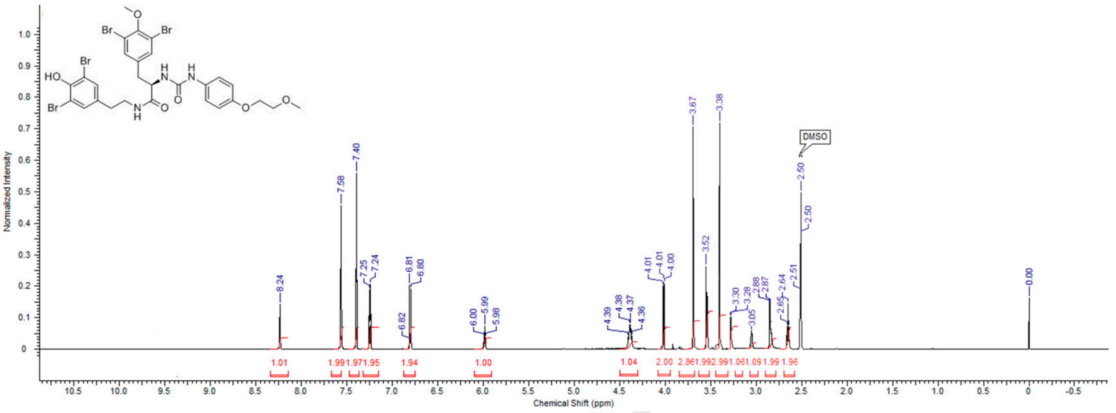


**5G $^{1}$H-NMR**

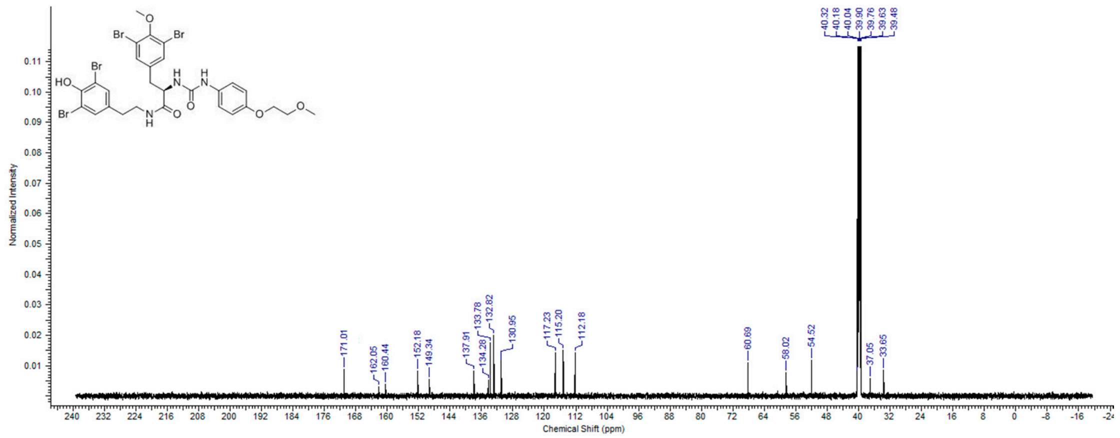

**5G $^{13}$C-NMR**

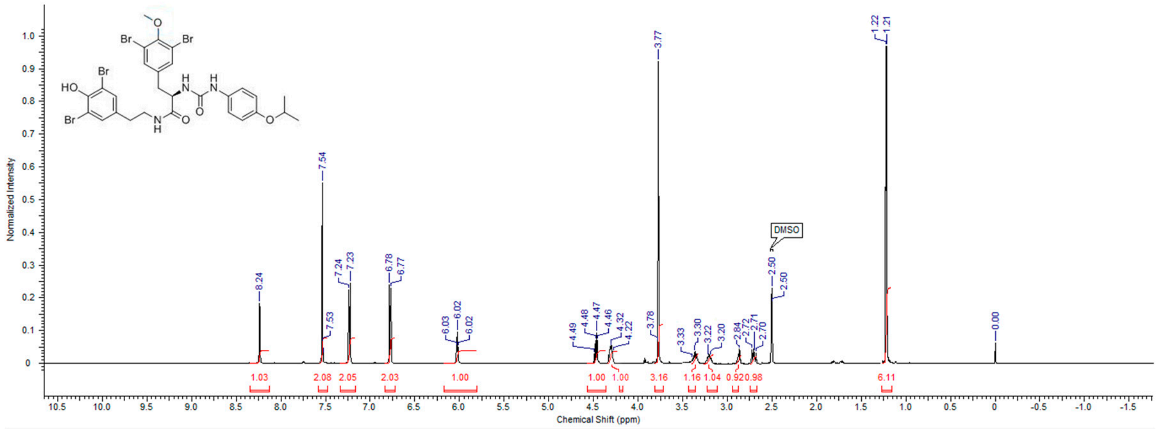

**5H $^{1}$H-NMR**

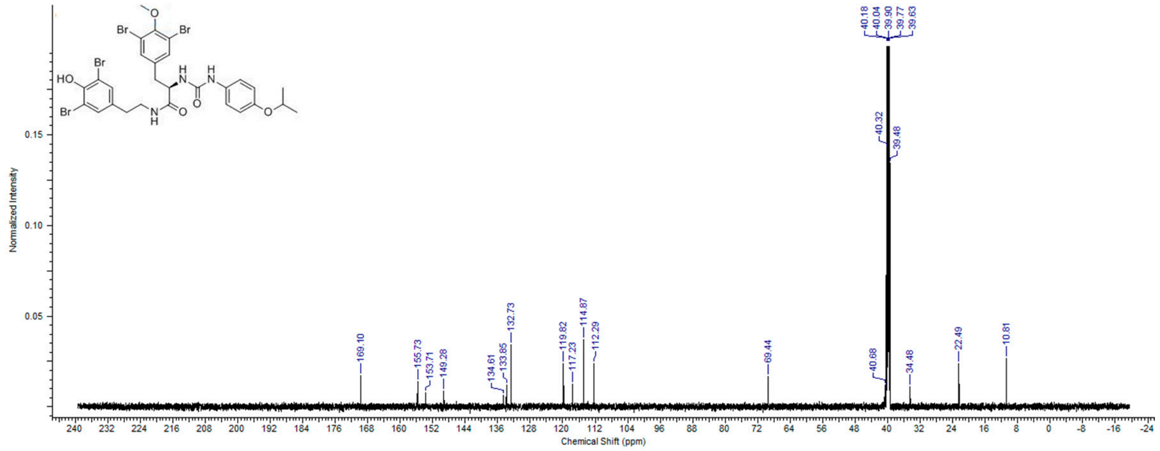

**5H $^{13}$C-NMR**

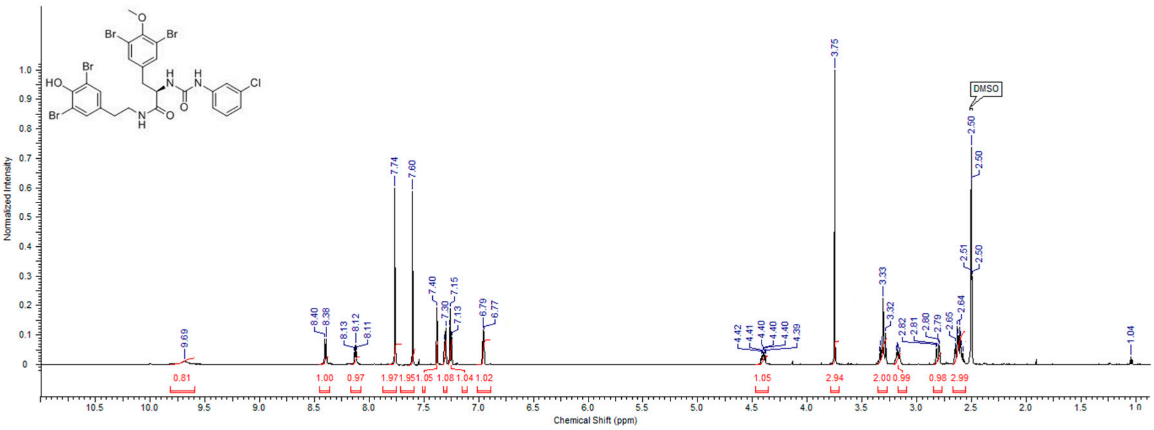


**5I $^{1}$H-NMR**

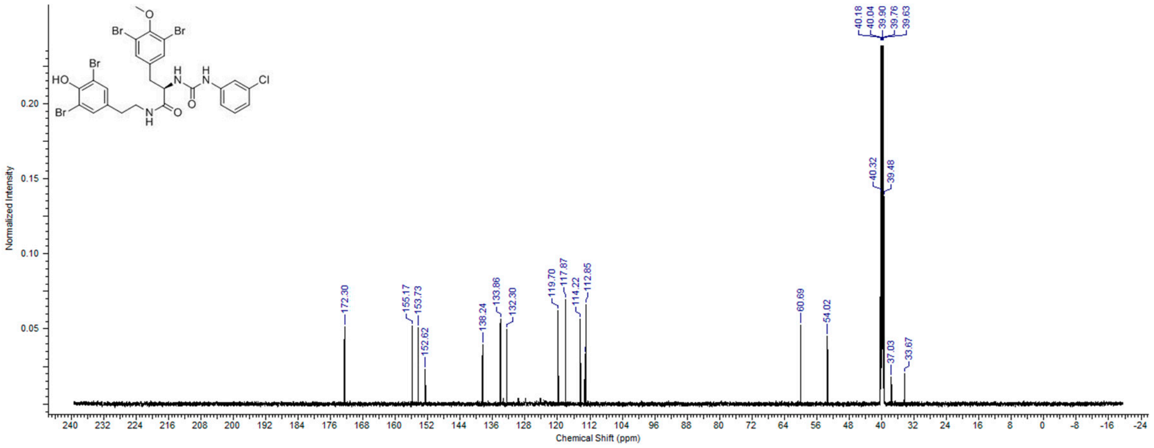


**5I $^{13}$C-NMR**

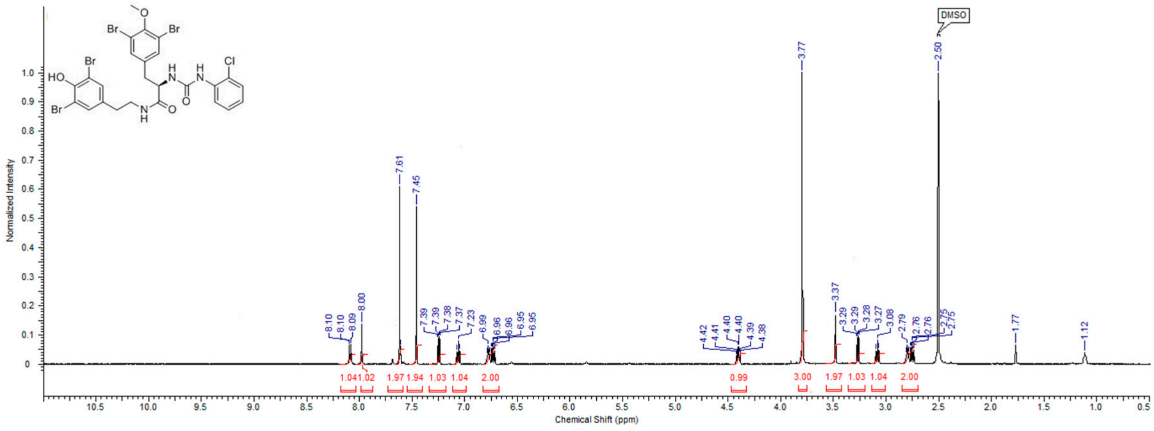


**5J $^{1}$H-NMR**

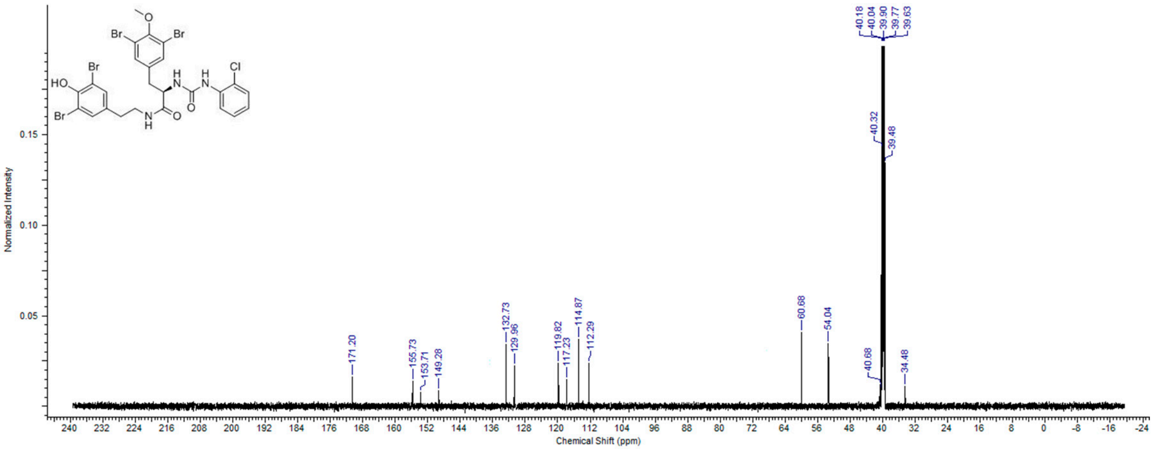


**5J $^{13}$C-NMR**

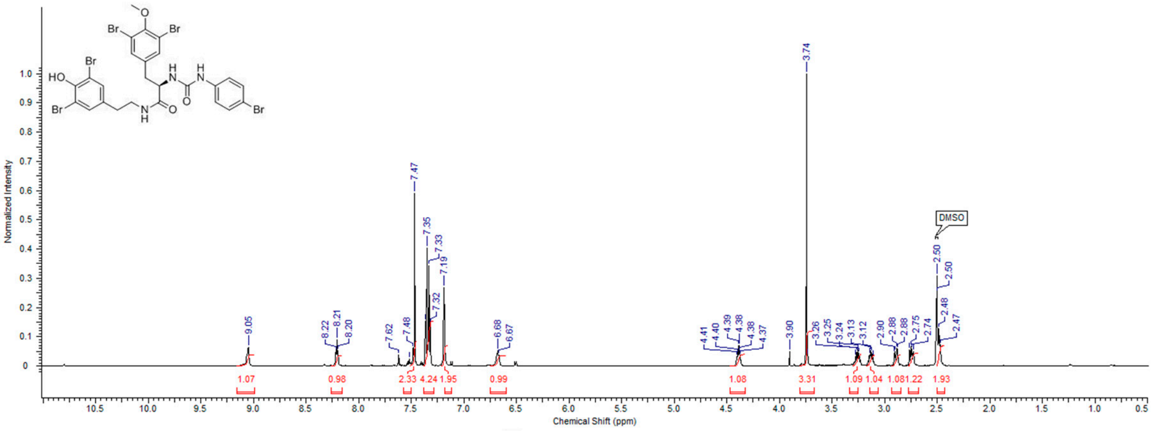


**5K $^{1}$H-NMR**

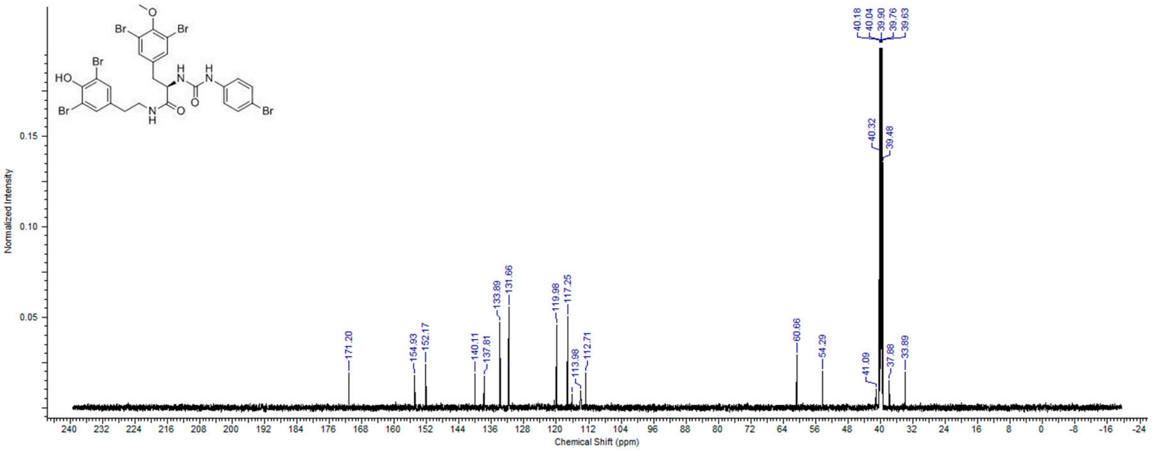


**5K $^{13}$C-NMR**

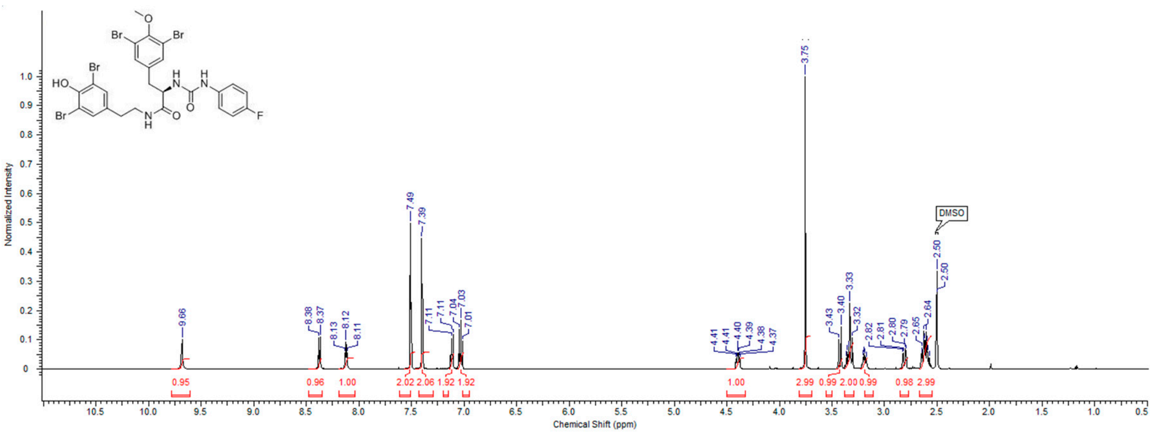


**5L $^{1}$H-NMR**

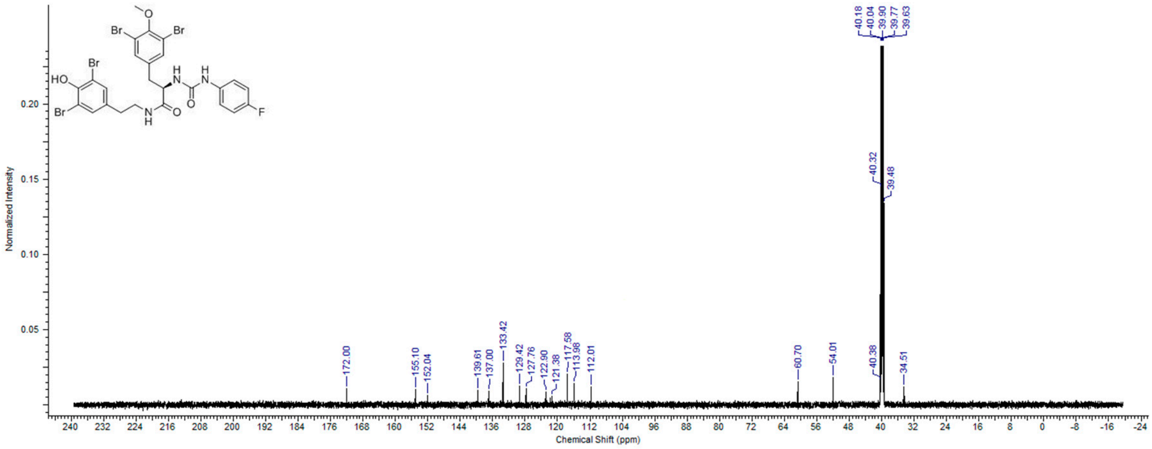


**5L $^{13}$C-NMR**

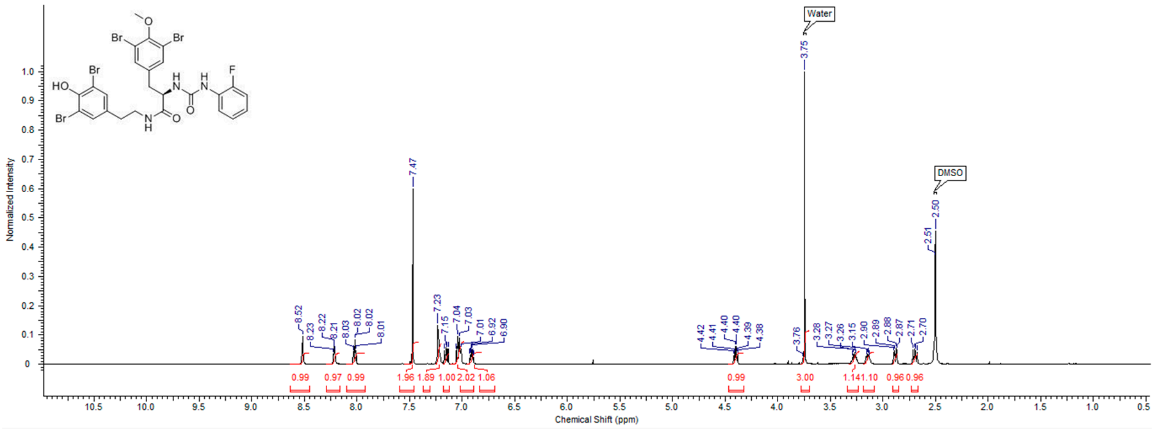


**5M $^{1}$H-NMR**

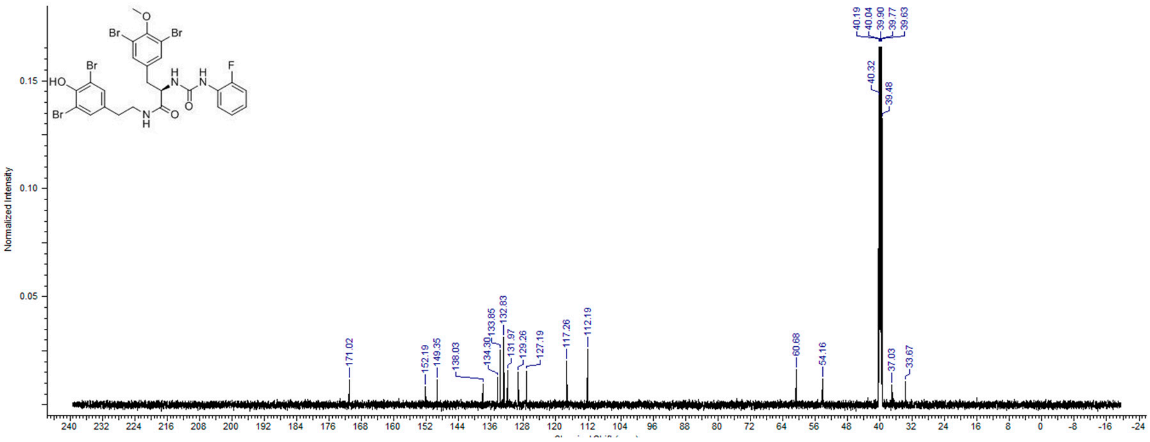


**5M $^{13}$C-NMR**

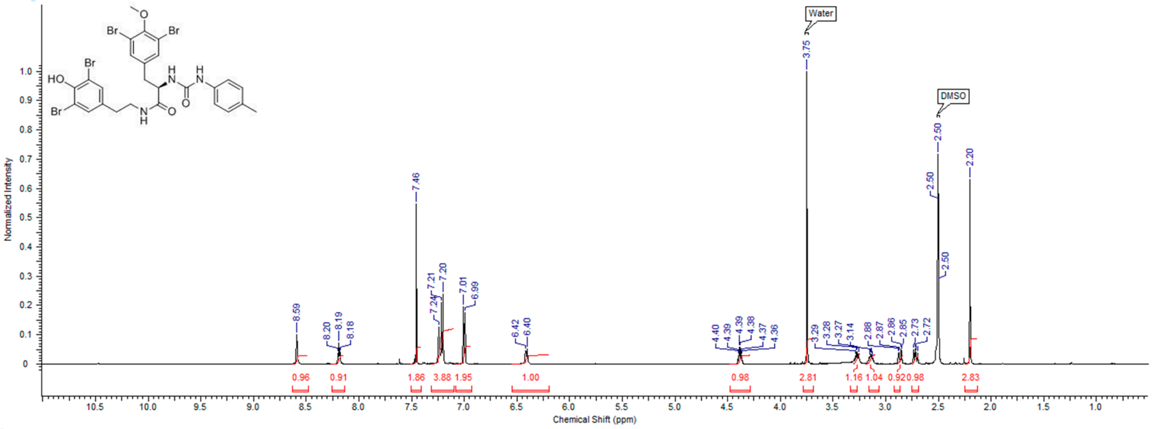


**5N $^{1}$H-NMR**

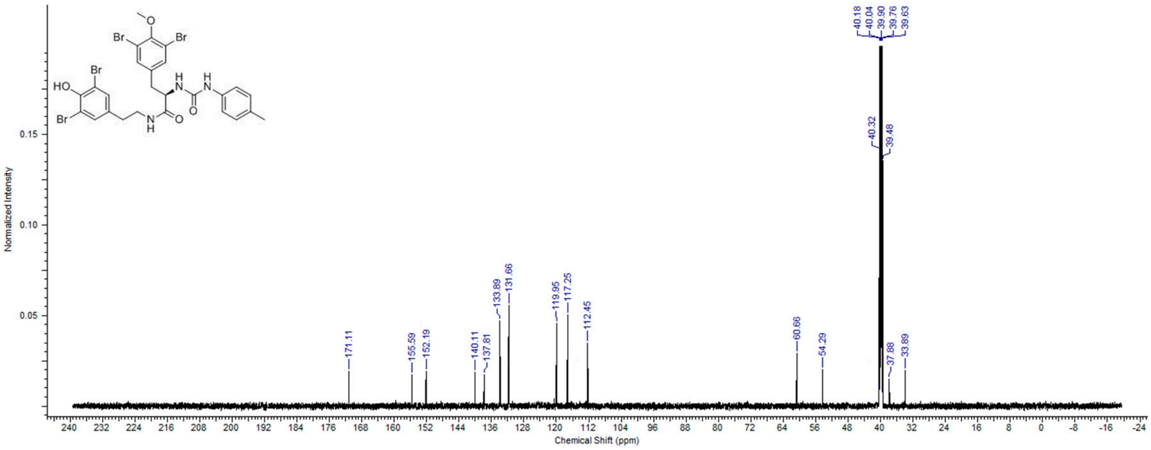


**5N $^{13}$C-NMR**

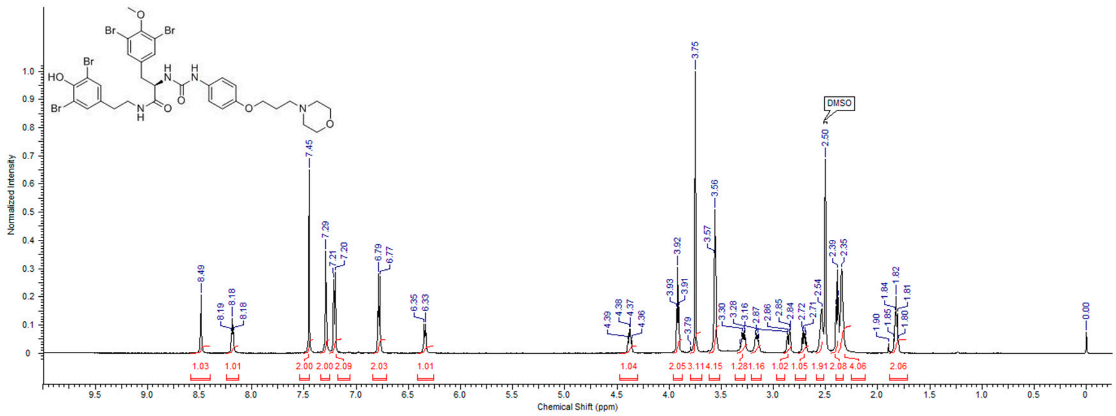


**5O $^{1}$H-NMR**

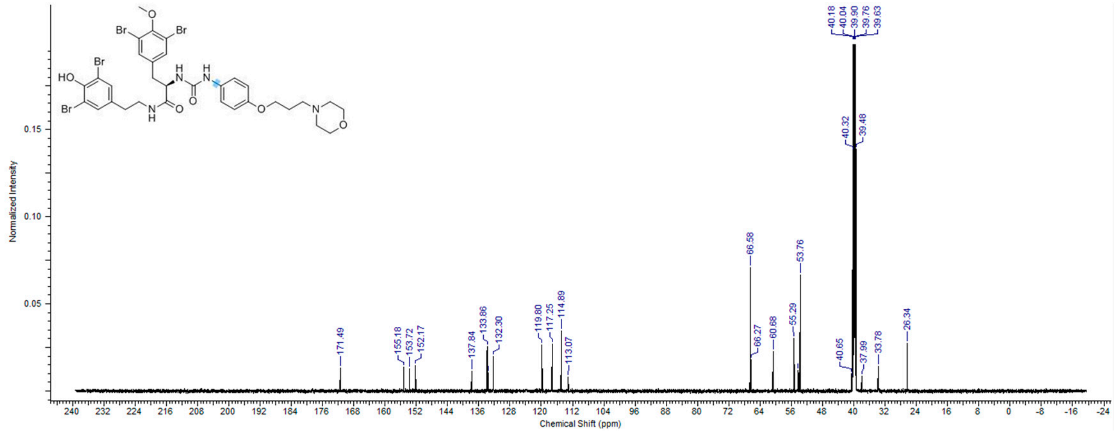


**5O $^{13}$C-NMR**

***2.3***

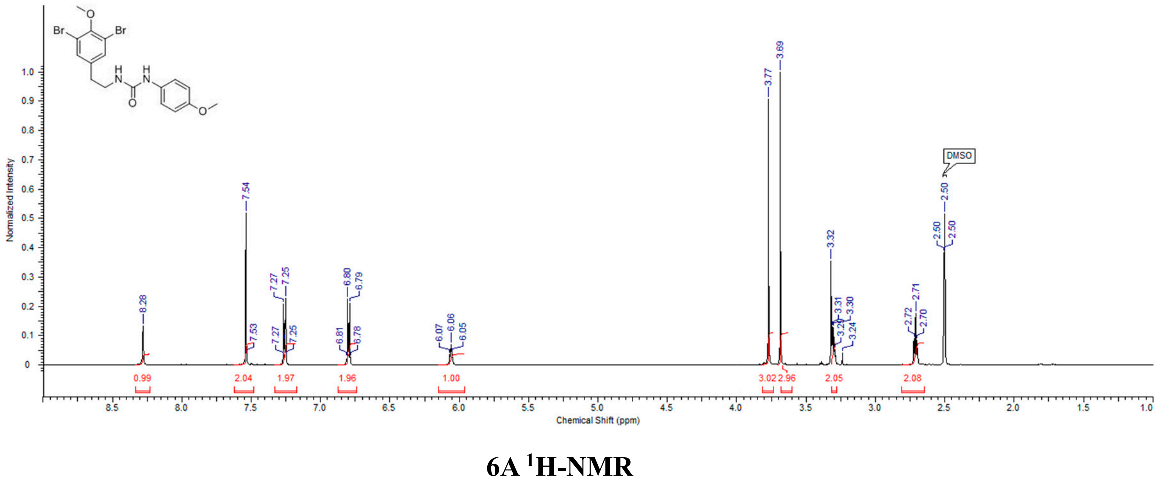


**6A $^{1}$H-NMR**

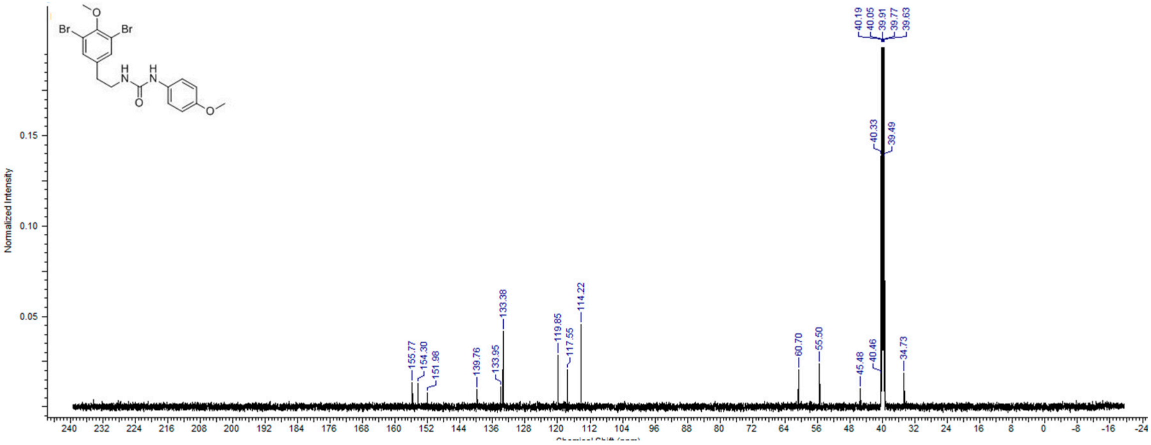


6A $^{13}$C-NMR

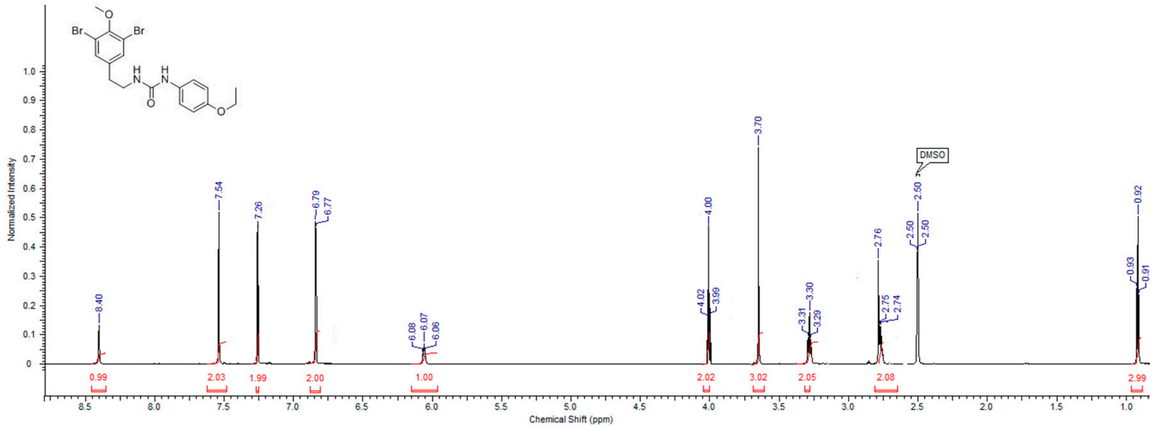


6B $^{1}$H-NMR

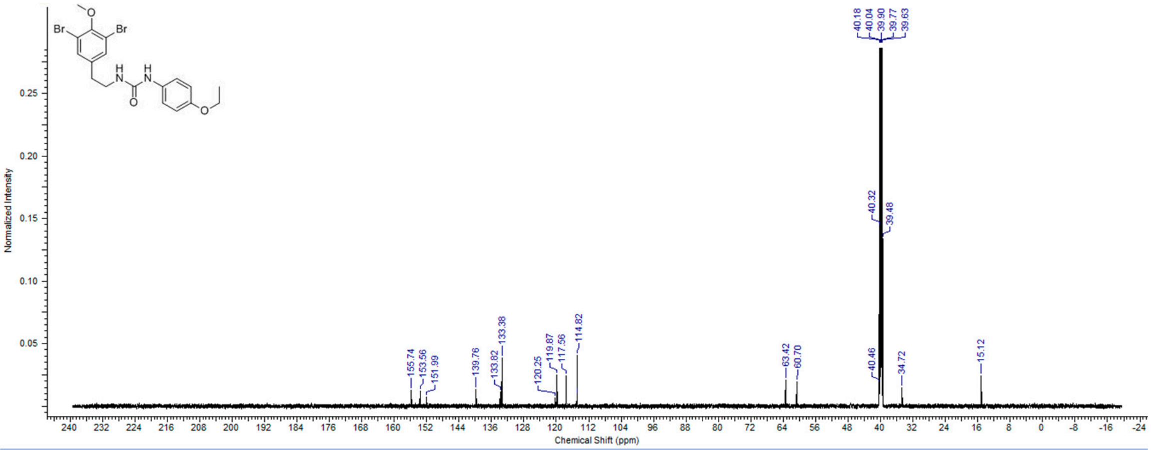


6B $^{13}$C-NMR

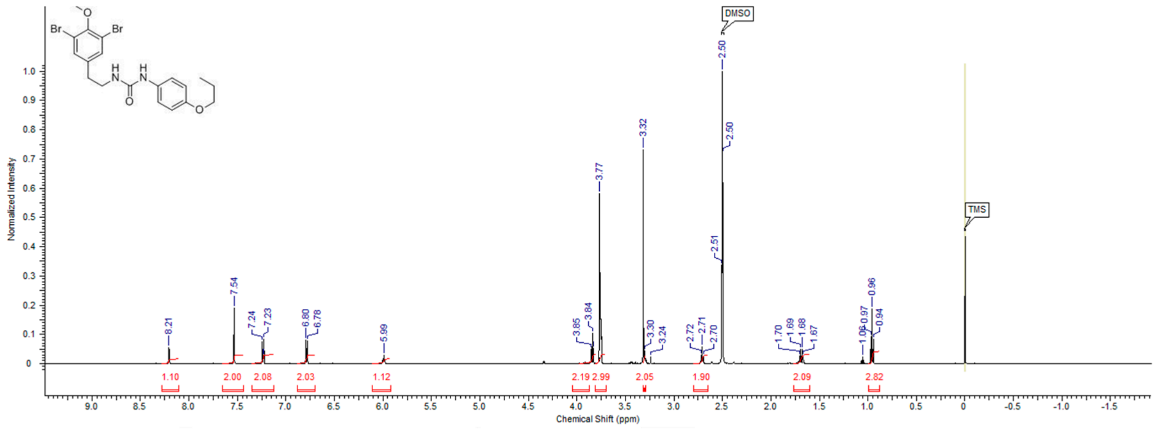

**6C $^{1}$H-NMR**

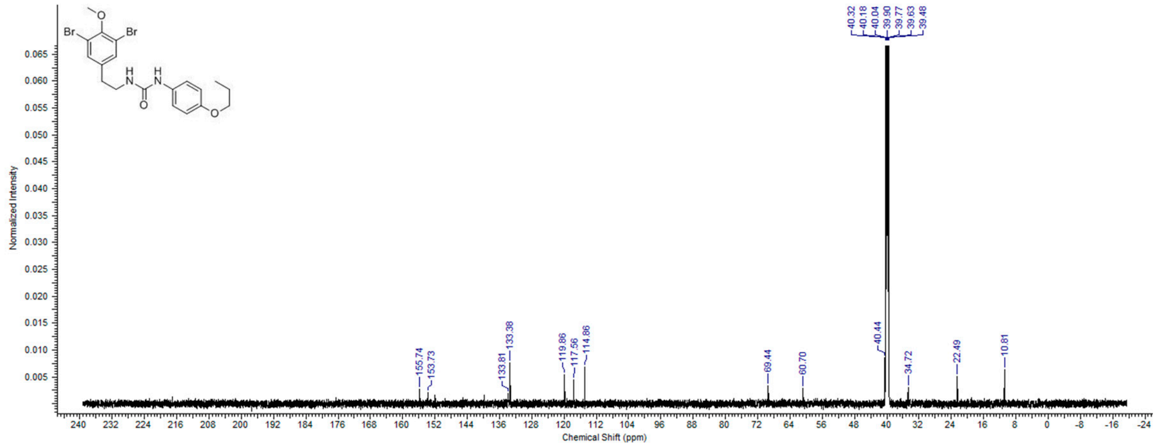

**6C $^{13}$C-NMR**

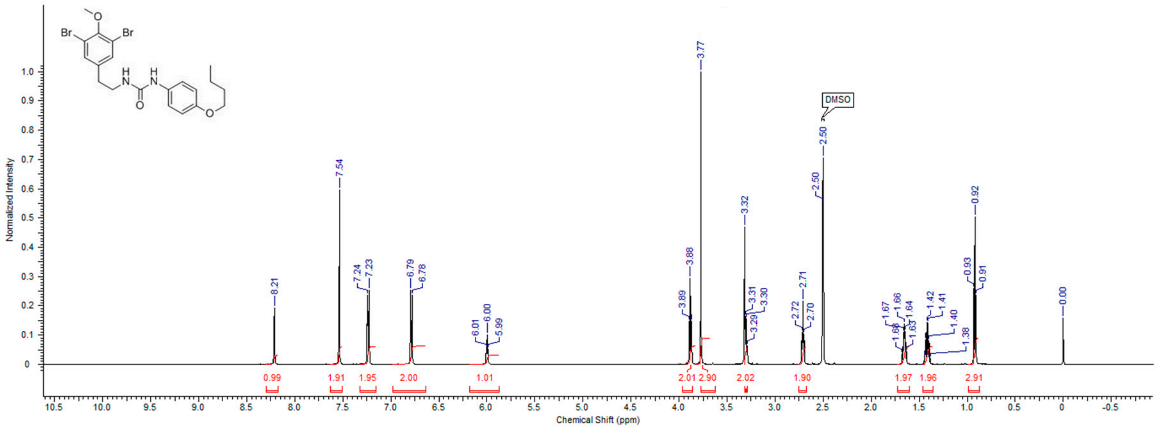

**6D $^{1}$H-NMR**

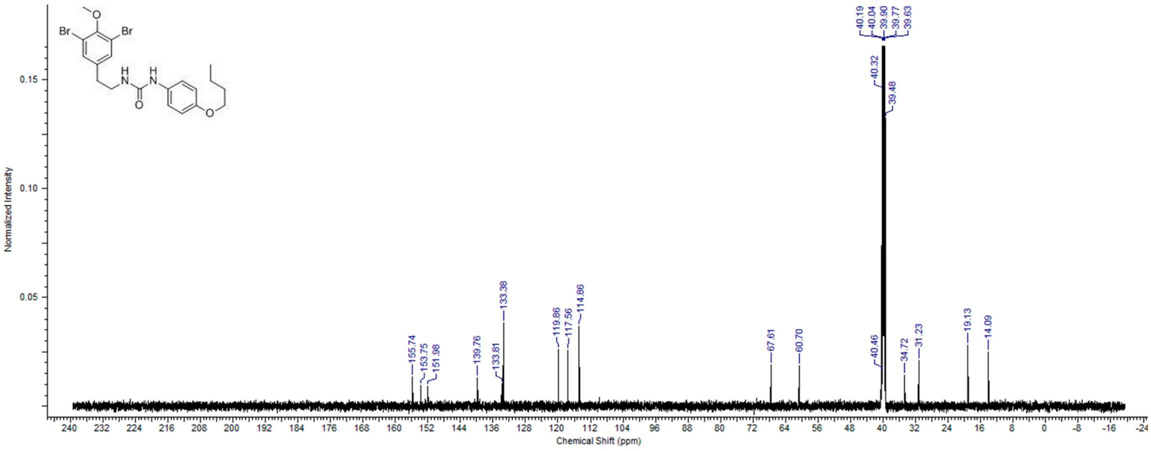


6D $^{13}$C-NMR

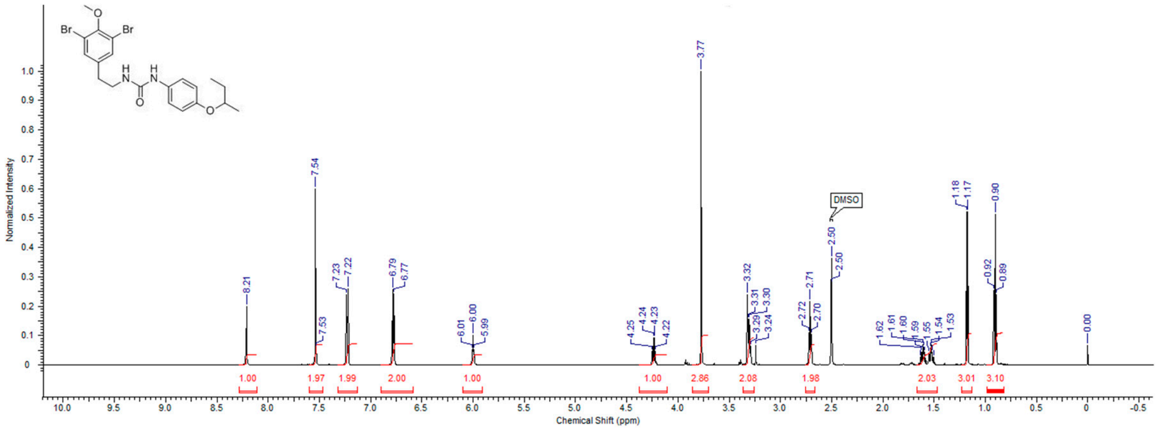


6E $^{1}$H-NMR

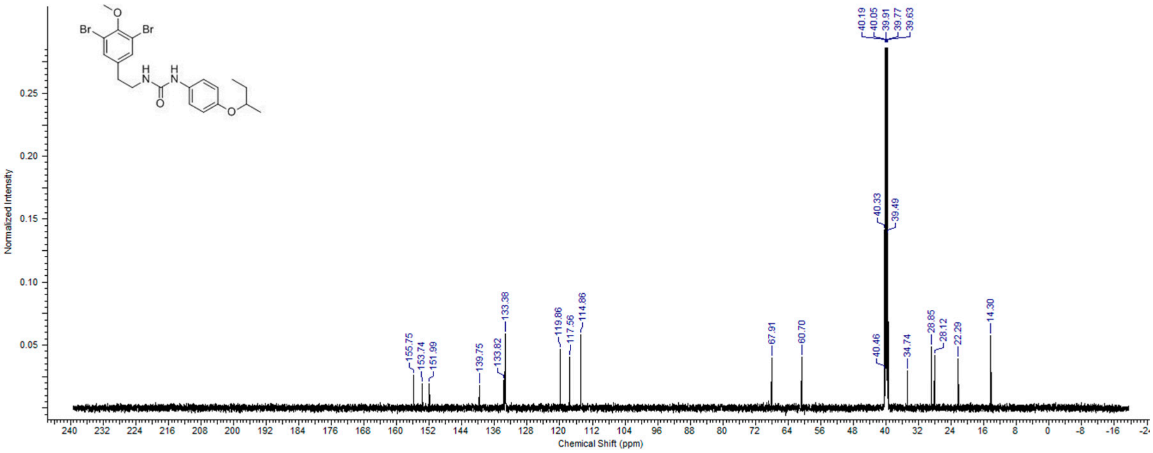


6E $^{13}$C-NMR

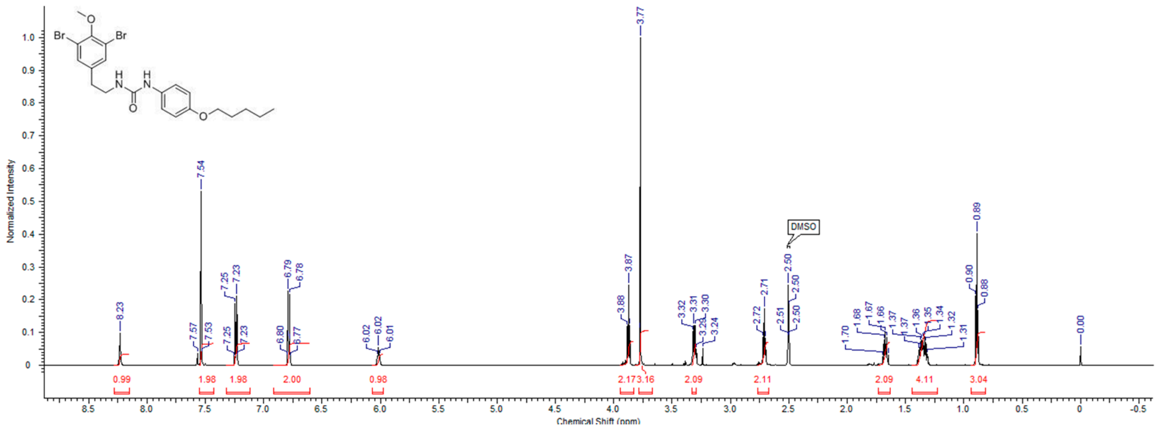

**6F $^{1}$H-NMR**

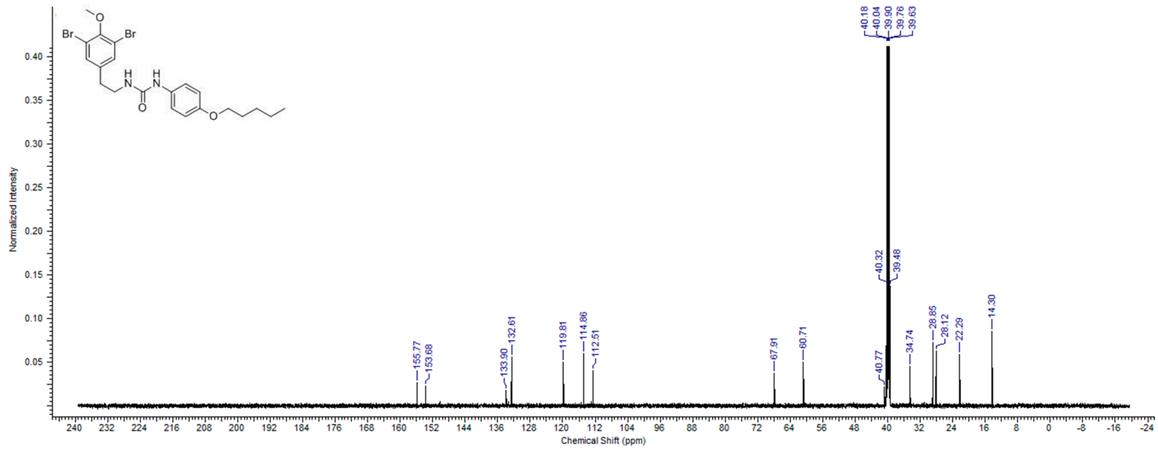

**6F $^{13}$C-NMR**

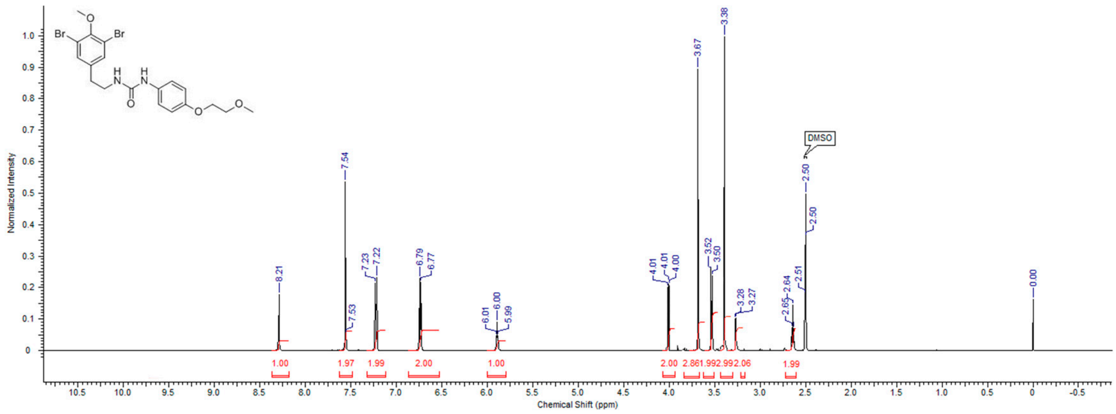

**6G $^{1}$H-NMR**

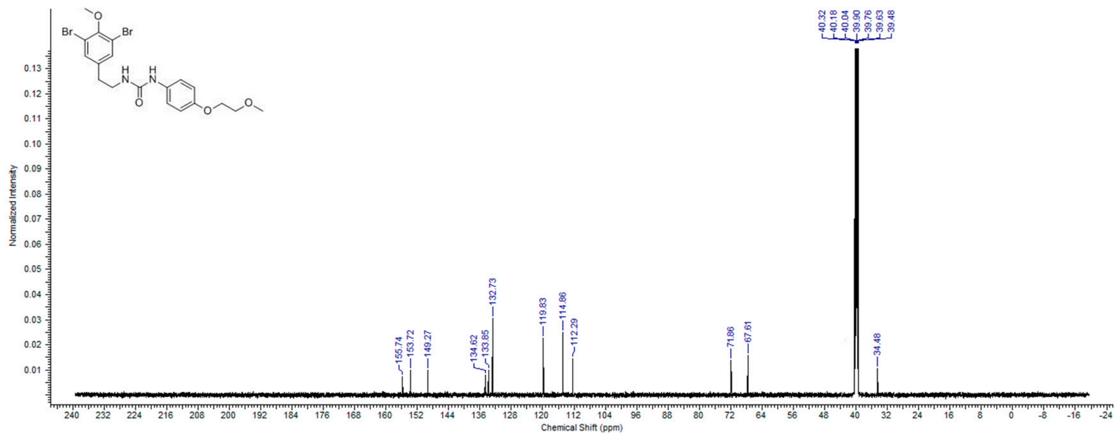

**6G $^{13}$C-NMR**

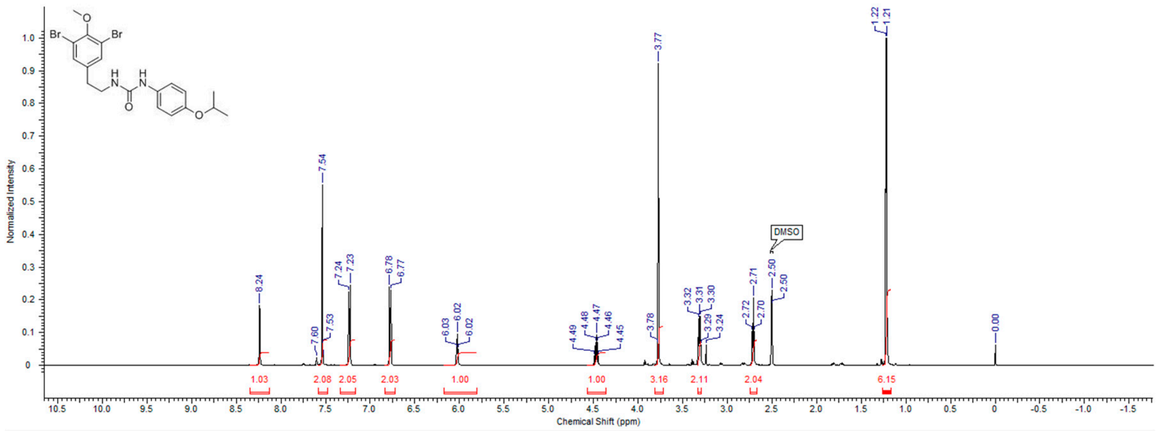

**6H $^{1}$H-NMR**

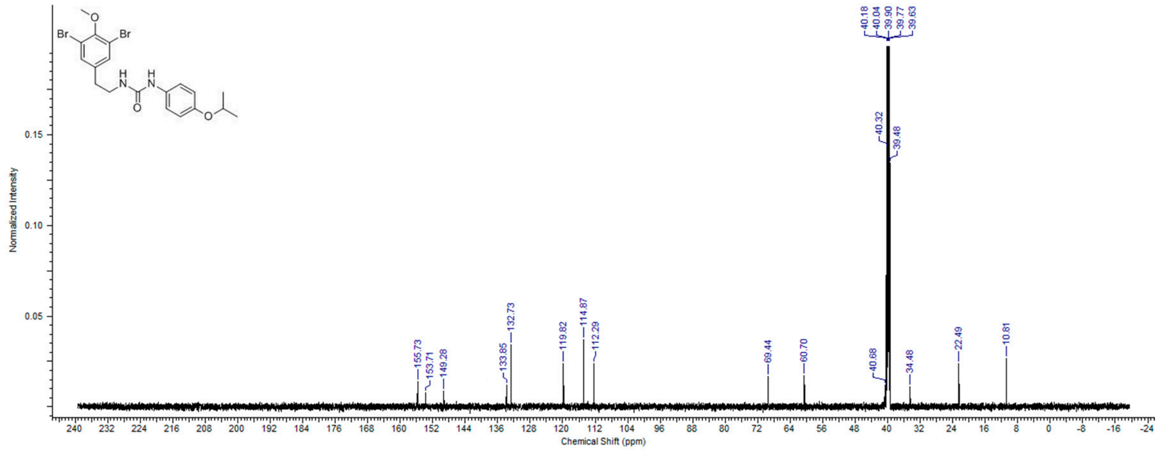

**6H $^{13}$C-NMR**

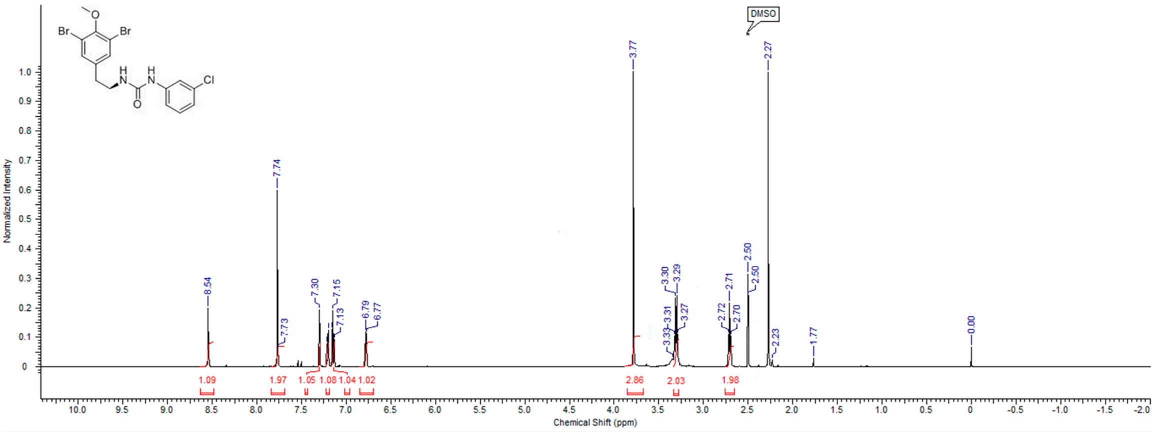


6I $^{1}$H-NMR

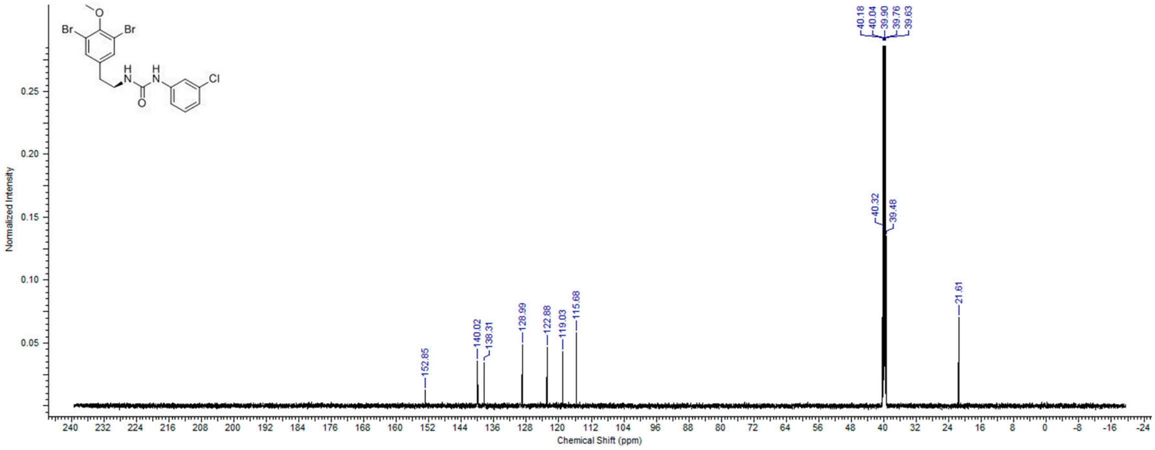


6I $^{13}$C-NMR

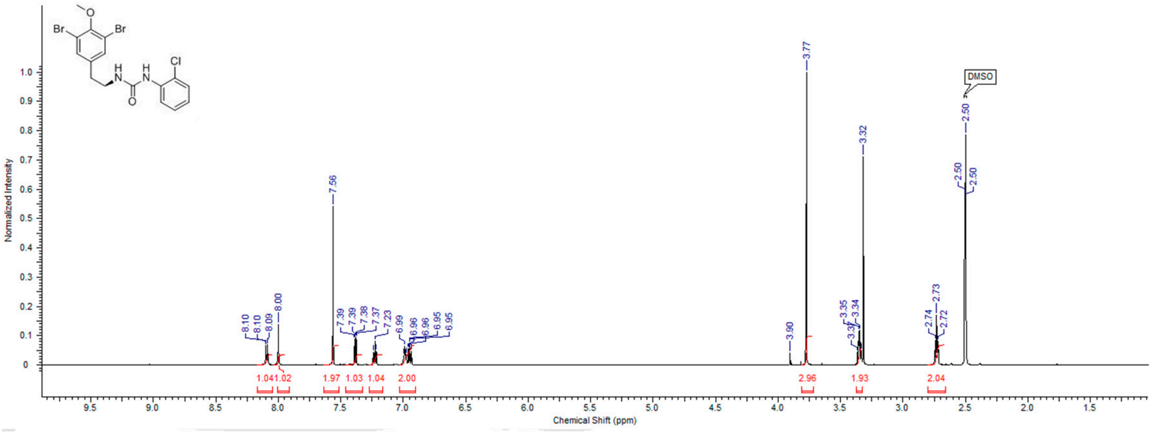


6J $^{1}$H-NMR

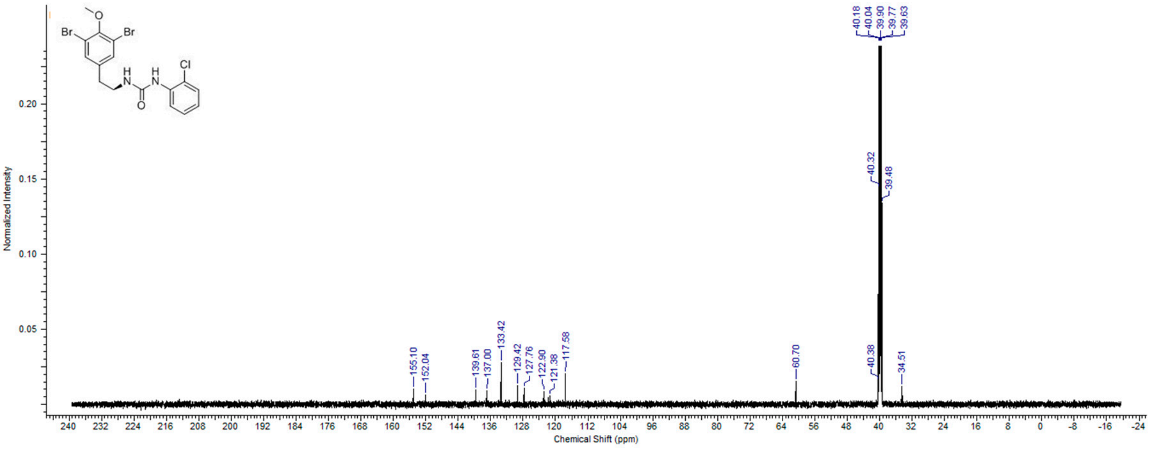


**6J $^{13}$C-NMR**

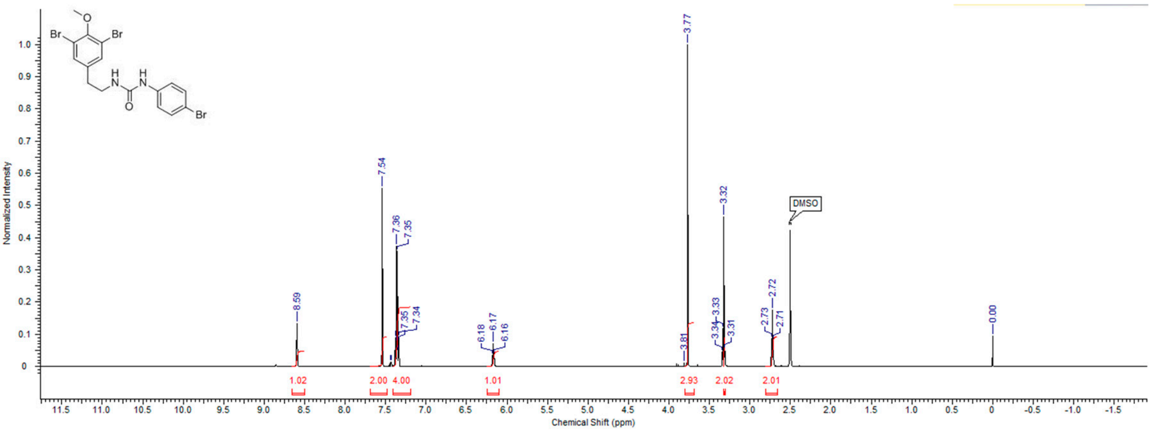


**6K $^{1}$H-NMR**

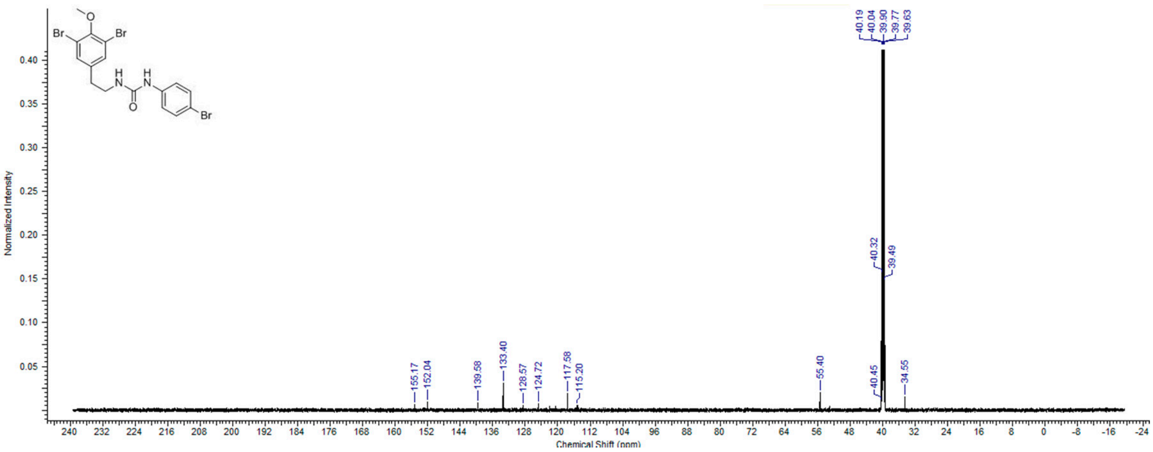


**6K $^{13}$C-NMR**

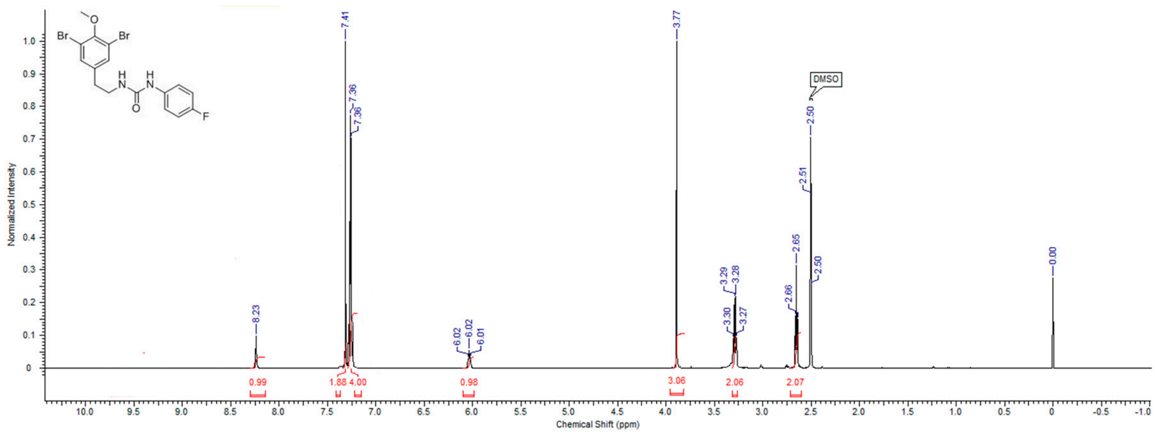

**6L $^{1}$H-NMR**

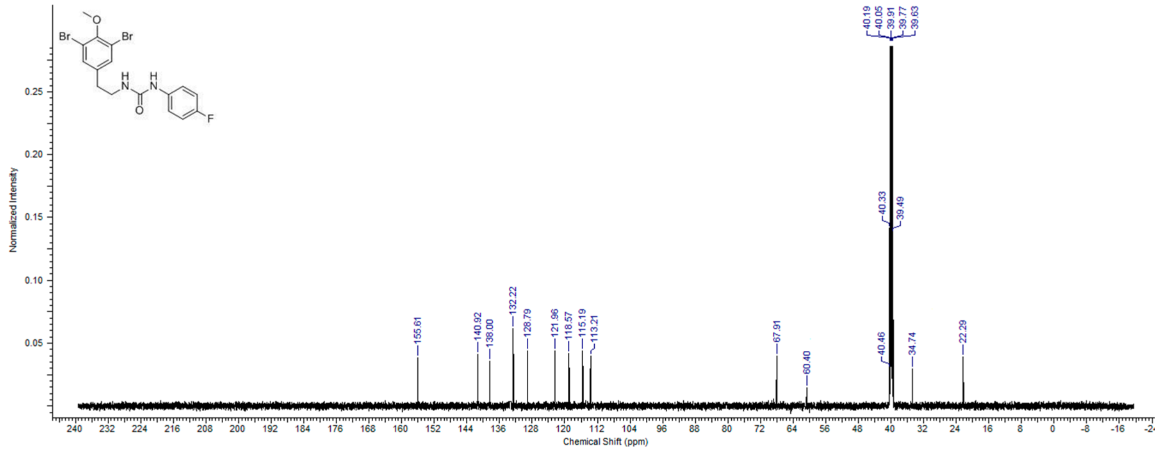

**6L $^{13}$C-NMR**

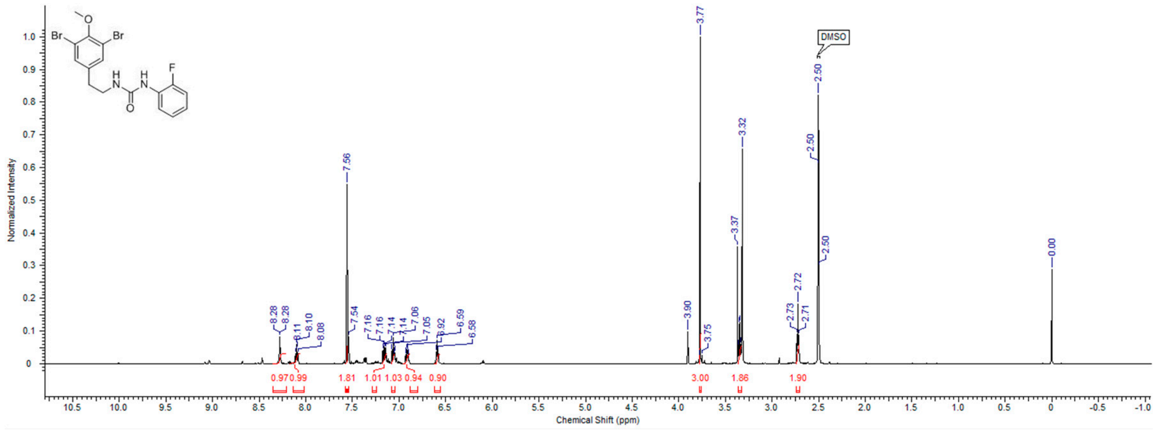

**6M $^{1}$H-NMR**

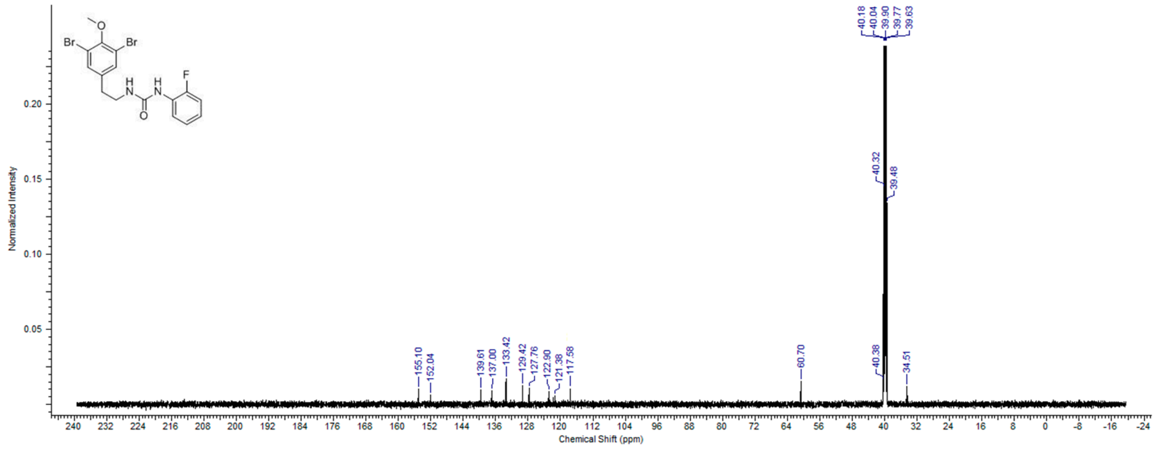


**6M $^{13}$C-NMR**

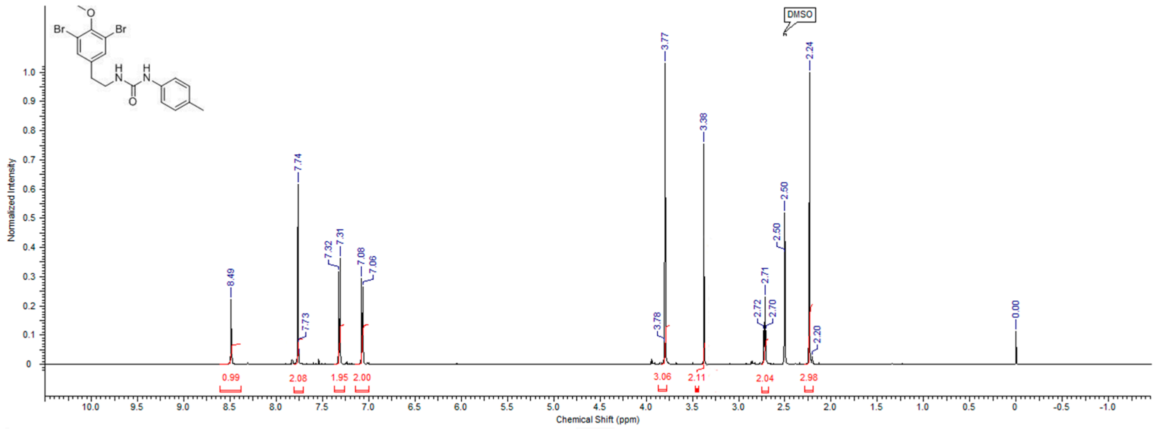


**6N $^{1}$H-NMR**

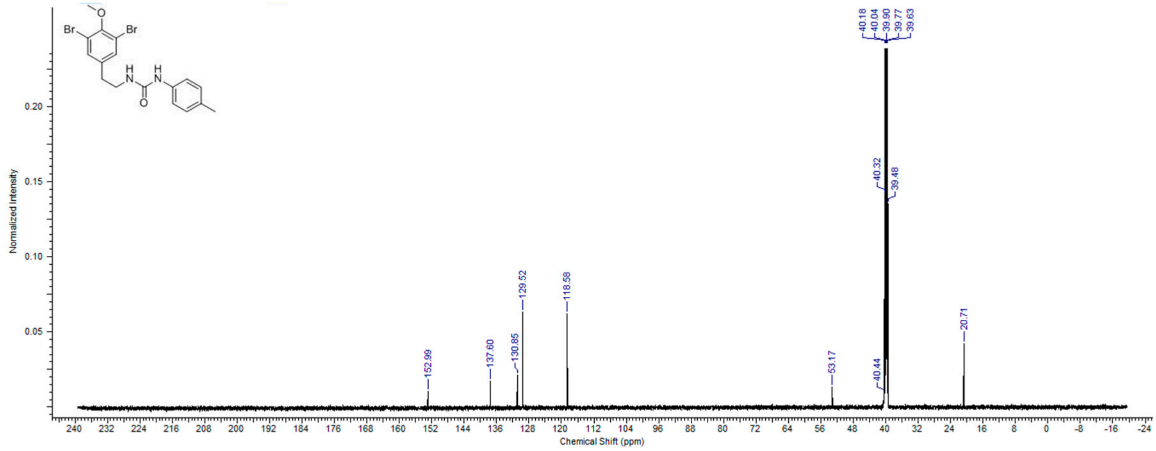


**6N $^{13}$C-NMR**

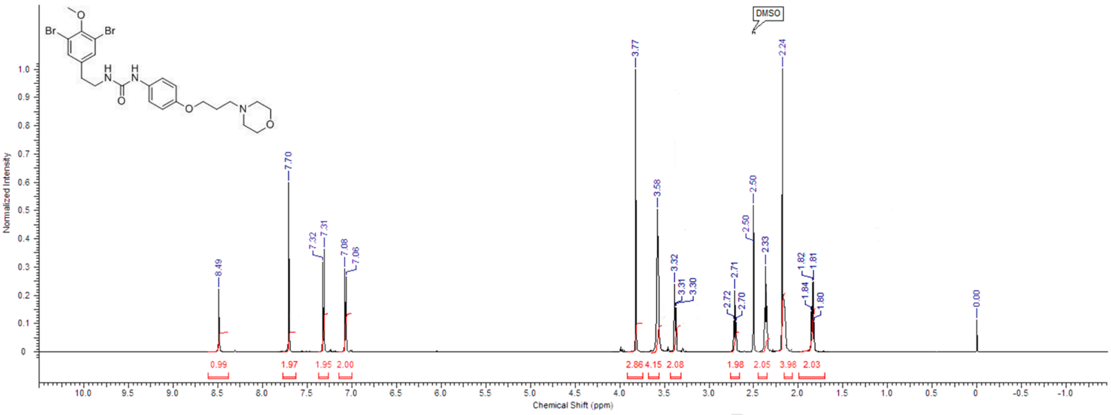


**6O $^{1}$H-NMR**

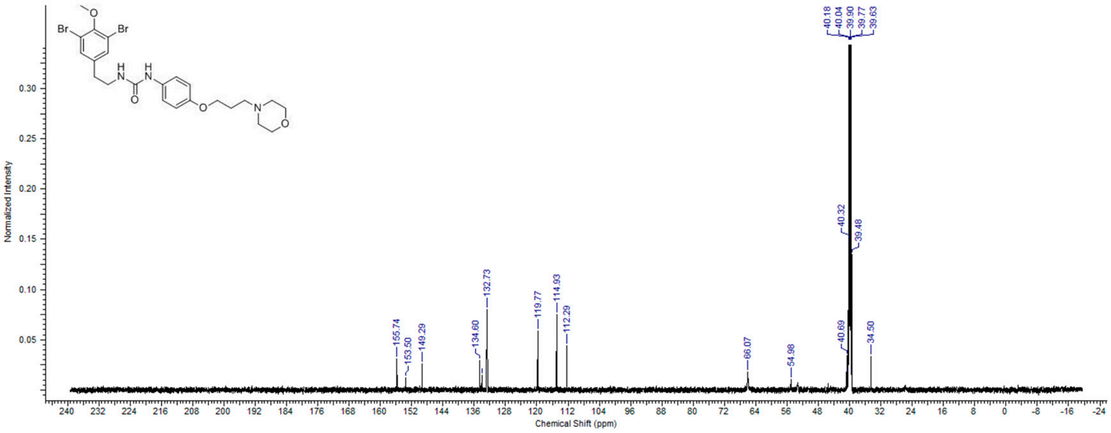


**6O $^{13}$C-NMR**

*4.2 HPLC data of 6O*

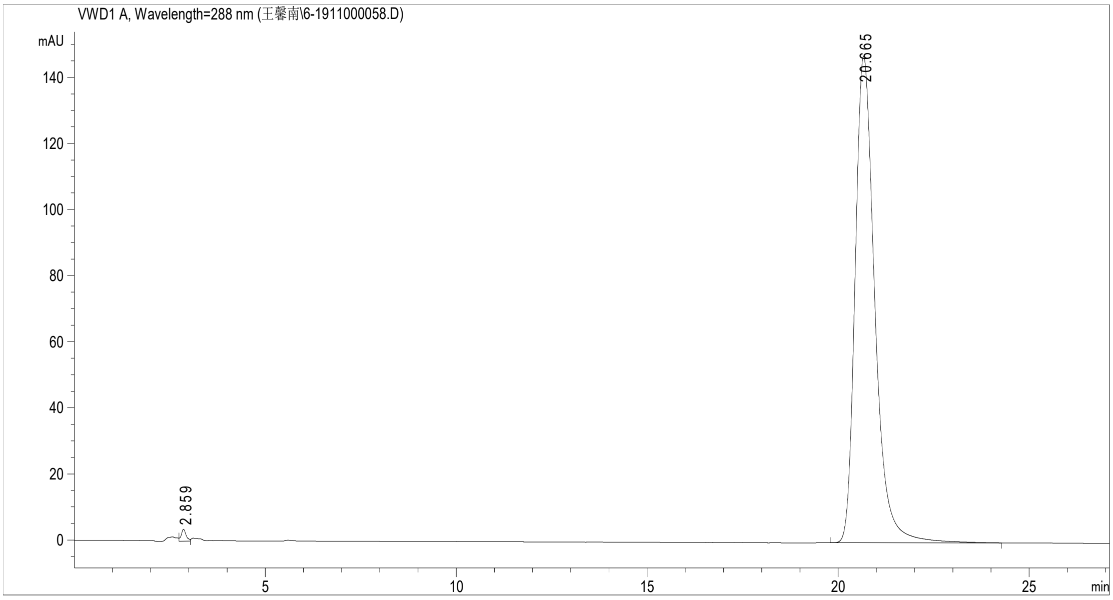


**HPLC data for the purity of 6O (MeOH:$H_2O$=1:1)**
